# KuINins as a New Class of HIV-1 Inhibitors That Block Post-Integration DNA Repair

**DOI:** 10.3390/ijms242417354

**Published:** 2023-12-11

**Authors:** Andrey Anisenko, Simon Galkin, Andrey A. Mikhaylov, Maria G. Khrenova, Yulia Agapkina, Sergey Korolev, Lidia Garkul, Vasilissa Shirokova, Viktoria A. Ikonnikova, Alexander Korlyukov, Pavel Dorovatovskii, Mikhail Baranov, Marina Gottikh

**Affiliations:** 1 Chemistry Department, Lomonosov Moscow State University, 119992 Moscow, Russia; khrenova.maria@gmail.com (M.G.K.); agapkina@belozersky.msu.ru (Y.A.); spkorolev@mail.ru (S.K.); 2 Faculty of Bioengineering and Bioinformatics, Lomonosov Moscow State University, 119992 Moscow, Russia; simon.galkin@gmail.com (S.G.); lidia.garkul@yandex.ru (L.G.); 3 Belozersky Institute of Physico-Chemical Biology, Lomonosov Moscow State University, 119992 Moscow, Russia; 4 Shemyakin-Ovchinnikov Institute of Bioorganic Chemistry of the Russian Academy of Sciences, 117997 Moscow, Russiashirokova.vasilissa@gmail.com (V.S.); victoriaikonnikova@yandex.ru (V.A.I.); baranovmikes@gmail.com (M.B.); 5 Federal Research Centre of Biotechnology, Russian Academy of Sciences, 119071 Moscow, Russia; 6 Higher Chemical College, D.I. Mendeleev University of Chemical Technology of Russia, 125047 Moscow, Russia; 7 Nesmeyanov Institute of Organoelement Compounds, 119334 Moscow, Russia; alex@xrlab.ineos.ac.ru; 8 Institute of Translational Medicine and Institute of Pharmacy and Medicinal Chemistry, Pirogov Russian National Research Medical University, 117997 Moscow, Russia; 9 National Research Center “Kurchatov Institute”, 123098 Moscow, Russia; paulgemini@mail.ru

**Keywords:** HIV-1, integrase, Ku70, DNA-PK, post-integration repair, inhibitor of protein–protein interaction, KuINins

## Abstract

Integration of HIV-1 genomic cDNA results in the formation of single-strand breaks in cellular DNA, which must be repaired for efficient viral replication. Post-integration DNA repair mainly depends on the formation of the HIV-1 integrase complex with the Ku70 protein, which promotes DNA-PK assembly at sites of integration and its activation. Here, we have developed a first-class inhibitor of the integrase-Ku70 complex formation that inhibits HIV-1 replication in cell culture by acting at the stage of post-integration DNA repair. This inhibitor, named s17, does not affect the main cellular function of Ku70, namely its participation in the repair of double-strand DNA breaks through the non-homologous end-joining pathway. Using a molecular dynamics approach, we have constructed a model for the interaction of s17 with Ku70. According to this model, the interaction of two phenyl radicals of s17 with the L76 residue of Ku70 is important for this interaction. The requirement of two phenyl radicals in the structure of s17 for its inhibitory properties was confirmed using a set of s17 derivatives. We propose to stimulate compounds that inhibit post-integration repair by disrupting the integrase binding to Ku70 KuINins.

## 1. Introduction

HIV integrase (IN) is one of the major proteins in the HIV-1 life cycle. It catalyzes the integration of viral cDNA into the cellular genomic DNA. In addition to its primary function, IN interacts with many cellular proteins essential for efficient virus replication [1,2,3]. Interestingly, these proteins regulate integration as well as reverse transcription [3,4], maintaining optimal capsid stability [5], integration site selection [6,7,8], and even post-integration DNA repair (PIR) [9] and transition from integration to early transcription [10]. Complexes between IN and its cellular partner proteins are considered promising targets for the development of novel drugs with a reduced risk of resistant strains’ emergence [2,3]. 

The cellular transcription co-activator LEDGF/p75 is the most studied cellular partner of IN [11]. This protein protects IN from proteasomal degradation [12] and directs viral DNA integration into actively transcribed regions of the cellular genome [6,13,14]. Compounds called LEDGINs or ALLINIs preventing LEDGF/p75 binding to IN and inhibiting viral replication have been developed [15,16,17]. However, further study of such inhibitors has shown that they predominantly inhibit viral replication by disrupting IN multimerization and proper virion morphogenesis [18,19]. Now they are undergoing preclinical trials [20]. 

We have focused on the IN complex with a protein that regulates PIR. Integration of viral cDNA into the cellular genome results in the formation of single-stranded 5-nucleotide gaps in the cellular DNA, which flank the integrated viral DNA (the so called integration intermediate), as well as unpaired CA dinucleotides at the 5’-ends of the viral DNA [21]. Successful viral replication requires the repair of these DNA damages.

Previously, we have shown that the DNA-PK complex and its kinase activity are necessary for efficient PIR [9]. DNA-PK consists of a heterodimer Ku70/Ku80 and a catalytic subunit DNA-PKcs. The main cellular function of DNA-PK is to participate in double-strand break (DSB) repair via non-homologous end joining (NHEJ) [22]. The Ku heterodimer in this process first binds DNA ends and then recruits DNA-PKcs, which holds both ends of the damaged DNA together and also recruits and phosphorylates downstream proteins [22]. However, the HIV-1 integration intermediate does not contain DSB; therefore, both the accumulation of DNA-PK on the integration intermediate and DNA-PK activation occur due to the formation of the IN/Ku70 complex [9,23]. PIR can be impaired by DNA-PKcs inhibition using Nu7441, but this inhibitor also affects the efficiency of cellular DBS repair. Another approach to PIR inhibition is to prevent the formation of the IN/Ku70 complex. This can be completed, for example, by amino acid substitutions at the interface of proteins’ interaction or by low-molecular-weight inhibitors of this interaction. The first approach has been implemented in [9], whereas the second has not yet been used. 

Here, we have developed a first-class inhibitor that blocks HIV-1 replication at the stage of post-integration DNA repair. Using molecular docking of the 1.6M ChemDiv compound library into the IN binding pocket within Ku70, we selected 37 compounds for further testing. Three of them prevented the binding of IN to Ku70 in vitro. However, only one compound, named s17, inhibited the IN/Ku70 complex formation with relatively low IC50 = 12 μM. This compound inhibited the early stages of HIV-1 replication in 293T, Jurkat, and CEM cells. Importantly, s17 impaired post-integration DNA repair and did not affect the cellular NHEJ pathway of DSB repair.

## 2. Results

### 2.1. s17 Successfully Prevented HIV-1 Integrase Binding to Ku70 In Vitro

Previously, we have identified the binding site of HIV-1 integrase in the human Ku70 protein. It includes residues S69, I72, S73, and I76 of Ku70 [24]. They are located in the Ku70/Ku80 pocket suitable for molecular docking. Using this technique, we identified compounds Y021-2376 and 3272-0701 hindering the formation of the IN/Ku70 complex in vitro [24]. However, the high cytotoxicity of these compounds did not allow us to test their effect on HIV-1 replication. 

To find more potent inhibitors, we have implemented the following approach. A total SMILES format of 1.6M compounds’ structures was downloaded from the ChemDiv website. Next, compounds were filtered to meet the criteria presented in Table S1. After the filtering, we identified 1385 compounds that had a similarity Tanimoto score of at least 0.65 compared to the one of the previously identified Y021-2376 or 3272-0701 inhibitors [24]. These compounds were chosen for further docking analysis. After the docking, compounds with a minimal free binding energy less than the minimal free binding energy of the Y021-2376 inhibitor (−11.31 kcal/mol) were subjected to further analysis. The analysis involved the calculation of solvent accessible surface area (SASA) values of S69, I72, S73, and I76 residues for Ku complexes with each compound. Based on this scoring, 37 compounds with the highest rank were selected for further testing in vitro (Table S2).

**Figure 1:**
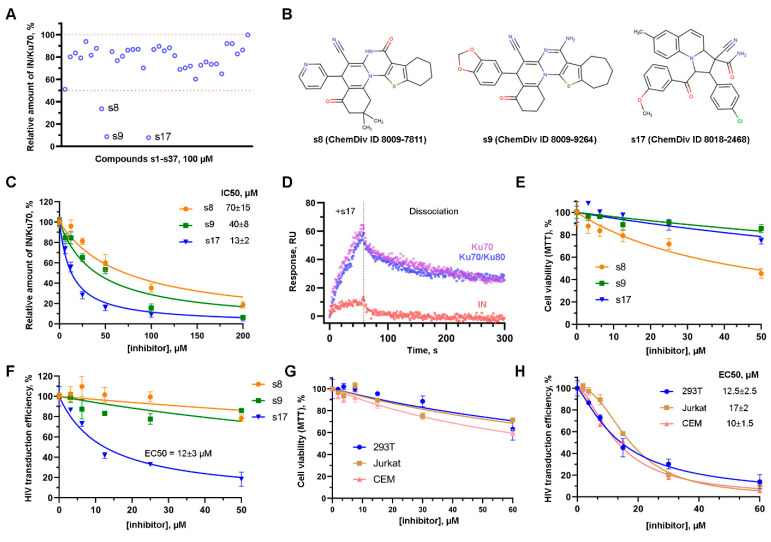
The effect of small molecules on the formation of the IN/Ku70 complex and on HIV-1 early replication events. (**A**) The effect of 100 μM compounds s1–s37 preselected with molecular docking procedure on the ability of Ku70 to bind HIV-1 IN in vitro. Mean values (*n* = 3) are shown. (**B**) Structures of the three most active compounds. (**C**) The effect of increasing concentrations of s8, s9, and s17 on the Ku70/IN complex formation in vitro. Mean values ± SD (*n* = 3) are shown. (**D**) SPR sensograms of the s17 interaction with His6-tagged HIV-1 IN, Ku70 protein, and Ku70/Ku80 heterodimer immobilized on the HTE sensor chip. The mean value of two independent experiments for each time point is shown. (**E**) The effect of s8, s9, and s17 on cell viability. 293T cells were treated with indicated concentrations of the compounds for 24 h. MTT assay was performed to measure cell viability. Mean values ± SD (*n* = 3) are shown. (**F**) The effect of s8, s9, and s17 on 293T cells transduction with HIV_wt. 293T were transduced with firefly luciferase coding HIV-based vector at MOI 0.1. Simultaneously, the cells were treated with tested compounds. Luciferase expression was assayed 24 h after transduction. Data were normalized to the luciferase expression level in DMSO-treated samples. Mean values ± SD (*n* = 3) are shown. (**G**) Cell viability (24 h) of 293T, Jurkat, and CEM in the presence of s17 measured with MTT assay. Mean values ± SD (*n* = 3) are shown. (**H**) The effect of s17 on 293T, Jurkat, and CEM transduction with HIV_wt vector. Data were normalized to the luciferase expression level in DMSO-treated samples. Mean values ± SD (*n* = 3) are shown.

All compounds were tested using an in vitro system based on genetically encoded fluorescent tags linked to both IN and Ku70, which are described in [25]. Among them, 11 compounds inhibited the formation of the IN/Ku70 complex by ~30%, 2 compounds by ~50%, 1 compound by 70%, and 2 compounds by 90% at 100 μM (Figure 1A, Table S2). Further studies were continued with the three most active compounds that inhibited the IN/Ku70 complex formation by 70% or more at 100 μM; their structures are shown in Figure 1B. Compound 8009-7811 (hereinafter s8) had IC50 = 70 ± 15 μM, 8009-9264 (hereinafter s9) 40 ± 8 μM, and only 8018-2468 (hereinafter s17) inhibited the complex formation with IC50 = 13 ± 2 μM (Figure 1C). 

To confirm that this inhibition of the protein–protein interaction was due to the binding of the tested compounds to Ku70, which was used as a receptor for molecular docking, but not to IN, the interaction of Ku70, Ku70/Ku80 heterodimer and IN with the most active compound s17 was analyzed using the surface plasmon resonance (SPR) assay. The binding of s17 to Ku70 and the Ku70/Ku80 heterodimer and the slow dissociation kinetics of the resulting complexes were detected (Figure 1D). In the case of IN immobilized on the HTE sensor chip, the addition of s17 also increased the SPR signal, but to a much lower level. After replacing the solution containing s17 with an empty buffer, the IN/s17 complexes immediately dissociated (Figure 1D). Therefore, s17 inhibited the formation of the IN/Ku70 complex predominantly through the binding to Ku70, but not to IN. 

### 2.2. s17 Inhibited the Early Replication Events in the HIV-1 Life Cycle

To assess the effect of the selected compounds on the early events of HIV-1 replication ex vivo, we used 293T cells and a VSV-G-pseudotyped, replication-incompetent HIV-based vector, encoding firefly luciferase under the control of CMV promoter (hereinafter HIV_wt). First, the cytotoxicity of the compounds was measured using MTT-test. Compounds s9 and s17 did not significantly affect cell proliferation and did not cause cell death up to 50 μM, whereas s8 did. At the maximal tested concentration (50 μM), s8 reduced cell viability to 40–45% (Figure 1E). Second, the effect of the compounds on the efficiency of 239T cell transduction with HIV_wt was measured. The inhibition of cell transduction was found only for s17, but not for s8 or s9. EC50 for s17 in this cell line was 12 ± 3 μM (Figure 1F). Importantly, resynthesized s17 (synthesis and characterization of compound described in Section S1) inhibited cell transduction using HIV_wt at the same efficiency (Section S2, Figure S4). Comparable EC50 and cell survival profiles were obtained for s17 in Jurkat (EC50 = 17 ± 2 μM) and CEM (EC50 = 10 ± 1.5 μM) cells (Figure 1G,H). 

The lentiviral vector used here adequately reproduces only the early stages of HIV-1 replication, including reverse transcription, integration, and post-integration repair. However, if the test compound affects the expression of a reporter gene encoded by the vector at the transcriptional level, the transduction efficiency will also be reduced, although this effect is not directly related to HIV replication, since this gene is under the control of the CMV promoter. To rule out a possible effect of s17 on the reporter gene expression from the CMV promoter, 293T cells with stably integrated vector cDNA were prepared. Using this cell line, we demonstrated that s17 does not alter the expression of firefly luciferase in the case of integrated vector cDNA (Section S2, Figure S5).

**Figure 2:**
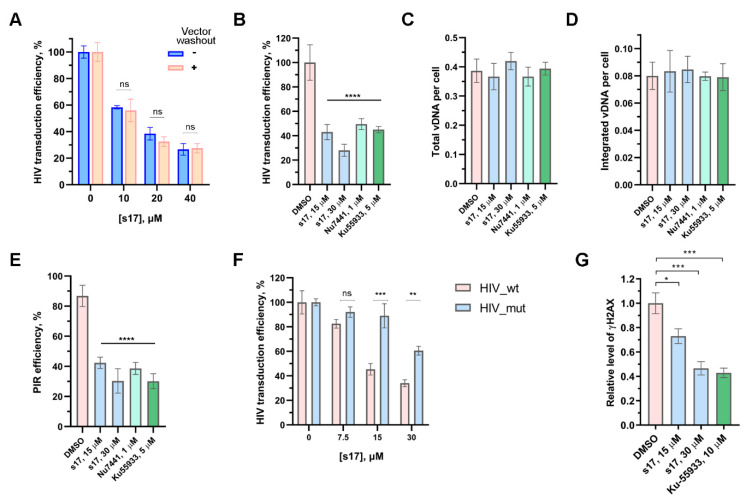
s17 inhibits HIV replication at the post-integration DNA repair step. (**A**) HIV vector washout experiment demonstrating no influence of s17 on the vector entry. s17 was added to 293T cells at indicated concentrations simultaneously with the HIV_wt vector or after vector washing out. At 24 h post-transduction, the luciferase expression level was measured. Data were normalized to DMSO-treated samples independently for both types of experiments. (**B**–**E**) The effect of s17, Nu7441 and Ku-55933 on the cell transduction efficiency with HIV_wt (**B**), total (**C**) or integrated (**D**) HIV cDNA level and post-integration DNA repair efficiency (**E**). (**F**) Transduction efficiency of 293T cells with HIV_wt or HIV_mut carrying IN_E212A/L213A variant unable to bind Ku70 in yjr presence of an increasing concentration of s17. (**G**) Influence of s17 and Ku-55933 on γH2AX accumulation measured 12 h post-transduction with HIV_wt. Data were normalized to DMSO-treated samples. Mean values ± SD (*n* = 3) are shown in all graphs. Significance was determined with one-way ANOVA with Bonferroni correction, * = *adj.p* < 0.05, ** = *adj.p* < 0.01, *** = *adj.p* < 0.001, **** = *adj.p* < 0.0001, ns—non-significant.

To be completely sure that s17 precisely inhibits the early stages of HIV-1 replication, it was necessary to exclude its effect on the entry of the VSV-G-pseudotyped pseudovirus into cells. For this purpose, 293T cells were transduced with HIV_wt. Two hours post-transduction, the cells were washed with PBS and the fresh medium without the vector being added. Three hours post-transduction, s17 was added to the transduced cells. In parallel, 293T cells were transduced with HIV_wt and immediately treated with s17. A comparison of the results of both experiments demonstrated that s17 was equally efficient in inhibiting cell transduction with HIV_wt both in the case of adding the inhibitor simultaneously with the vector and in the case of washing off the vector with a subsequent addition of the inhibitor (Figure 2A). Therefore, s17 did not influence the vector entry but acted on subsequent steps. 

### 2.3. s17 Inhibited HIV-1 Post-Integration DNA Repair Ex Vivo

To characterize which step of the HIV life cycle was affected by s17, 293T cells were transduced with HIV_wt in the presence of increasing concentrations of the tested compound, and the level of total and integrated viral DNA, as well as PIR efficiency, were measured using previously described real-time PCR techniques [26,27]. We demonstrated that the decreased transduction efficiency in the presence of s17 (Figure 2B) results from the inhibition of PIR (Figure 2E), but not reverse transcription (Figure 2C) or integration (Figure 2D). The same type of HIV replication inhibition was shown in cells treated with Nu7441 and Ku-55933 (Figure 2B–E), well-known inhibitors of DNA-PKcs and ATM kinases, respectively, acting downstream of the IN/Ku70 complex during PIR [28].

Previously, we have demonstrated that the HIV-based vector containing a mutant variant of IN (IN_E212A/L213A) that is unable to bind Ku70 (HIV_mut) transduces different cell lines 5–7 times less efficiently than HIV_wt due to impaired PIR. Moreover, the HIV_mut vector was less sensitive to DNA-PKcs and ATM inhibitors [28]. It is clear that when the interaction of IN and Ku70 is impaired, downstream kinases are not activated, and the addition of their inhibitors does not affect the behavior of the lentiviral vector. In the case of s17, we observed a similar effect. Namely, s17 efficiently inhibited HIV_wt, but not HIV_mut (Figure 2F). This result additionally confirms the effect of s17 on the formation of the IN/Ku70 complex. 

Thus, we are the first to have demonstrated that the disruption of the IN/Ku70 complex formation by a low-molecular-weight compound inhibits HIV-1 replication at the post-integration repair step.

### 2.4. s17 Impaired γH2AX Accumulation

ATM and DNA-PKcs, activated by damaged DNA, phosphorylate many target proteins that participate in the DNA damage response and repair [22,29,30]. Among them, the reparative histone H2AX occupies a special place. This minor histone H2A variant is rapidly phosphorylated at Ser139 by the kinases indicated above to produce γH2AX and serves as a platform for the recruitment of further DNA repair factors needed to repair the damage [31,32]. As it was previously shown, γH2AX accumulates in cells transduced by HIV- or ASV-based lentiviral vectors [28,33]. This process strongly depends, firstly, on the ability of IN to bind Ku70, and secondly, on the kinase activity of both DNA-PKcs and ATM [28]. Here, we verified whether s17 could prevent γH2AX accumulation in cells transduced with HIV_wt. For this purpose, 293T cells were transduced with HIV_wt in the presence of s17 or Ku-55933. The γH2AX level was measured 12 h post-transduction. Ku-55933, as previously shown, disrupted γH2AX accumulation (Figure 2G). In cells treated with s17, a decrease in the level of γH2AX was also observed (Figure 2G). Therefore, the low-molecular-weight inhibitor preventing the formation of the IN/Ku70 complex blocked the downstream activation of DNA-PKcs and ATM during PIR.

### 2.5. s17 Did Not Affect the NHEJ Pathway of the DNA Double-Strand Break Repair

The Ku heterodimer is best characterized for its central role as an initial DNA-end-binding factor in the cNHEJ pathway, the main DSB repair pathway in mammals [34]. The central ring of the Ku heterodimer binds DNA ends in a sequence-independent manner. The putative binding site of s17 and the DNA binding region are located in different parts of the Ku heterodimer. Nevertheless, we could not rule out that s17 binding could allosterically impair the basic function of the heterodimer. Thus, it was necessary to study the effect of s17 on the main function of Ku. 

**Figure 3:**
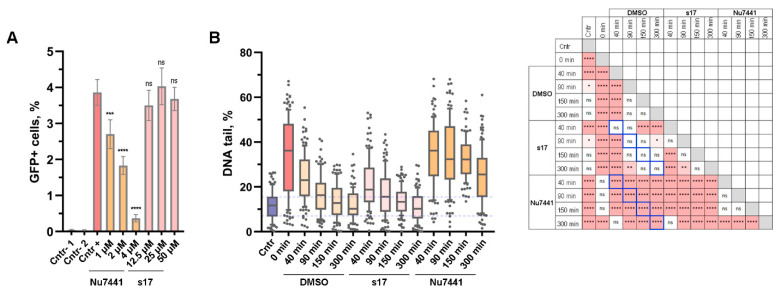
s17 did not alter the main cellular function of the Ku heterodimer, which is its role in the NHEJ pathway. (**A**) The effect of s17 and Nu7441 on overall DSB repair efficiency along the NHEJ pathway measured by the GFP-reporter system. 293T cells were co-transfected by EJ7-GFP reporter vector encoding GFP interrupted by insertion of 46 nucleotides linker, and expression plasmids for Cas9 and sgRNAs 7a and 7b. Samples noted as Cntr-1 and Cntr-2 were obtained by co-transfection of 293T cells with EJ7-GFP and 7a or 7b coding vectors, respectively. At 6 h post-transfection, the cell medium was replaced with fresh one containing tested compounds or DMSO. After 48 h, the proportion of GFP+ cells was counted using flow cytometry. (**B**) The effect of s17 (50 μM) and Nu7441 (2 μM) on DSB repair kinetics measured by the neutral comet assay. 293T cells were treated with etoposide (50 μM) for 1 h, washed three times with 1× PBS and left in fresh media containing s17, Nu7441 or DMSO for the indicated time. Analysis of fragmented DNA was performed using the neutral comet assay (100 cells per time point). Significance was determined with one-way ANOVA and Bonferroni correction, * = adj.p < 0.05, ** = adj.p < 0.01, *** = adj.p < 0.001, **** = adj.p < 0.0001, ns—non-significant. The results of multiple comparisons are presented in the table (right). Pink squares represent pairs of data that are statistically significantly different. The signs inside these squares indicate the significance level. Blue squares display the most meaningful pairs for comparison: s17 versus control (DMSO); Nu7441 versus control.

To examine the fidelity of cNHEJ in the presence of s17, we used a GFP-based reporter system in which the sequence of the GFP-coding vector is split at codon 67 by inserting a 46-nucleotide spacer that disrupts GFP expression [35]. Two other plasmids encode Cas9 and guide RNAs to this insert. When cells are co-transfected by three plasmids, Cas9 introduces two DSBs into the spacer region of the reporter. The GFP coding sequence is restored by joining the ends of the vector due to cNHEJ. Thus, the amount of GFP-positive cells correlates with the accuracy and efficiency of cNHEJ [35]. In our hands, the Nu7441 inhibitor of DNA-PKcs dose-dependently reduced the number of GFP-positive cells and therefore inhibited the cNHEJ pathway, while s17 had no effect on cNHEJ up to 50 µM (Figure 3A).

To further characterize the effect of s17 on DSB repair, the neutral comet assay was applied (Figure 3B). Etoposide was used to generate DSBs. The system was validated using the Nu7441 inhibitor of DNA-PKcs. The amount of damaged DNA decreased to the control level 150 min after replacing the medium containing etoposide with the medium containing DMSO. The presence of Nu7441 in the medium inhibited this process; the amount of damaged DNA in Nu7441-treated samples did not return to the control level even 300 min after the medium replacement. At the same time, the kinetic of damaged DNA repair in the presence of s17 was the same as in the case of the DMSO-treated samples. Therefore, s17 did not affect either the overall efficiency of DSB repair or the kinetics of this process.

### 2.6. Molecular Dynamics Model of the s17 Complex with the Ku70/Ku80 Heterodimer

**Figure 4:**
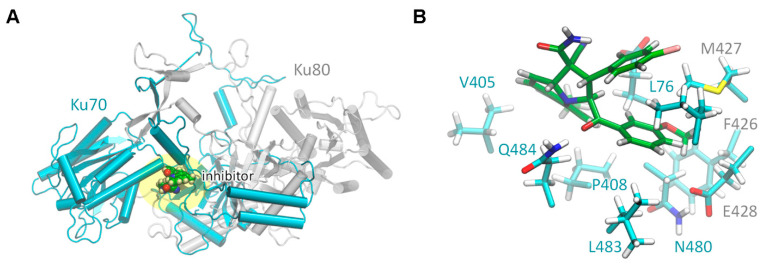
Structure of the s17/Ku70/Ku80 complex constructed with the molecular dynamics approach. (**A**) The overall view of the Ku70/Ku80 complex with the inhibitor S17. Ku70 is colored cyan, Ku80—grey. The binding area is highlighted yellow. (**B**) The composition of the Ku70/Ku80 binding site and amino acid residues interacting with the inhibitor. The carbon atoms of inhibitor are colored green; protein carbon atoms are cyan.

The model of the s17-Ku70/Ku80 complex was created to obtain an in-depth insight into the s17 binding to the Ku70/Ku80 heterodimer. This can assist in future rational modifications to increase its potency. The model of the Ku70/Ku80 heterodimer for molecular dynamics simulations was the same as in [24]. We placed the s17 compound as it was found in the molecular docking procedure and performed a molecular dynamics run for 200 ns. The s17 compound occupies the binding site in the interface between Ku70 and Ku80 monomers (Figure 4). It mainly interacts with the hydrophobic residues of the Ku70. The Ku70 L76 residue is known to be important for the Ku70 interaction with the HIV-1 IN as it forms a hydrophobic “leucine zipper” [24]. In the Ku70/Ku80 complex with s17, it is sandwiched between two six-membered rings of the s17 compound, chlorphenyl and methoxyphenyl residues (Figure 4B). This is likely to be important for the experimentally observed inhibition. The hydrophobic part of the s17 interacts with other hydrophobic fragments from P408, L483, N480, V405 and Q484 from the Ku70 as well as from F426, M427 and E428 from the Ku80 (Figure 4B). The other part of the S17 is exposed to the solution and should be more hydrophilic. The obtained complex was stable during all periods of the molecular dynamics run.

### 2.7. Chloropohenyl and Methoxyphenyl Radicals of s17 Are Essential for Its Inhibitory Activity

**Figure 5:**
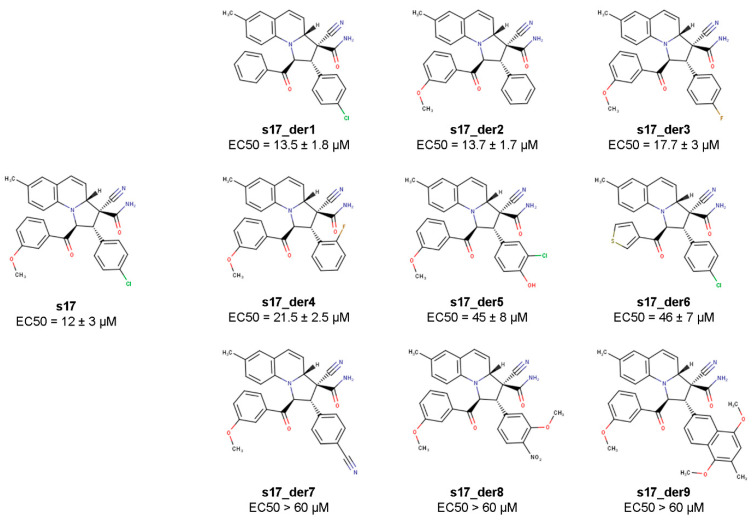
Methoxyphenyl and chlorophenyl residues of s17 are essential for HIV-1 inhibition. To measure EC50 of s17 derivatives, 293T cells were transduced with the HIV_wt vector and treated with increasing concentrations of s17 or its derivatives. At 24 h post-transduction, the luciferase expression level was measured. Normalized to DMSO-treated cells; data of three independent repeats were used to calculate EC50.

The structure of the s17-Ku70/Ku80 complex constructed with the molecular dynamics approach suggested that the chlorophenyl and methoxyphenyl substituents may play a decisive role in the activity of s17 since they bind the L76 residue of Ku70, which is involved in the IN binding. To check this hypothesis, a set of s17 derivatives with substituted chlorophenyl or methoxyphenyl radicals was synthesized (Figure 5 and Section S1, Figures S1–S3). All compounds were tested for their ability to inhibit HIV_wt transduction of 293T cells (Figure 5 and Section S2, Figure S6). As shown in Figure 5, the most dramatic increase in EC50 was detected when the chlorophenyl radical was replaced by the 1,4-dimethoxy-3-methylnaphthalen group (s17_der9) or phenyl radicals with bulky substituents (s17_der7 and s17_der8). All these substituents are larger than the chlorophenyl group, which may affect the correct positioning of the inhibitor in the binding pocket. 

A noticeable decrease in the inhibitory activity was also observed for compounds s17_der5 and s17_der6. In the first case, this can most likely be explained by the replacement of the chlorine atom by a hydrophilic hydroxyl group, which can hinder the inhibitor positioning in the hydrophobic binding pocket. The fact that replacing the methoxyphenyl with a smaller thiophene radical (s17_der6) increased the EC50 by a factor of 4.5 confirms the importance of the phenyl radical for hydrophobic interactions with the L76 residue of Ku70. It is worth noting that there were two samples among the tested compounds, s17_der1 and s17_der2, with a similar to s17 EC50. The first compound contained phenyl instead of methoxyphenyl radical, the other contained phenyl instead of chlorophenyl residue. These data indicate that the presence of these substituents in the phenyl rings is not necessary. It is interesting, however, that the replacement of chlorine by fluorine (s17_der3 and s17_der4) reduces the inhibitory effect, although only slightly. Altogether, these data confirm the important role of the two aromatic substituents for the inhibitory effect of s17.

## 3. Discussion

The current therapy used to treat the HIV infection (ART) consists of a cocktail of several compounds that act at different stages in the HIV-1 life cycle. This therapy made it possible to transfer this infection to the category of chronic diseases. However, most of the drugs used in ART inhibit viral enzymes, the genes of which accumulate mutations rather quickly due to the inaccurate work of HIV-1 reverse transcriptase. Therefore, even such combination therapy sooner or later becomes ineffective due to the emergence of drug-resistant strains of the virus. Thus, there is a clear need for the development of new drugs to be included in cART, especially those not or less subjected to the emergence of resistant strains.

One of the most promising approaches for creating such therapies is undermining an interaction of viral proteins with cellular proteins essential for viral replication. The development of viral resistance to such drugs is unlikely, since the interaction surface of the two proteins, which is the target of these drugs, is highly conservative, and any mutations in it decrease the stability of the protein–protein complex [36].

Maraviroc was the first drug of this type. It inhibits viral entry into cells by preventing the binding of the HIV gp120 protein to the CCR5 co-receptor [37]. Maraviroc has been used in cART since 2007, and since then all studies with both cART-experienced and cART-naïve patients show its efficacy and safety. It is extremely important that, so far, no HIV-1 strains resistant to maraviroc have been identified [38]. Unfortunately, maraviroc can only be prescribed to patients infected with the CCR5-tropic strain of HIV-1, which is mainly found in the early stages of the disease and in untreated patients.

In 2022, another inhibitor of protein–protein interactions, lenacapavir, was approved for medical use. Lenacapavir is the first inhibitor of viral capsid assembly/disassembly that prevents the correct interaction of the viral capsid protein (CA) with cellular proteins CPSF6 (cleavage and polyadenylation specificity factor subunit 6) and nucleoporin Nup153 [39]. In persons with HIV-1, there was no preexisting resistance to lenacapavir regardless of treatment history. In heavily treatment-experienced persons with multidrug-resistant HIV-1 and treatment-naïve persons with HIV-1, lenacapavir in combination with other antiretroviral agents led to high rates of virologic suppression and was well tolerated [40]. 

Maraviroc and lenacapavir’s clinical experience confirm that the inhibition of viral and cellular proteins’ interaction is a promising approach to the treatment of the HIV infection. Drugs that disrupt complexes of viral proteins and host cell proteins provide a high genetic barrier for the development of resistant viral strains. In this regard, the identification of these complexes would open up new possibilities for the development of new antiretroviral drugs.

IN is one of the main HIV-1 enzymes. It is responsible for the integration of viral DNA into the host cell genome [41]. Many studies have searched for IN cellular partners, and some of them are already described [1]. The best-known partner of IN is LEDGF/p75; its interaction with IN has been studied in detail, and inhibitors of this interaction, called LEDGINs or ALLINIs, have been developed [13,42]. The molecular mechanisms by which these inhibitors inhibit HIV replication are being actively studied. To date, they have been found to interfere with IN multimerization and proper virion morphogenesis [18,19].

We have previously investigated the interaction between HIV-1 IN and the cellular Ku70 protein [23]. This interaction was shown to be essential for post-integrational DNA repair [9], and the structure for the protein binding site has also been suggested [24]. 

Here, we present the results of our work on the development of inhibitors of the IN interaction with Ku70. It was important for us to show that by disrupting the binding of IN to the human Ku70 protein, it is possible to disrupt the early stages of replication, or more precisely, post-integrational repair. For this reason, we used a VSV-G pseudotyped replication-incompetent HIV-1-based vector to study the effects of our inhibitor s17 on early replication events. We can assume that s17 may also affect some later stages of HIV-1 replication, but this vector does not allow us to study later stages.

The search for inhibitors was carried out by computer docking compounds from the ChemDiv into the pocket of the Ku70 protein, which contains the amino acid residues involved in the binding to IN. Among the inhibitors found in silico, several compounds were able to disrupt the binding of Ku70 to IN, and one inhibitor, called s17, inhibited HIV-1 replication in cells. Using replication-incompetent lentiviral vectors, it was shown that s17 is able to suppress viral replication precisely at the stage of post-integration repair. It is important that at a concentration of 50 µM the s17 inhibitor is not cytotoxic, but suppresses the replication of the pseudovirus. However, we understand that the s17 inhibitor has not been optimized in terms of its solubility. We cannot evaluate the effect of higher concentrations of s17 due to its poor solubility and possible aggregation in aqueous media. 

The Ku70 protein is one of the main players in the process of DNA double-strand break repair by the NHEJ pathway in the human cell. Therefore, checking whether the s17 inhibitor impairs the NHEJ pathway of cellular DNA DSB repair was important. This was verified using a GFP-based reporter system in which the sequence of the GFP-coding vector is split by inserting a spacer that disrupts GFP expression [35]. In the case of correct functioning of the NHEJ pathway, GFP expression is restored after the spacer cleavage by the CRISPR-Cas9 system. Nu7441, the inhibitor of DNA-PKcs, dose-dependently inhibited the NHEJ pathway, while s17 had no effect on DNA DSB repair. Using the neutral comet assay, we additionally showed that s17 does not only affect the overall efficiency of DSB repair, but also the kinetics of this process.

It is clear that the inhibitory activity of the s17 inhibitor is quite average, so additional SAR studies are needed to increase the inhibitory activity and reduce the cytotoxicity of the inhibitor. To this end, we constructed a molecular dynamics model of the s17-Ku70/IN ternary complex in order to understand which structural elements of the s17 determine its activity. It has been found that chlorphenyl and methoxyphenyl radicals are important for the inhibition of the Ku binding to IN. The other part of the S17 is exposed to the solution, and possibly increasing its hydrophilicity will improve the solubility of the inhibitor and reduce its cytotoxicity. Our further research will focus on optimizing the structure of the s17 inhibitor to improve its therapeutic index.

In conclusion, it should be noted once again that we have developed a first-class inhibitor that blocks HIV-1 replication at the stage of post-integrational DNA repair. We propose to stimulate compounds that inhibit post-integration repair by disrupting the integrase binding to Ku70 KuINins.

## 4. Materials and Methods

### 4.1. Molecular Docking

System Configuration and Computational Details: This research was performed using AutoDock-GPU (v1.4.3), OpenCL and Cuda accelerated version of AutoDock4.2.6 [43] installed in macOS Catalina 10.15.7 with 16 GB RAM and AMD Radeon Pro 5300M 4 GB graphics card. The 3D visualization images were created using PyMol. Data analysis was performed using Python.

Protein Model Preparation: The X-ray crystal structure of the human Ku heterodimer (PDB ID: 1JEQ) was downloaded from Protein Data Bank [44]. All water molecules were removed, and AutoDockTools software [45] was used to prepare the required files for AutoDock-GPU by assigning hydrogen polarities, calculating Gasteiger charges to protein structures and converting protein structures from the PDB file format to PDBQT format. Energy grid maps were calculated using AutoGrid program [45]. A grid size was set to 82 × 72 × 76 (x, y and z) points with a spacing of 0.292 Å. The grid center was designated at x, y and z dimensions of 41.744, 8.608 and 128.236, respectively.

Ligand Preparation: The virtual compound library from ChemDiv was downloaded from the company website in SMILES format. After the initial filtering using RDKit, the library was converted to PDB format using the open source program for preparing small-molecule libraries Gypsum-DL [45]. All of the hydrogen atoms were added to the ligand molecules. Ligand ionization states were generated at pH 7.2.

Docking parameters: Chosen compounds were subjected to docking with the following parameters: 100 LGA runs, 2,500,000 score evaluations (max.) per LGA run, 42,000 generations (max.) per LGA, ADADELTA local search method, 300 local search iterations (max.), 150 population size, 2% mutation rate, 80% crossover rate, 80% local search rate and 60% tournament (selection) rate.

### 4.2. Molecular Dynamics Simulations

Full-atom molecular model of the Ku70/Ku80 heterodimer was constructed as in ref. [24] and by taking PDB ID 1JEQ [44] as a source of coordinates of heavy atoms. The CGenFF [46] parameters were utilized for s17. CHARMM36 force filed parameters [47] were utilized for protein and TIP3P [48] for water molecules. All MD simulations were performed in the NPT ensemble at T = 300 K and p = 1 atm. Langevin dynamics were used for the temperature control. Constant pressure was achieved using the modified Nosé–Hoover method, in which, Langevin dynamics were used to control fluctuations in the barostat [49,50]. A total of 10,000 steps of energy minimization and 30 ns molecular dynamic simulation were performed to equilibrate the system. The 200 ns production run was performed to analyze the stability of the s17-Ku70/Ku80 complex.

### 4.3. Recombinant Proteins Expression and Purification 

GST-mCer-IN and His6-Ku70-tRFP were expressed and purified as previously described [25]. Ku70/Ku80 heterodimer was expressed and purified as previously described [51].

### 4.4. Protein Binding Assay for Search of Inhibitors 

To investigate the influence of low-molecular-weight inhibitors on the His6-Ku70-tRFP/GST-mCer-IN complex formation, indicated compounds were incubated at 100 μM or increasing concentrations (0–200 μM) with 200 nM GST-mCer-IN and 200 nM His6-Ku70-tRFP in 200 µL of buffer A (20 mM Hepes pH 7.5, 100 mM NaCl, 7.5 mM MgCl_2_, 2 mM 2-mercaptoethanol, 50 μg/mL BSA) containing 5% DMSO at room temperature for 1 h. Then, complexes His6-Ku70-tRFP/GST-mCer-IN and free His6-Ku70-tRFP were precipitated using glutathione–agarose as described above. After elution of proteins with 20 μL of 1× SDS-PAGE, the levels of His6-Ku70-tRFP and GST-mCer-IN were analyzed by standard SDS-PAGE electrophoresis with subsequent detection of fluorescence in the gel [25]. The fluorescence signals ratios (tRFP/mCer) were used as a measure of proteins’ binding efficiency. This ratio in the absence of the inhibitor was taken as 100%. 

### 4.5. SPR Assay

To analyze binding of s17 with HIV-1 IN, human Ku70 or Ku70/Ku80 heterodimer ProteOn XPR36 protein interaction array system (BioRad, Hercules, CA, USA) was used. A total of 200 nM N-terminally His6-tagged recombinant IN, Ku70 or heterodimer Ku70/Ku80 (only Ku70 contained tag) were immobilized on HTE-sensorchip (BioRad) for 7 min at 30 μL/min flow rate. To analyze binding with s17, 100 μM compound was injected to HTE-sensorchip in running buffer (20 mM Hepes pH 7.5, 100 mM NaCl, 7.5 mM MgCl_2_, 2 mM 2-mercaptoethanol, 5% DMSO) for 1 min at 100 μL/min flow rate.

### 4.6. Cell Culture and Lentiviral Vector Production

293T cells were obtained through the NIH AIDS Research and Reference Reagent Program. 293T cells were cultured in DMEM medium supplemented with 10% FBS and 100 I.U./mL penicillin/100 μg/mL streptomycin solution (all from Gibco, Waltham, MA, USA) in a 37 °C incubator with a humidified atmosphere of 5% CO_2_ in air. Jurkat and CEM (all purchased from ATCC) were cultured in RPMI medium supplemented with 10% FBS and 100 I.U./mL penicillin/100 μg/mL streptomycin solution (all from Gibco). Cell viability was measured using MTT assay. For this purpose, cells were seeded 24 h prior experiment. Tested compounds were added at indicated concentrations in 1% DMSO (final concentration at media) and cells grown for additional 24 h. At the end of the incubation period, MTT (PanEco, Moscow, Russia) was added to each well and incubation was carried out for 2 h at 37 °C. Formazan crystals were dissolved in 100% DMSO, absorbance was measured on multimode microplate reader BioTek Synergy H1 (BioTek Instruments Inc., Winooski, VT, USA) and was normalized to DMSO-treated cells. 

To generate VSV-G pseudotyped replicative incompetent HIV-based vectors HIV_wt and HIV_mut, 293T cells were co-transfected with HIV-1 packaging vector pCMVΔR8.2 (Addgene plasmid #12263) or pCMVΔR8.2_IN_E212A/L213A, vector for expression of protein G from vesicular stomatitis virus (VSV) pCMV-VSVG (Addgene plasmid #8454), and reporter plasmid pUCHR-inLuc-mR by calcium–phosphate transfection [52,53]. At 6 h post-transfection, media was replaced. At 48 and 72 h post-transfection, supernatants were harvested, and pseudoviruses were concentrated by centrifugation at 30,000× *g* for 2 h and resuspended in PBS. The MOI for HIV_wt was determined as amount of integrated DNA per cell. The level of p24 was assayed using the HIV-1 p24-antigen ELISA Kit (Vector Best, Saint Petersburg, Russia). The same amount of HIV_wt and HIV_mut measured with p24 was used in all experiments. To enhance transduction capability of pseudoviruses, Jurkat and CEM were transduced using spinoculation at room temperature and 2000× *g* for 1.5 h in the presence of 7 µg/mL of polybrene (Sigma, St. Louis, MO, USA). 

Transduced cells in presence of tested compounds in 1% DMSO were harvested at 24 h post-transduction, cell number was counted and luciferase activity in cell lysates was measured using BioTek Synergy H1 reader (BioTek Instruments Inc., USA) and luciferase assay system kit (Promega, Madison, WI, USA). Data were corrected on cell amount and normalized to 1% DMSO-treated samples.

### 4.7. Measurement of Total, Integrated Viral DNA and Effect on PIR Efficiency

To investigate efficiency of reverse transcription, integration and post-integration, DNA repair 293T cells were transduced with HIV_wt at MOI = 0.1 in presence of s17 (15 or 30 μM), Nu7441 (1 μM) or Ku-55933 (5 μM). At 24 h post-transduction, total DNA was extracted from cells using ExtractDNA Blood and Cells kit (Evrogen, Moscow, Russia). All downstream procedures were performed as previously described [26,27].

To characterize accumulation of γH2AX in presence of s17 (15 or 30 μM), 3 ×10^6^ 293T cells were transduced with HIV_wt (MOI = 50). Two hours later, media containing vector was replaced with fresh one containing DMSO, s17 or Ku-55933 at indicated concentrations. At twelve hours post-transduction, cells were harvested, washed thrice with cold 1× PBS and lysed for 30 min in 1× RIPA buffer containing 1× Halt protease and phosphatase inhibitor cocktail (ThermoScientific, Waltham, MA, USA) at 4 °C. Lysates were cleared using centrifugation for 10 min at 14,000× *g*. Total protein concentration was measured with Bradford assay and 50 µg of protein was mixed with loading buffer. For the analysis of γH2AX and actin, protein samples were separated on gradient precast gel 4–15% Mini-PROTEAN^®^ TGX™ Precast Protein Gels, 15-well plate (BioRad) and transferred to Immun-Blot PVDF membrane (BioRad) in buffer containing 50 mM Tris–HCl pH 7.5, 40 mM glycine, 20% ethanol and 0.08% SDS. The primary antibodies used were anti-γH2AX rabbit polyclonal antibody (#9718, Cell Signaling, Danvers, MA, USA) and anti-actin rabbit N-terminal antibody (A2103, Sigma). HRP-conjugated anti-rabbit (Sigma) was used as secondary antibodies. Immuno-reactive bands were detected on ChemiDoc MP system (BioRad) using Clarity Western ECL substrate (BioRad). 

### 4.8. DSB Repair Assays

GFP-based reporter assay. For the extrachromosomal DSB repair assays, 293T cells were seeded at a cell density of 0.5 × 105 cells per well of a 24-well plate. Each well was transfected with 200 ng of each sgRNA/Cas9 plasmid (7a sgRNA plasmid AddGene #113620 and 7b sgRNA plasmid #113624) and pCMV6-AC EJ7-GFP (AddGene #113617) using 3.6μL of Lipofectamine 2000 in 0.5mL of antibiotic-free media. Negative controls (Cntr-1 and Cntr-2) were transfected with pCMV6-AC EJ7-GFP and 7a or 7b coding plasmids. The culture media were replaced 6 h post-transfection and cells were treated with s17, Nu7441 in 1% DMSO or DMSO alone. Cells were analyzed with flow cytometry 2 days post-transfection using MACSQuant Analyzer (Milteyi Biotec GmbH, North Rhine-Westphalia, Germany). 

Comet assay was performed as described in [54]. To generate DSB, 293T cells were treated with 100 μM etoposide (Sigma-Aldrich) for 1 h. 

## Data Availability

The 3D model of the complex of the Ku70/Ku80 heterodimer with the s17 compound is available in Zenodo and can be accessed at https://doi.org/10.5281/zenodo.8418105 (accessed on 8 October 2023). The crystallographic data are deposited at the Cambridge Crystallographic Data Centre with CCDC 2291532.

## Supplementary Materials

The following supporting information can be downloaded at: https://www.mdpi.com/article/10.3390/ijms242417354/s1. References [55,56,57,58,59,60,61] are cited in the supplementary materials.
