# Supplementary material for: KuINins as a New Class of HIV-1 Inhibitors That Block Post-Integration DNA Repair
Source: Int J Mol Sci. 2023 Dec 11;24(24):17354. doi: 10.3390/ijms242417354 (PMC10744174; doi:10.3390/ijms242417354)
Supplement: Supplementary file 1 [file ijms-24-17354-s001.zip › sup_section S1_MG.pdf]

Supplementary Section S1. Chemical compounds synthesis and characterization

All reactions were performed in round-bottom flasks fitted with rubber septa. Reactions sensitive to air and/or moisture were performed under positive argon pressure. Air- and moisture-sensitive liquids were transferred with a syringe. Analytical thin-layer chromatography (TLC) was performed using aluminum plates pre-coated with silica gel (silica gel 60 F254, Merck or Sorbfil). TLC plates were visualized under ultraviolet light (UV) at 254 nm or stained by submersion in acidic ethanol solution of vanillin followed by brief heating (vanillin) or submersion in an aqueous solution of potassium permanganate followed by extensive washing with water ($KMnO_4$). Flash-column chromatography was carried out on silica gel (60 Å, 230–400 mesh, Sigma-Aldrich). All solvents for chromatography and extractions were technical grade and distilled prior use.

All reagents were purchased from commercial suppliers (Dalchem. Sigma-Aldrich, Acros Organics, Fluorochem) and used without further purification. The following anhydrous solvents were dried by distillation over indicated drying agents prior use: Et_2O (sodium benzophenone ketyl), CH_2Cl_2, dichloroethane (CaH_2). Toluene and Et_3N were dried over CaH_2 and stored over MS4Å.

Nuclear magnetic resonance spectra were recorded on Bruker Furier 300 and Bruker Avance II 700 instruments at the indicated temperature. Data are presented as follows: chemical shift, multiplicity (s = singlet, d = doublet, t = triplet, q = quartet, m = multiplet and/or multiple resonances), coupling constant (*J*) in Hertz, integration. Proton chemical shifts are expressed in parts per million (ppm, δ scale) and are referenced to residual protium in the NMR solvents ($C\mathit{H}Cl_3$, δ 7.26 ppm; $C\mathit{H}D_2SOCD_3$, δ 2.50 ppm,). Carbon chemical shifts are expressed in parts per million (ppm, δ scale) and are referenced to the carbon resonances of the NMR solvents ($\mathit{C}DCl_3$, δ 77.16 ppm; $(\mathit{C}D_3)_2SO$, δ 39.52 ppm).

High-resolution mass spectra were recorded on a Bruker micrOTOF-Q II mass spectrometer using electrospray ionization (ESI–TOF). Melting points were determined on Kofler melting point apparatus and uncorrected.

Chemistry experimental procedures

Synthesis of starting materials

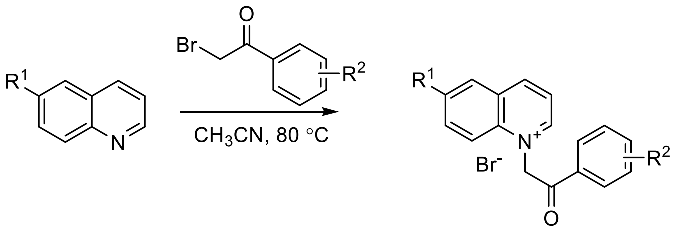

General procedure A (quinolinium salts): A stirred solution of substituted quinoline (5 mmol, 1 equiv.) and phenacyl bromide (5 mmol, 1.0 equiv.) in anhydrous CH_3CN (5 mL) was heated to 80 °C for 2-6 h. At this time some precipitate formed. The reaction mixture was allowed to cool to r.t., diluted with Et_2O (15 mL). The precipitated quinolinium salt was filtered, washed twice with Et_2O and dried on air.

6-Methyl-1-(2-oxo-2-phenylethyl)quinolin-1-ium bromide[1]

Off-white solid. Yield 1.09 g (3.2 mmol, 64%)

^{1}H (300 MHz, DMSO-d_6): δ = 9.52 (d, *J* = 5.8 Hz, 1H), 9.34 (d, *J* = 8.3 Hz, 1H), 8.37 (d, *J* = 9.1 Hz, 1H), 8.33–8.23 (m, 2H), 8.16 (d, *J* = 7.7 Hz, 2H), 8.06 (dd, *J* = 9.2, 2.1 Hz, 1H), 7.82 (t, *J* = 7.3 Hz, 1H), 7.69 (t, *J* = 7.5 Hz, 2H), 7.08 (s, 2H), 2.60 (s, 3H).

^{13}C{^{1}H} NMR (75 MHz, DMSO-d_6): δ = 190.8, 149.8, 147.5, 140.3, 137.8, 137.2, 134.8, 133.6, 129.5, 129.0 (2×), 128.9, 128.7 (2×), 122.1, 118.9, 63.3, 20.8.

1-(2-(3-Methoxyphenyl)-2-oxoethyl)-6-methylquinolin-1-ium bromide

Beige solid. Yield 1.45 g (3.9 mmol, 78%)

^{1}H NMR (300 MHz, DMSO-d_6): δ = 9.41 (dd, *J* = 5.9, 1.4 Hz, 1H), 9.32 (d, *J* = 8.3 Hz, 1H), 8.46–8.20 (m, 3H), 8.07 (dd, *J* = 9.1, 2.0 Hz, 1H), 7.75 (dt, *J* = 7.6, 1.3 Hz, 1H), 7.69–7.52 (m, 2H), 7.47–7.28 (m, 1H), 6.98 (s, 2H), 3.88 (s, 3H), 2.62 (s, 3H).

^{13}C{^{1}H} NMR (75 MHz, DMSO-d_6): δ = 190.7, 159.5, 149.7, 147.5, 140.3, 137.8, 137.1, 134.9, 130.2, 129.5, 128.9, 122.1, 121.1, 120.7, 118.9, 113.3, 63.4, 55.7, 20.8.

HRMS (ESI) m/z: $[M]^+$ Calcd for $C_{19}H_{18}NO_2$ 292.1332; Found 292.1335.

1-(2-Oxo-2-(thiophen-3-yl)ethyl)quinolin-1-ium bromide

Off-white solid. Yield 1.37 g (4.0 mmol, 79%)

^{1}H NMR (300 MHz, DMSO-d_6): δ = 9.53 (d, *J* = 5.8 Hz, 1H), 9.33 (d, *J* = 8.4 Hz, 1H), 8.94 (dd, *J* = 2.7, 1.3 Hz, 1H), 8.39–8.22 (m, 3H), 8.06 (dd, *J* = 9.2, 2.0 Hz, 1H), 7.81 (dd, *J* = 5.1, 2.8 Hz, 1H), 7.65 (dd, *J* = 5.1, 1.3 Hz, 1H), 6.96 (s, 2H), 2.59 (s, 3H).

^{13}C{^{1}H} NMR (75 MHz, DMSO-d_6): δ = 185.0, 149.8, 147.6, 140.4, 138.0, 137.9, 137.1, 136.3, 129.5, 128.9, 128.3, 126.5, 122.1, 118.8, 63.2, 20.8.

HRMS (ESI) m/z: $[M]^+$ Calcd for $C_{16}H_{14}NOS$ 268.0791; Found 268.0799.

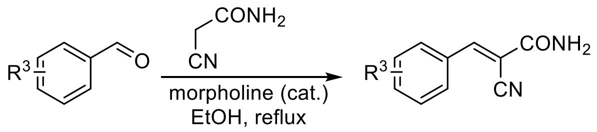

General procedure B (cyanoacrylamides): Corresponding aldehyde (10 mmol, 1 equiv.), 2-cyanoacetamide (924 mg, 11 mmol, 1.1 equiv.) and mopholine (86 µL, 87 mg, 1 mmol, 10 mol-%) in ethanol (30 mL) were heated to reflux for 2-10 h. Then the reaction mixture was allowed to cool to r.t., and afterwards put in a freezer overnight. Formed precipitate was filtered, washed with cold ethanol twice and air-dried to give target cyanoacrylamide (40-80% yield).

(*E*)-3-(4-chlorophenyl)-2-cyanoacrylamide[3]

Yellowish solid. Yield 1.53 g (7.4 mmol, 74%)

^{1}H NMR (300 MHz, DMSO-d_6): δ = 8.18 (s, 1H), 8.00–7.87 (m, 3H), 7.81 (br s, 1H), 7.64 (d, *J* = 8.9 Hz, 2H).

^{13}C{^{1}H} NMR (75 MHz, DMSO-d_6): δ = 162.5, 149.3, 136.9, 131.7 (2×), 130.8, 129.4 (2×), 116.3, 107.3.

(*E*)-2-cyano-3-phenylacrylamide[3]

White solid. Yield 1.38 g (8.0 mmol, 80%)

^{1}H NMR (300 MHz, $CDCl_3$): δ = 8.35 (s, 1H), 7.96 (d, *J* = 7.8 Hz, 2H), 7.62–7.44 (m, 3H), 6.39 (br s, 1H), 6.22 (br s, 1H).

^{13}C{^{1}H} NMR (75 MHz, CDCl3): δ = 162.4, 154.1, 133.2, 131.7, 130.9 (2×), 129.4 (2×), 117.1, 103.3.

(*E*)-2-cyano-3-(4-fluorophenyl)acrylamide[3]

Yellowish solid. Yield 1.45 g (7.6 mmol, 76%)

^{1}H NMR (300 MHz, $CDCl_3$, 310 K): δ = 8.29 (s, 1H), 7.98 (dd, *J* = 8.6, 5.5 Hz, 2H), 7.18 (t, *J* = 8.6 Hz, 2H), 6.42 (br s, 2H).

^{13}C{^{1}H} NMR (75 MHz, $CDCl_3$): δ = 165.4 (d, *J* = 257.1 Hz), 162.2, 152.6, 133.40 (d, *J* = 9.2 Hz, 2×), 128.1 (d, *J* = 3.4 Hz), 117.0, 116.82 (d, *J* = 22.1 Hz, 2×), 102.87 (d, *J* = 2.4 Hz).

(*E*)-2-cyano-3-(2-fluorophenyl)acrylamide[3]

White solid. Yield 1.17 g (6.1 mmol, 61%)

^{1}H NMR (300 MHz, $CDCl_3$): δ = 8.59 (s, 1H), 8.23 (td, *J* = 7.6, 1.7 Hz, 1H), 7.51 (dddd, *J* = 8.8, 7.2, 5.3, 1.7 Hz, 1H), 7.26 (td, *J* = 7.6, 1.2 Hz, 1H), 7.15 (ddd, *J* = 9.1, 8.4, 1.2 Hz, 1H), 6.42 (br s, 2H).

^{13}C{^{1}H} NMR (75 MHz, $CDCl_3$): δ: 161.9^0, 161.8^9 (d, *J* = 257.4 Hz), 145.6 (d, *J* = 7.3 Hz), 135.0 (d, *J* = 9.1 Hz), 129.0, 125.0 (d, *J* = 3.8 Hz), 120.2 (d, *J* = 11.0 Hz), 116.7, 116.4 (d, *J* = 21.7 Hz), 105.4.

(*E*)-2-cyano-3-(4-cyanophenyl)acrylamide[3]

Greyish solid. Yield 1.47 g (7.5 mmol, 75%)

^{1}H NMR (300 MHz, DMSO-d_6): δ = 8.24 (s, 1H), 8.10–7.96 (m, 5H), 7.88 (br s, 1H).

$^{13}C\{^1H\}$ NMR (75 MHz, DMSO-d_6): δ = 162.1, 148.8, 136.3, 133.0 (2×), 130.3 (2×), 118.2, 115.8, 113.8, 110.0.

(*E*)-2-cyano-3-(3-methoxy-4-nitrophenyl)acrylamide

Yellow solid. Performed on 2.0 mmol. Yield 301 mg (1.2 mmol, 61%)

^1H NMR (300 MHz, DMSO-d_6): δ = 8.24 (s, 1H), 8.18–7.73 (m, 4H), 7.62 (d, *J* = 8.3 Hz, 1H), 3.95 (s, 3H).

$^{13}C\{^1H\}$ NMR (75 MHz, DMSO-d_6): δ = 162.0, 151.8, 148.8, 140.4, 137.4, 125.6, 121.4, 115.9, 115.7, 110.2, 56.8.

HRMS (ESI) m/z: $[M+H]^+$ Calcd for $C_{11}H_{10}N_3O_4$ 248.0666; Found 248.0654.

(*E*)-2-cyano-3-(1,4-dimethoxy-3-methylnaphthalen-7-yl)acrylamide

Yellow solid. Performed on 2.0 mmol. Yield 396 mg (1.3 mmol, 67%)

^1H NMR (300 MHz, DMSO-d_6): δ = 8.62 (d, *J* = 1.8 Hz, 1H), 8.30 (s, 1H), 8.12 (dd, *J* = 8.9, 1.8 Hz, 1H), 8.03 (s, 1H), 7.92 (s, 1H), 7.75 (s, 1H), 6.91 (s, 1H), 3.96 (s, 3H), 3.79 (s, 3H), 2.42 (s, 3H).

$^{13}C\{^1H\}$ NMR (75 MHz, DMSO-d_6): δ = 163.1, 151.7, 150.3, 146.5, 129.8, 129.2, 128.1, 126.8, 125.5, 123.9, 122.5, 116.7, 108.7, 105.7, 61.1, 55.9, 16.3.

HRMS (ESI) m/z: $[M+H]^+$ Calcd for $C_{17}H_{17}N_2O_3$ 297.1234; Found 297.1234.

(*E*)-2-cyano-3-(3-chloro-4-hydroxyphenyl)acrylamide

Yellow solid. Performed on 2.0 mmol. Yield 178 mg (0.8 mmol, 40%)

^1H NMR (300 MHz, DMSO-d_6): δ = 11.26 (br s, 1H), 8.13–7.88 (m, 2H), 7.88–7.51 (m, 3H), 7.10 (d, *J* = 8.6 Hz, 1H).

$^{13}C\{^1H\}$ NMR (75 MHz, DMSO-d_6): δ = 163.0, 157.1, 149.3, 132.1, 131.1, 124.1, 120.6, 117.2, 117.0, 103.3.

HRMS (ESI) m/z: $[M+H]^+$ Calcd for $C_{10}H_8{}^{35}ClN_2O_2$ 223.0269; Found 223.0277.

Cycloaddition reaction

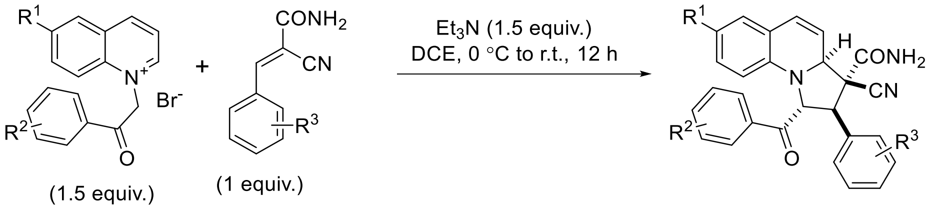

Typical procedure: Triethylamine (42 µL, 30 mg, 0.30 mmol, 1.5 equiv.) solution in dichloroethane (0.5 mL) was added dropwise during 5 min to a stirred suspension of quinolinium salt (0.30 mmol, 1.5 equiv.) and acrylamide (0.2 mmol, 1 equiv.) at 0 °C. During 1 h at 0 °C the reaction mixture turned pink and the precipitate has almost dissolved. The reaction mixture was allowed to warm to r.t. and was stirred additionally overnight. Then it was concentrated on rotary evaporator and subjected to column chromatography on silica (eluent hexane/EtOAc 2/1 → 1/1) to give desired tricyclic compound (31-87%) as yellowish powder.

***(1R*,2R*,3R*,3aS*)*-2-(4-chlorophenyl)-3-cyano-1-(3-methoxybenzoyl)-7-methyl-1,2,3,3a-tetrahydropyrrolo[1,2-a]quinoline-3-carboxamid** (named s17)

Yield 74 mg (0.15 mmol, 74%)

^{1}H NMR (300 MHz, $CDCl_3$): δ = 7.41–7.33 (m, 4H), 7.27–7.17 (m, 3H), 7.07 (dt, *J* = 7.4, 2.1 Hz, 1H), 6.81–6.71 (m, 2H), 6.60 (dd, *J* = 10.6, 2.5 Hz, 1H), 6.11 (s, 1H), 5.89 (d, *J* = 7.8 Hz, 1H), 5.69–5.51 (m, 3H), 5.38 (d, *J* = 8.1 Hz, 1H), 4.12 (d, *J* = 8.1 Hz, 1H), 3.69 (s, 3H), 2.17 (s, 3H).

^{13}C{^{1}H} NMR (75 MHz, $CDCl_3$): δ = 197.6, 164.4, 160.0, 139.7, 135.9, 135.3, 133.2, 130.4 (2×), 130.2, 129.9^{3}, 129.8^{7}, 129.6, 128.9, 127.9 (2×), 121.4, 121.3, 119.9, 117.7, 117.2, 112.5, 109.8, 69.7, 67.1, 63.7, 55.4, 54.5, 20.4.

HRMS (ESI) m/z: $[M+H]^+$ Calcd for $C_{29}H_{25}{}^{35}ClN_3O_3$ 498.1579; Found 498.1580.

***(1R*,2R*,3R*,3aS*)*-1-benzoyl-2-(4-chlorophenyl)-3-cyano-7-methyl-1,2,3,3a-tetrahydropyrrolo[1,2-a]quinoline-3-carboxamide** (named s17_der1)

Yield 74 mg (0.16 mmol, 76%)

^{1}H NMR (300 MHz, $CDCl_3$): δ = 7.71 (d, *J* = 7.0 Hz, 2H), 7.53 (t, *J* = 7.5 Hz, 1H), 7.40–7.27 (m, 6H), 6.81–6.67 (m, 2H), 6.59 (dd, *J* = 10.7, 2.4 Hz, 1H), 6.14 (br s, 1H), 5.89 (d, *J* = 7.9 Hz, 1H), 5.76 (br s, 1H), 5.67–5.54 (m, 2H), 5.40 (d, *J* = 8.2 Hz, 1H), 4.10 (d, *J* = 8.2 Hz, 1H), 2.17 (s, 3H).

^{1}H NMR (300 MHz, DMSO-d_6): δ = 7.93 (s, 1H), 7.86–7.73 (m, 3H), 7.57 (t, *J* = 7.4 Hz, 1H), 7.49 (d, *J* = 8.6 Hz, 2H), 7.44–7.32 (m, 4H), 6.81 (s, 1H), 6.76 (dd, *J* = 8.1, 2.0 Hz, 1H), 6.62 (dd, *J* = 10.2, 2.0 Hz, 1H), 5.96 (d, *J* = 8.1 Hz, 1H), 5.85 (d, *J* = 8.0 Hz, 1H), 5.67 (dd, *J* = 10.2, 2.3 Hz, 1H), 5.36 (t, *J* = 2.2 Hz, 1H), 4.06 (d, *J* = 8.0 Hz, 1H), 2.12 (s, 3H).

^{13}C{^{1}H} NMR (75 MHz, DMSO-d_6): δ = 198.3, 163.7, 139.5, 134.4, 133.6, 133.3, 130.8, 130.5, 129.8, 129.2, 128.7 (2×), 128.6 (2×), 128.0, 127.2, 126.1 (2×), 119.5 (2×), 118.0, 116.9, 110.0, 68.4, 64.6, 63.4, 53.8, 19.9.

HRMS (ESI) m/z: $[M+H]^+$ Calcd for $C_{28}H_{23}{}^{35}ClN_3O_2$ 468.1473; Found 468.1479.

_(1R*,2R*,3R*,3aS*)_-3-cyano-1-(3-methoxybenzoyl)-7-methyl-2-phenyl-1,2,3,3a-tetrahydropyrrolo[1,2-a]quinoline-3-carboxamide (named s17_der2)

Yield 52 mg (0.11 mmol, 56%)

^{1}H NMR (300 MHz, DMSO-d_6): δ = 7.90 (br s, 1H), 7.74 (br s, 1H), 7.50–7.31 (m, 6H), 7.27 (t, *J* = 7.8 Hz, 1H), 7.22 (s, 1H), 7.11 (dd, *J* = 8.4, 2.5 Hz, 1H), 6.82 (s, 1H), 6.76 (d, *J* = 8.1 Hz, 1H), 6.61 (d, *J* = 10.1 Hz, 1H), 5.96 (d, *J* = 8.1 Hz, 1H), 5.83 (d, *J* = 7.8 Hz, 1H), 5.67 (dd, *J* = 10.1, 2.3 Hz, 1H), 5.33 (s, 1H), 4.06 (d, *J* = 7.6 Hz, 1H), 3.53 (s, 3H), 2.13 (s, 3H).

^{13}C{^{1}H} NMR (75 MHz, DMSO-d_6): δ = 197.9, 163.9, 159.2, 139.5, 135.6, 134.8, 129.8 (2×), 129.0, 128.9 (3×), 128.8, 128.7, 128.5, 128.1, 128.0, 126.0, 121.0, 120.9, 119.5, 118.1, 117.1, 112.7, 110.0, 68.5, 64.6, 63.5, 55.0, 54.1, 19.9.

HRMS (ESI) m/z: $[M+H]^+$ Calcd for $C_{29}H_{26}N_3O_3$ 464.1969; Found 464.1959.

_(1R*,2R*,3R*,3aS*)_-3-cyano-2-(4-fluorophenyl)-1-(3-methoxybenzoyl)-7-methyl-1,2,3,3a-tetrahydropyrrolo[1,2-a]quinoline-3-carboxamide (named s17_der3)

Yield 81 mg (0.17 mmol, 84%)

^{1}H NMR (300 MHz, $CDCl_3$): δ = 7.42 (dd, *J* = 8.5, 5.1 Hz, 2H), 7.25–7.15 (m, 3H), 7.11–7.01 (m, 3H), 6.79–6.68 (m, 2H), 6.58 (dd, *J* = 10.7, 2.4 Hz, 1H), 6.17 (br s, 1H), 5.95 (br s, 1H), 5.88 (d, *J* = 7.8 Hz, 1H), 5.70–5.52 (m, 2H), 5.38 (d, *J* = 8.0 Hz, 1H), 4.12 (d, *J* = 8.0 Hz, 1H), 3.68 (s, 3H), 2.17 (s, 3H).

^{13}C{^{1}H} NMR (75 MHz, $CDCl_3$): δ = 197.7, 164.5, 162.6 (d, *J* = 162.8 Hz), 160.0, 139.7, 135.9, 130.8 (d, *J* = 8.5 Hz, 2×), 130.4^0, 130.3^7, 130.1, 129.9, 128.8, 127.8, 121.3, 121.2, 119.9, 117.7, 117.3, 116.4 (d, *J* = 21.6 Hz, 2×), 116.2, 112.5, 109.8, 69.6, 67.2, 63.7, 55.4, 54.6, 20.4.

HRMS (ESI) m/z: $[M+H]^+$ Calcd for $C_{29}H_{25}FN_3O_3$ 482.1874; Found 482.1870.

_(1R*,2R*,3R*,3aS*)_-3-cyano-2-(2-fluorophenyl)-1-(3-methoxybenzoyl)-7-methyl-1,2,3,3a-tetrahydropyrrolo[1,2-a]quinoline-3-carboxamide (named s17_der4)

Yield 82 mg (0.17 mmol, 85%)

^{1}H (300 MHz, $CDCl_3$): δ = 7.67 (t, *J* = 7.1 Hz, 1H), 7.36 (qd, *J* = 7.6, 1.4 Hz, 1H), 7.29–7.20 (m, 3H), 7.17 (t, *J* = 7.8 Hz, 1H), 7.09–6.99 (m, 2H), 6.81–6.71 (m, 2H), 6.59 (d, *J* = 8.7 Hz, 1H), 6.16 (br s, 1H), 5.92 (d, *J* = 7.8 Hz, 1H), 5.81 (br s, 1H), 5.67–5.57 (m, 2H), 5.51 (d, *J* = 7.5 Hz, 1H), 4.42 (d, *J* = 7.5 Hz, 1H), 3.66 (s, 3H), 2.18 (s, 3H).

^{13}C{^{1}H} NMR (75 MHz, $CDCl_3$): δ = 197.1, 164.8, 161.4 (d, *J* = 249.3 Hz), 160.0, 139.7, 135.9, 130.9 (d, *J* = 8.5 Hz), 130.3, 129.9^3, 129.8^4, 129.8^1, 128.8, 127.7, 125.1 (d, *J* = 3.6 Hz), 122.3 (d, *J* = 13.1 Hz), 121.4, 121.3, 120.0, 117.7, 117.5, 116.4 (d, *J* = 22.4 Hz), 112.3, 109.7, 69.5, 66.3 (d, *J* = 2.0 Hz), 62.9, 55.3, 47.9 (d, *J* = 2.0 Hz), 20.4.

HRMS (ESI) m/z: $[M+H]^+$ Calcd for $C_{29}H_{25}FN_3O_3$ 482.1874; Found 482.1865.

_(1R*,2R*,3R*,3aS*)_-2-(3-chloro-4-hydroxyphenyl)-3-cyano-1-(3-methoxybenzoyl)-7-methyl-1,2,3,3a-tetrahydropyrrolo[1,2-a]quinoline-3-carboxamide (named s17_der5)

Yield 32 mg (0.06 mmol, 31%)

^{1}H NMR (300 MHz, $CDCl_3$): δ = 7.37 (d, *J* = 2.2 Hz, 1H), 7.30–7.18 (m, 5H), 7.07 (d, *J* = 8.1 Hz, 1H), 7.01 (d, *J* = 8.4 Hz, 1H), 6.78–6.70 (m, 2H), 6.58 (dd, *J* = 10.5, 2.2 Hz, 1H), 6.16 (s, 1H), 5.88 (d, *J* = 8.0 Hz, 1H), 5.74 (s, 1H), 5.65–5.56 (m, 2H), 5.33 (d, *J* = 8.1 Hz, 1H), 4.04 (d, *J* = 8.1 Hz, 1H), 3.69 (s, 3H), 2.17 (s, 3H).

^{13}C{1H} NMR (75 MHz, $CDCl_3$): δ = 197.7, 164.4, 159.9, 152.5, 139.6, 135.8, 133.1, 132.3, 130.2, 129.9, 129.5, 128.7, 127.7, 127.0, 123.8, 122.4, 121.2, 120.7, 119.8, 117.5, 117.2, 117.1, 109.7, 69.4, 67.0, 63.6, 56.9, 54.4, 20.2.

HRMS (ESI) m/z: $[M+H]^+$ Calcd for $C_{29}H_{25}{}^{35}ClN_3O_4$ 514.1528; Found 514.1513.

(1R*,2R*,3R*,3aS*)-2-(4-chlorophenyl)-3-cyano-7-methyl-1-(thiophene-3-carbonyl)-1,2,3,3a-tetrahydropyrrolo[1,2-a]quinoline-3-carboxamide (named s17_der6)

Yield 66 mg (0.14 mmol, 70%)

^{1}H NMR (300 MHz, DMSO-d_6): δ = 8.40 (dd, *J* = 2.3, 1.0 Hz, 1H), 7.92 (s, 1H), 7.78 (s, 1H), 7.60–7.50 (m, 3H), 7.46–7.37 (m, 3H), 6.81 (s, 1H), 6.77 (d, *J* = 8.4 Hz, 1H), 6.61 (dd, *J* = 10.0, 2.0 Hz, 1H), 5.94 (d, *J* = 8.0 Hz, 1H), 5.71–5.57 (m, 2H), 5.40 (t, *J* = 2.3 Hz, 1H), 4.07 (d, *J* = 8.3 Hz, 1H), 2.12 (s, 3H).

^{13}C{^{1}H} NMR (75 MHz, DMSO-d_6): δ = 193.0, 163.6, 139.6, 139.1, 135.6, 133.3, 133.2, 130.8 (2×), 129.9, 128.9, 128.6 (2×), 128.0, 127.9, 126.8, 126.2, 119.4, 117.9, 116.8, 109.9, 68.5, 66.0, 63.4, 53.7, 19.9.

HRMS (ESI) m/z: $[M+H]^+$ Calcd for $C_{26}H_{21}{}^{35}ClN_3O_2S$ 474.1038; Found 474.1029.

***(1R*,2R*,3R*,3aS*)*-3-cyano-2-(4-cyanophenyl)-1-(3-methoxybenzoyl)-7-methyl-1,2,3,3a-tetrahydropyrrolo[1,2-a]quinoline-3-carboxamide** (named s17_der7)

Yield 41 mg (0.08 mmol, 42%)

^{1}H NMR (300 MHz, $CDCl_3$): δ = 7.67 (d, *J* = 8.3 Hz, 2H), 7.56 (d, *J* = 8.3 Hz, 2H), 7.27 (s, 1H), 7.23–7.14 (m, 2H), 7.08 (ddd, *J* = 7.2, 5.0, 3.0 Hz, 1H), 6.81–6.76 (m, 2H), 6.62 (d, *J* = 9.7 Hz, 1H), 6.20 (br s, 1H), 5.93 (d, *J* = 8.1 Hz, 1H), 5.84 (br s, 1H), 5.67–5.57 (m, 2H), 5.43 (d, *J* = 8.0 Hz, 1H), 4.23 (d, *J* = 8.0 Hz, 1H), 3.72 (s, 3H), 2.18 (s, 3H).

^{13}C{^{1}H} NMR (75 MHz, $CDCl_3$): δ = 197.3, 163.9, 160.1, 140.1, 139.6, 135.8, 133.0 (2×), 130.5, 130.4, 130.0, 129.9 (2×), 129.0, 128.2, 121.1, 121.0, 119.9, 118.2, 117.4, 116.9, 113.3, 112.9, 109.8, 70.0, 67.0, 63.6, 55.5, 54.7, 20.4.

HRMS (ESI) m/z: $[M+H]^+$ Calcd for $C_{30}H_{25}N_4O_3$ 489.1921; Found 489.1934.

***(1R*,2R*,3R*,3aS*)*-3-cyano-1-(3-methoxybenzoyl)-2-(3-methoxy-4-nitro-phenyl)-7-methyl-1,2,3,3a-tetrahydropyrrolo[1,2-a]quinoline-3-carboxamide** (named s17_der8)

Yield 94 mg (0.18 mmol, 87%)

^{1}H NMR (300 MHz, $CDCl_3$): δ = 7.83 (d, *J* = 8.4 Hz, 1H), 7.32–7.17 (m, 3H), 7.17–7.02 (m, 3H), 6.86–6.73 (m, 2H), 6.61 (d, *J* = 9.9 Hz, 1H), 6.30 (s, 1H), 6.06 (s, 1H), 5.94 (d, *J* = 8.0 Hz, 1H), 5.63 (d, *J* = 9.9 Hz, 1H), 5.58 (t, *J* = 2.3 Hz, 1H), 5.43 (d, *J* = 8.0 Hz, 1H), 4.21 (d, *J* = 8.0 Hz, 1H), 3.89 (s, 3H), 3.73 (s, 3H), 2.19 (s, 3H).

$^{13}C\{^{1}H\}$ NMR (75 MHz, $CDCl_3$): δ = 197.5, 164.2, 160.2, 153.4, 141.8, 139.9, 139.6, 135.9, 130.6, 130.4, 130.2, 130.1, 129.1, 128.3, 126.6, 121.1, 120.7, 120.0, 117.6, 117.1, 114.4, 110.0^{3}, 109.9^{8}, 70.0, 67.2, 63.5, 55.6, 55.5, 55.0, 20.4.

HRMS (ESI) m/z: $[M+H]^+$ Calcd for $C_{30}H_{27}N_4O_6$ 539.1925; Found 489.1933.

***(1R*,2R*,3R*,3aS*)*-3-cyano-2-(1,4-dimethoxy-3-methylnaphthalen-7-yl)-1-(3-methoxybenzoyl)-7-methyl-1,2,3,3a-tetrahydropyrrolo[1,2-a]quinoline-3-carboxamide**
(named s17_der9)

Yield ca. 52%

The product obtained after column chromatography is contaminated with starting cyanoacrylamide (ca. 11 w-%), and is also relatively unstable. We observed poor solubility of the impurity in chloroform, and thus we prepared relatively pure sample, which was used directly for biology studies:

^{1}H NMR (300 MHz, $CDCl_3$): δ = 8.23 (d, *J* = 1.9 Hz, 1H), 8.05 (d, *J* = 8.7 Hz, 1H), 7.63 (dd, *J* = 8.6, 1.8 Hz, 1H), 7.28 (d, *J* = 7.7 Hz, 1H), 7.20 (t, *J* = 1.8 Hz, 1H), 7.09 (t, *J* = 7.9 Hz, 1H), 6.96 (dd, *J* = 8.1, 2.5 Hz, 1H), 6.80–6.71 (m, 2H), 6.66–6.51 (m, 2H) 6.05 (s, 1H), 5.91 (d, *J* = 7.8 Hz, 1H), 5.73 (t, *J* = 2.4 Hz, 1H), 5.64 (dd, *J* = 10.0, 2.4 Hz, 1H), 5.61–5.54 (m, 2H), 4.28 (d, *J* = 7.9 Hz, 1H), 3.91 (s, 3H), 3.85 (s, 3H), 3.44 (s, 3H), 2.45 (s, 3H), 2.18 (s, 3H).

However, the compound is relatively unstable. Thus, full spectral analysis was made with a sample partially contaminated with starting cyanoacrylamide:

^{1}H NMR (700 MHz, DMSO-d_6): δ = 8.04 (s, 1H), 8.00 (d, *J* = 8.7 Hz, 1H), 7.94 (s, 1H), 7.81 (d, *J* = 8.8 Hz, 1H), 7.77 (s, 1H), 7.41 (d, *J* = 7.6 Hz, 1H), 7.27–7.20 (m, 2H), 7.04 (d, *J* = 8.2 Hz, 1H), 6.87–6.77 (m, 3H), 6.65 (d, *J* = 10.1 Hz, 1H), 6.03 (d, *J* = 8.1 Hz, 1H), 5.91 (d, *J* = 7.7 Hz, 1H), 5.71 (d, *J* = 10.1 Hz, 1H), 5.40 (s, 1H), 4.26 (d, *J* = 7.7 Hz, 1H), 3.89 (s, 3H), 3.79 (s, 3H), 3.39 (s, 3H), 2.40 (s, 3H), 2.15 (s, 3H).

$^{13}C\{^{1}H\}$ NMR (176 MHz, DMSO-d_6): δ = 197.7, 164.0, 159.1, 150.6, 146.4, 139.6, 135.5, 131.0, 129.8^{3}, 129.7^{6}, 128.9, 128.0, 127.8, 126.6, 126.5, 126.1, 124.1, 122.6, 122.0, 120.9 (2×), 119.6, 118.1, 117.1, 112.7, 110.1, 107.9, 68.5, 64.9, 63.6, 60.9, 55.5, 54.8, 54.4, 19.9, 16.0.

HRMS (ESI) m/z: $[M+H]^+$ Calcd for $C_{36}H_{34}N_3O_5$ 588.2493; Found 588.2510.

Copies of NMR spectra

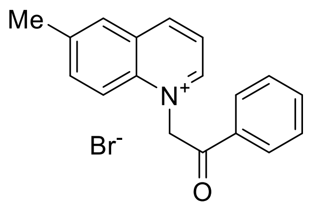

^{1}H NMR (300 MHz, DMSO-d_6)

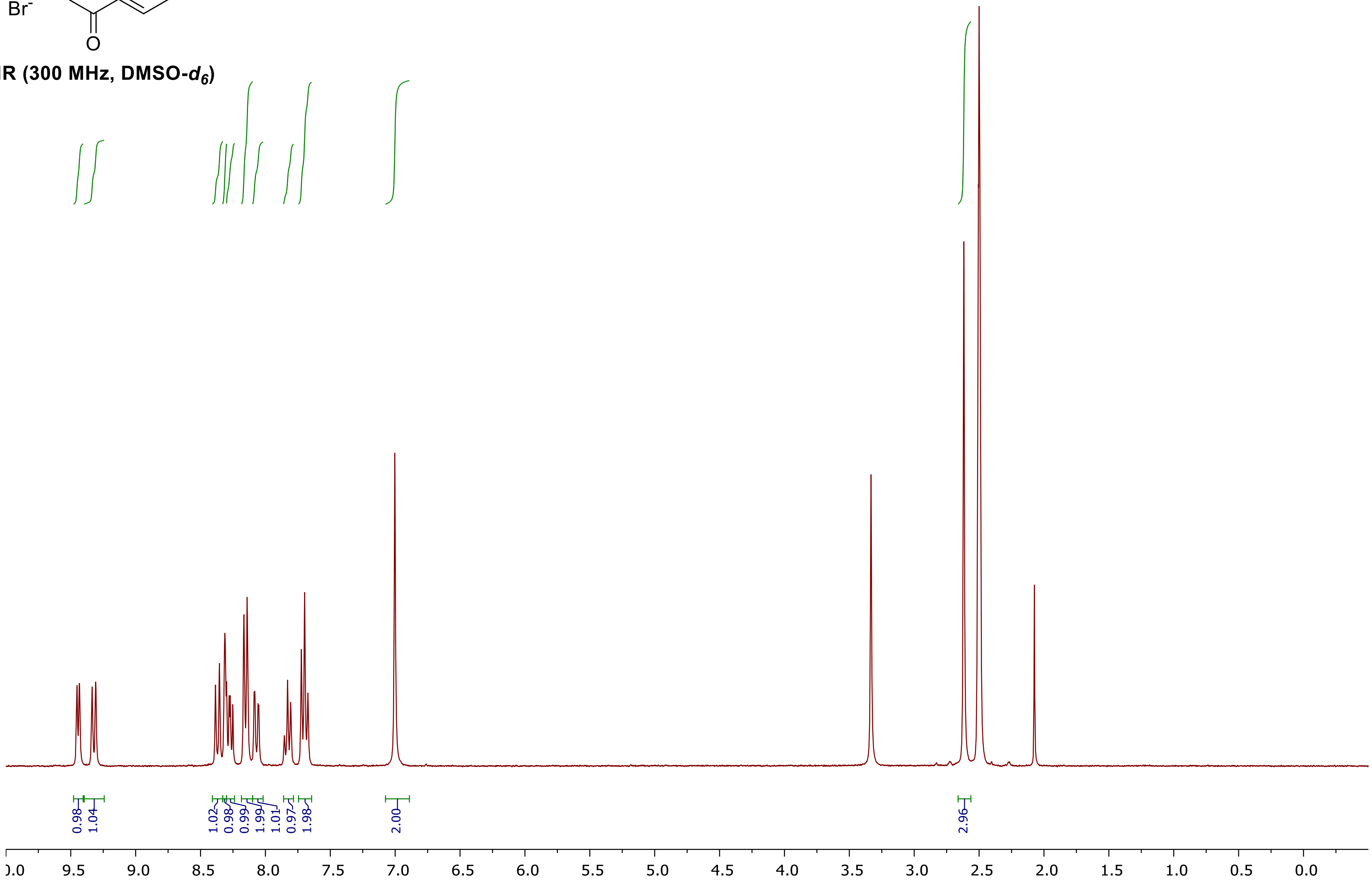

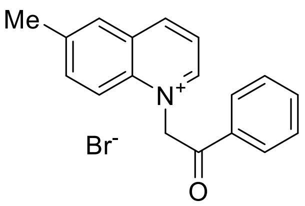

$^{13}C\{^1H\}$ NMR (75 MHz, DMSO-d_6)

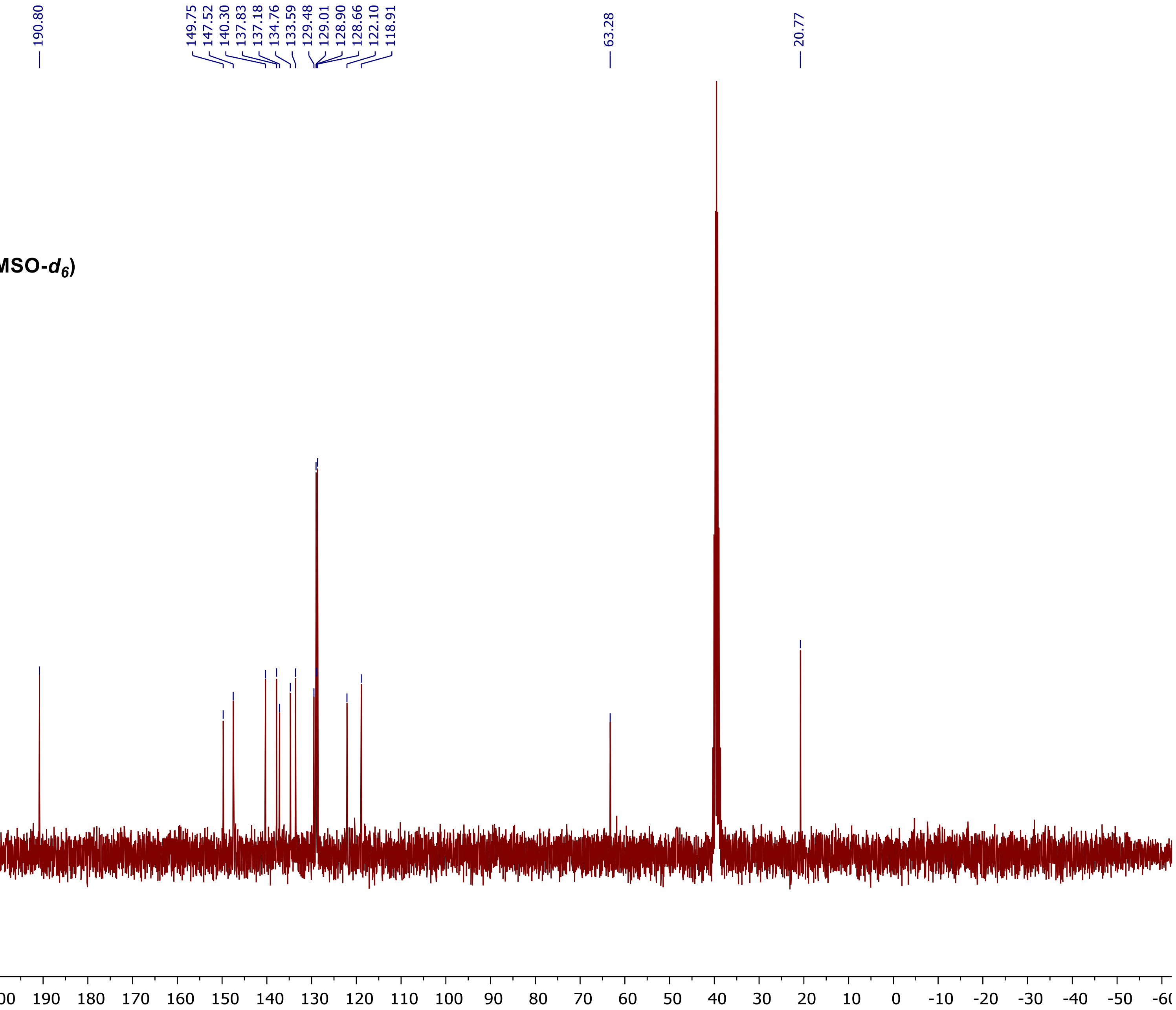

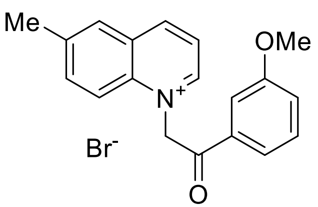

^{1}H NMR (300 MHz, DMSO-d_6)

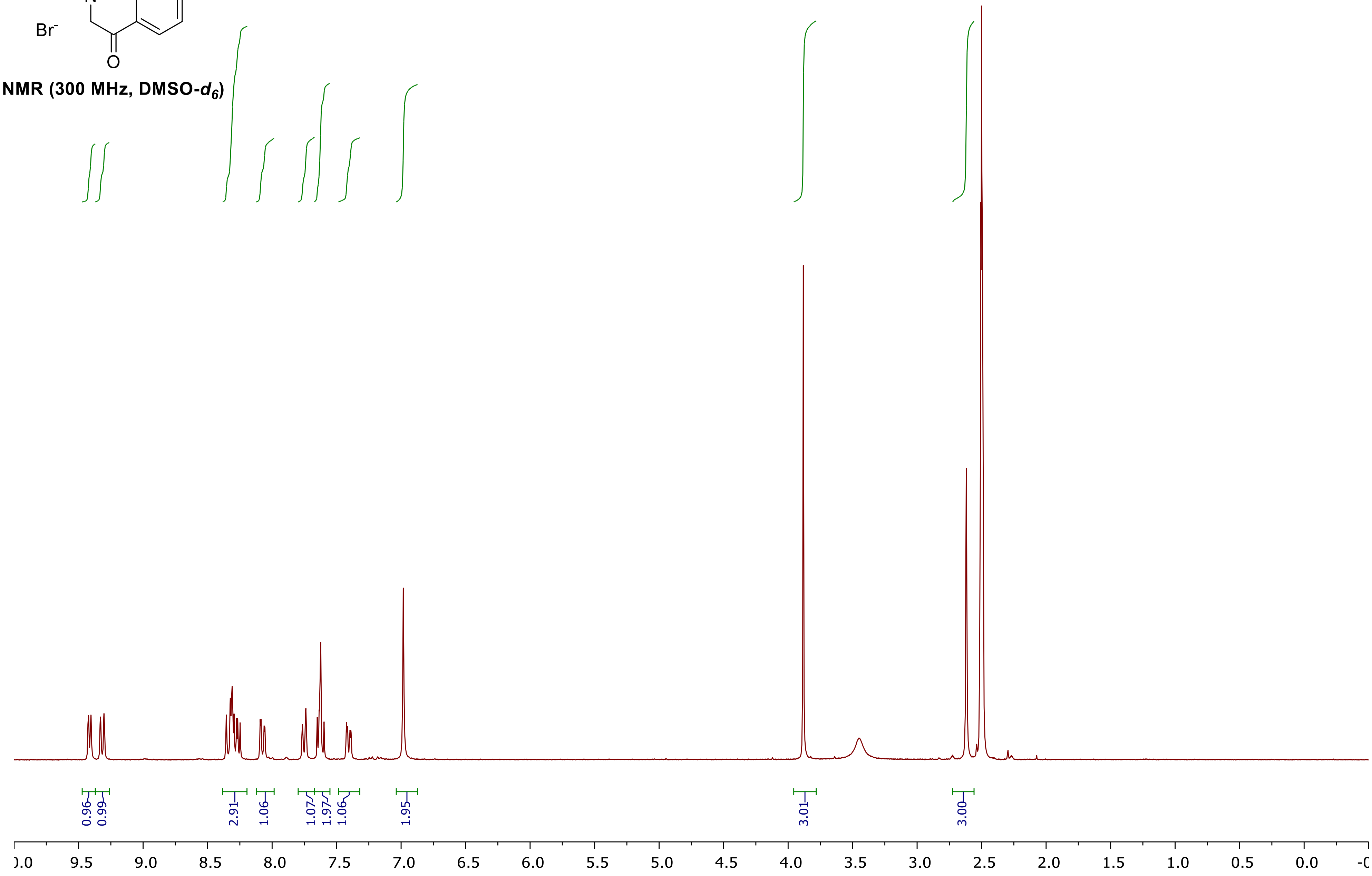

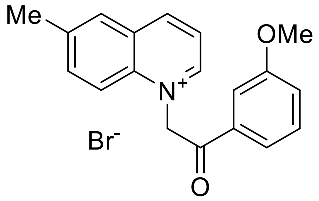

$^{13}C\{^{1}H\}$ **NMR (75 MHz, DMSO-d_6)**

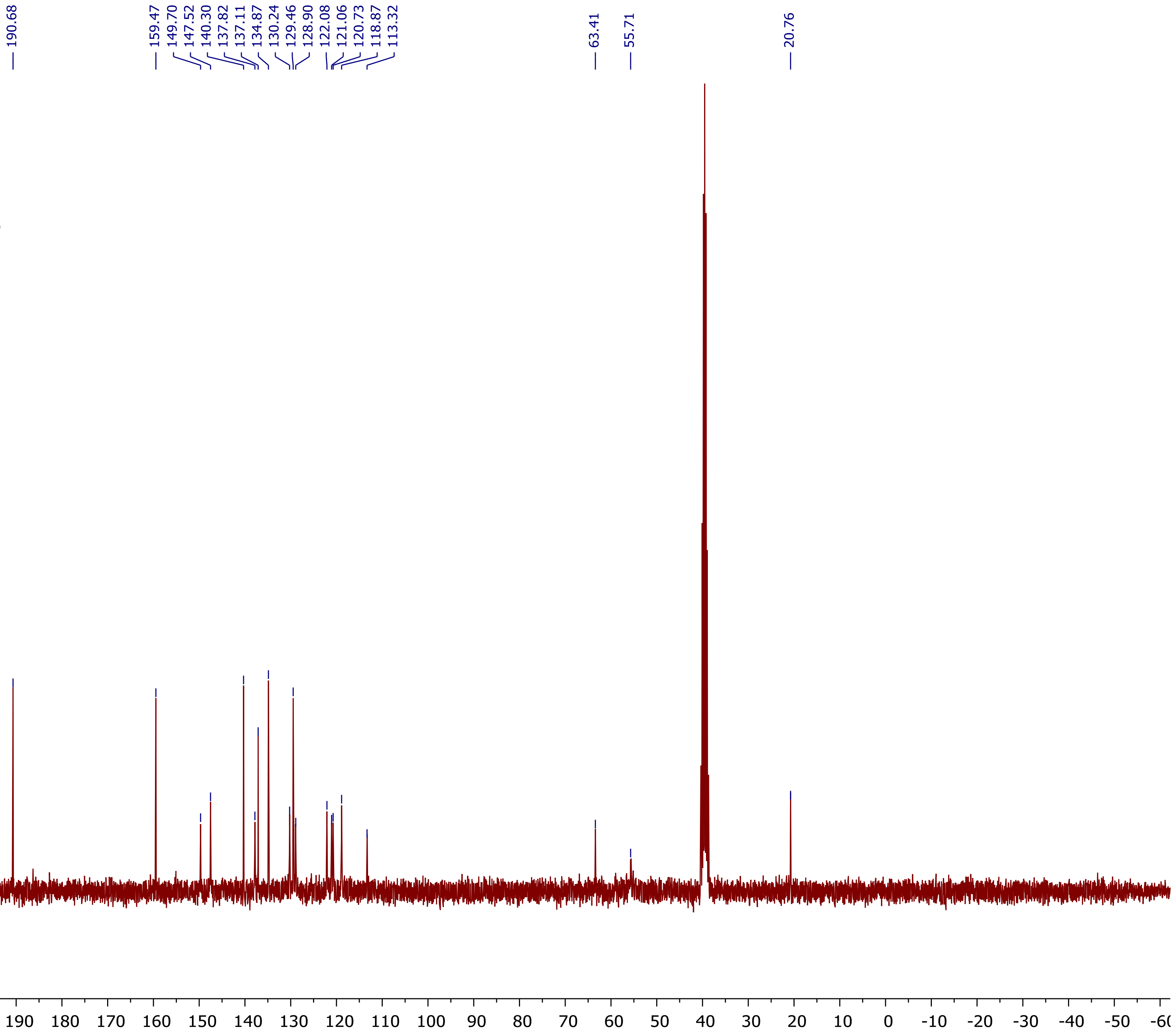

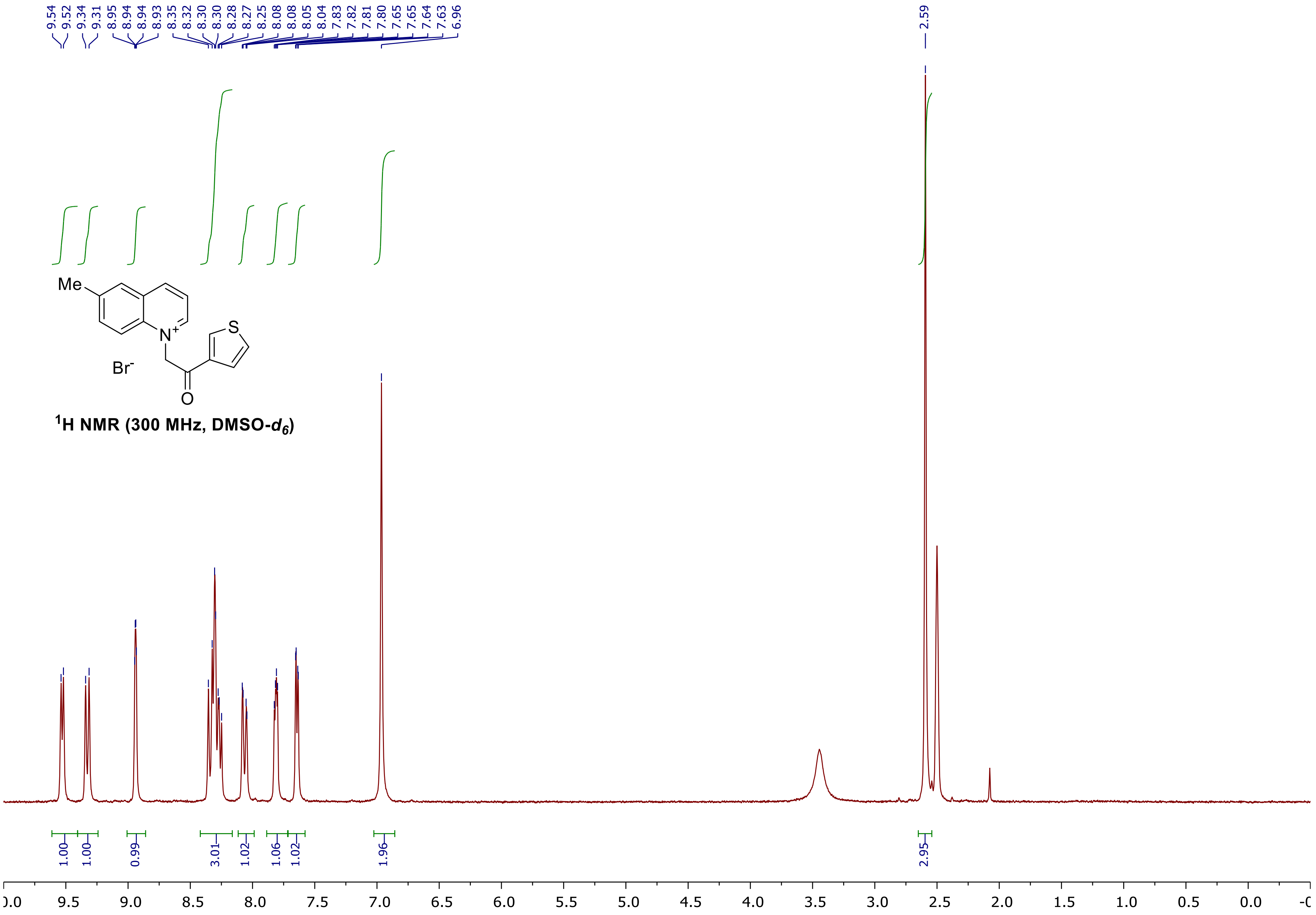

9.54 9.52 9.34 9.31 8.95 8.94 8.94 8.93 8.35 8.32 8.30 8.30 8.28 8.27 8.25 8.08 8.08 8.05 8.04 7.83 7.82 7.81 7.80 7.65 7.65 7.64 7.63 6.96
2.59
Me
N+
S
Br-
O
^{1}H NMR (300 MHz, DMSO-d_6)
1.00 1.00 0.99 3.01 1.02 1.06 1.02 1.96 2.95
9.5 9.0 8.5 8.0 7.5 7.0 6.5 6.0 5.5 5.0 4.5 4.0 3.5 3.0 2.5 2.0 1.5 1.0 0.5 0.0

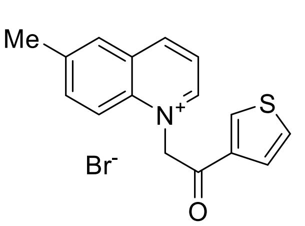

$^{13}C\{^{1}H\}$ NMR (75 MHz, DMSO-d_6)

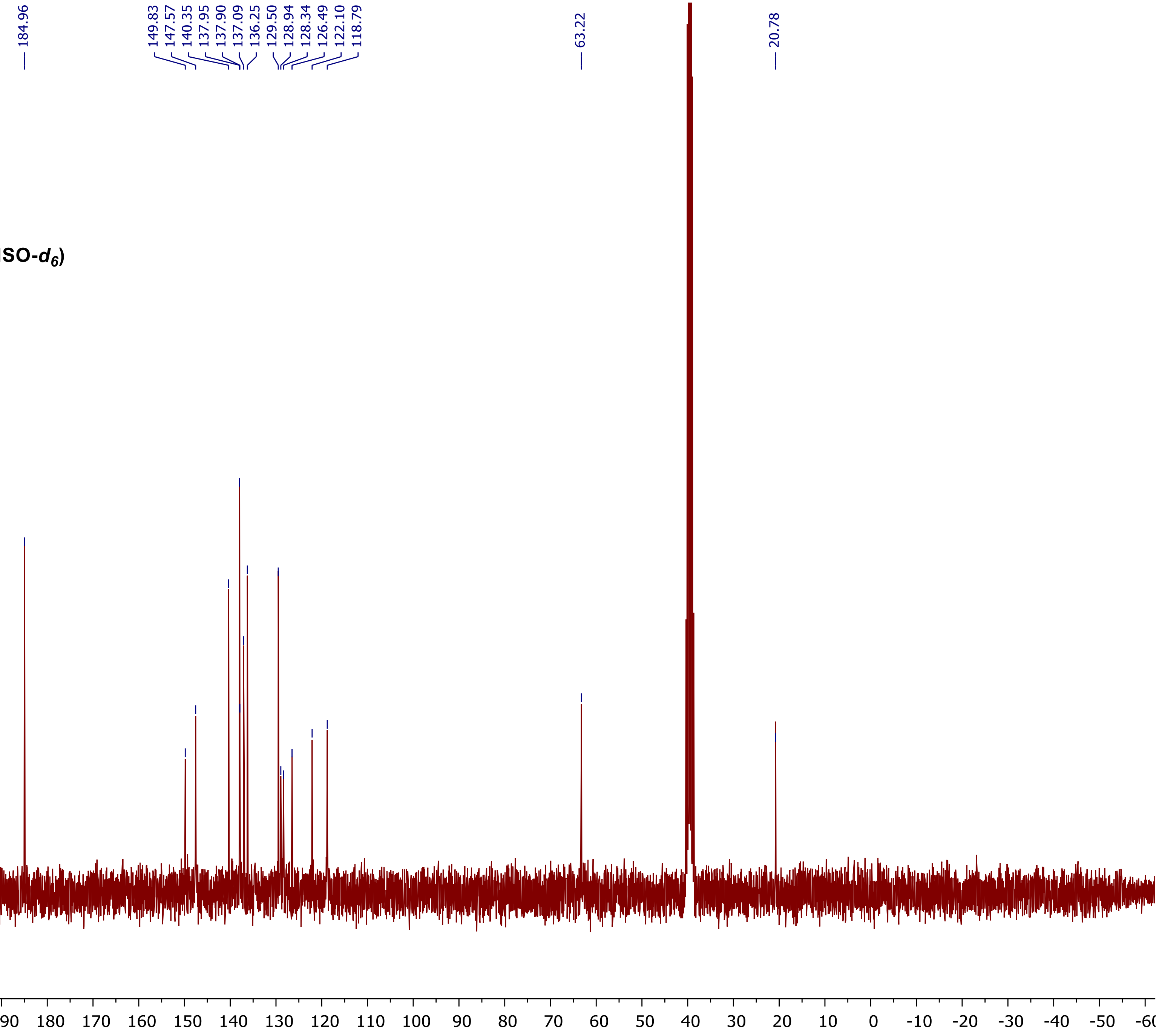

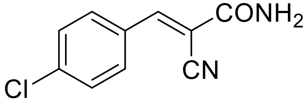

^{1}H NMR (300 MHz, DMSO-d_6)

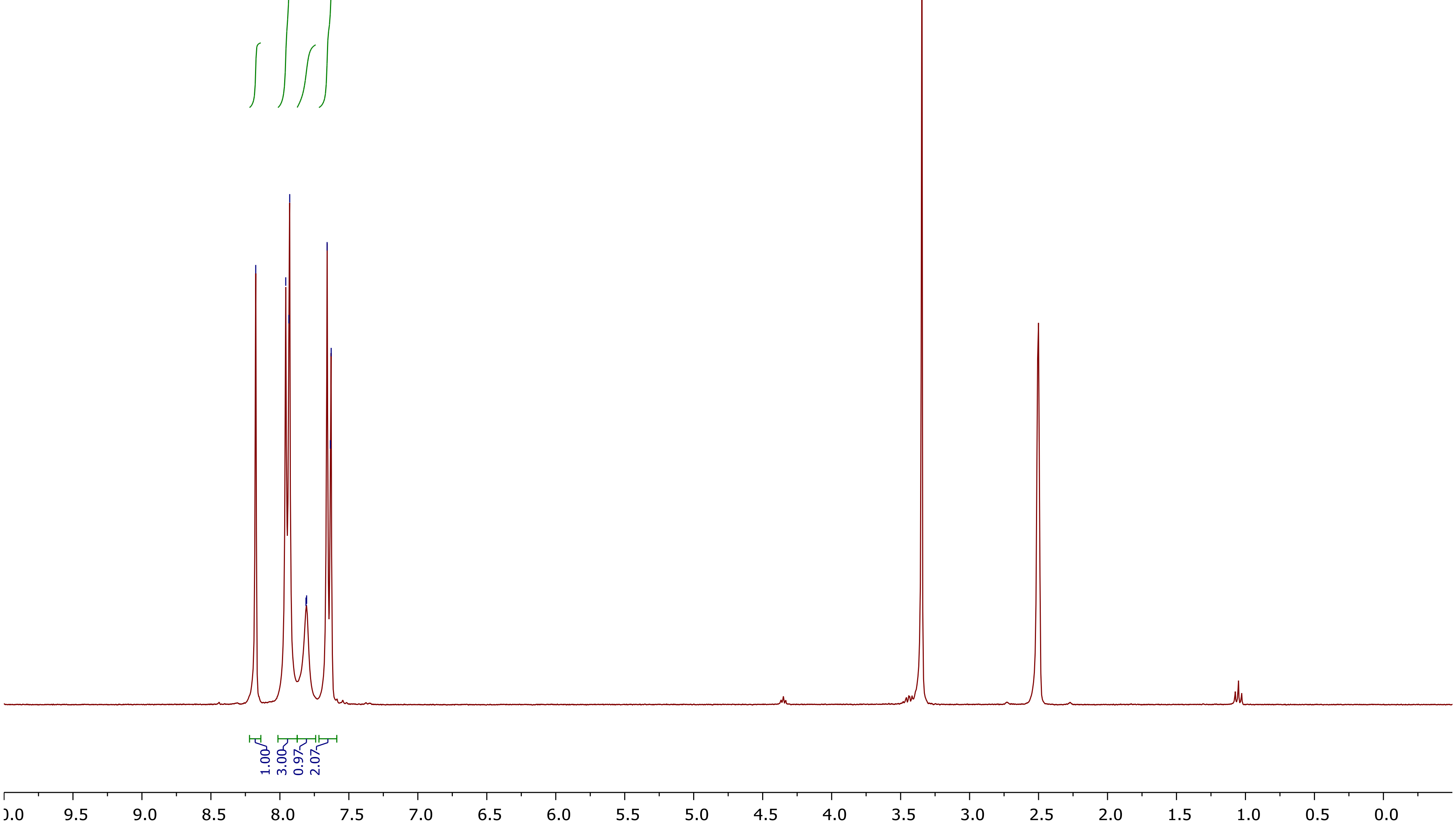

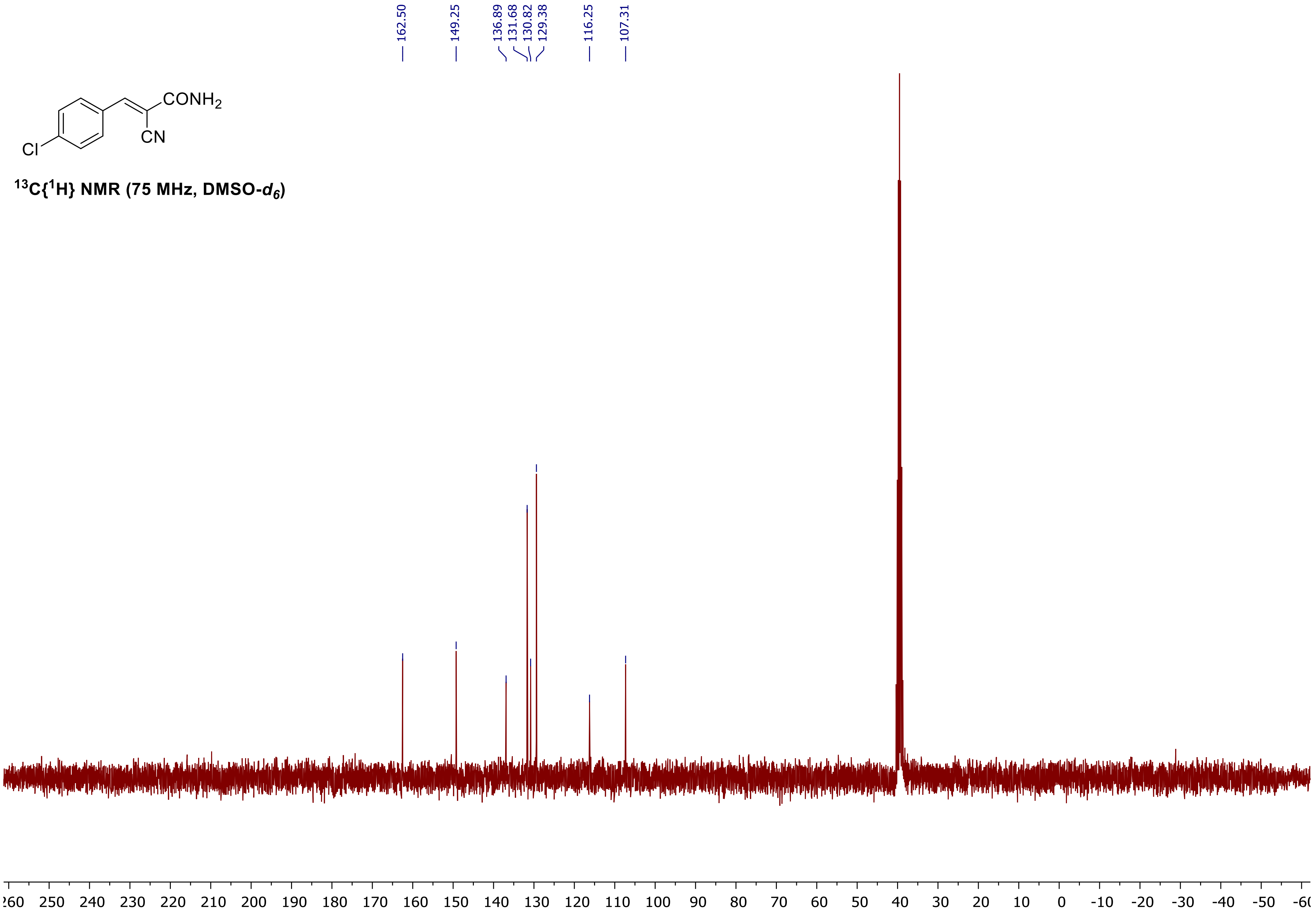
CONH2
CN
Cl
13C{1H} NMR (75 MHz, DMSO-d6)
162.50
149.25
136.89
131.68
130.82
129.38
116.25
107.31
260 250 240 230 220 210 200 190 180 170 160 150 140 130 120 110 100 90 80 70 60 50 40 30 20 10 0 -10 -20 -30 -40 -50 -60

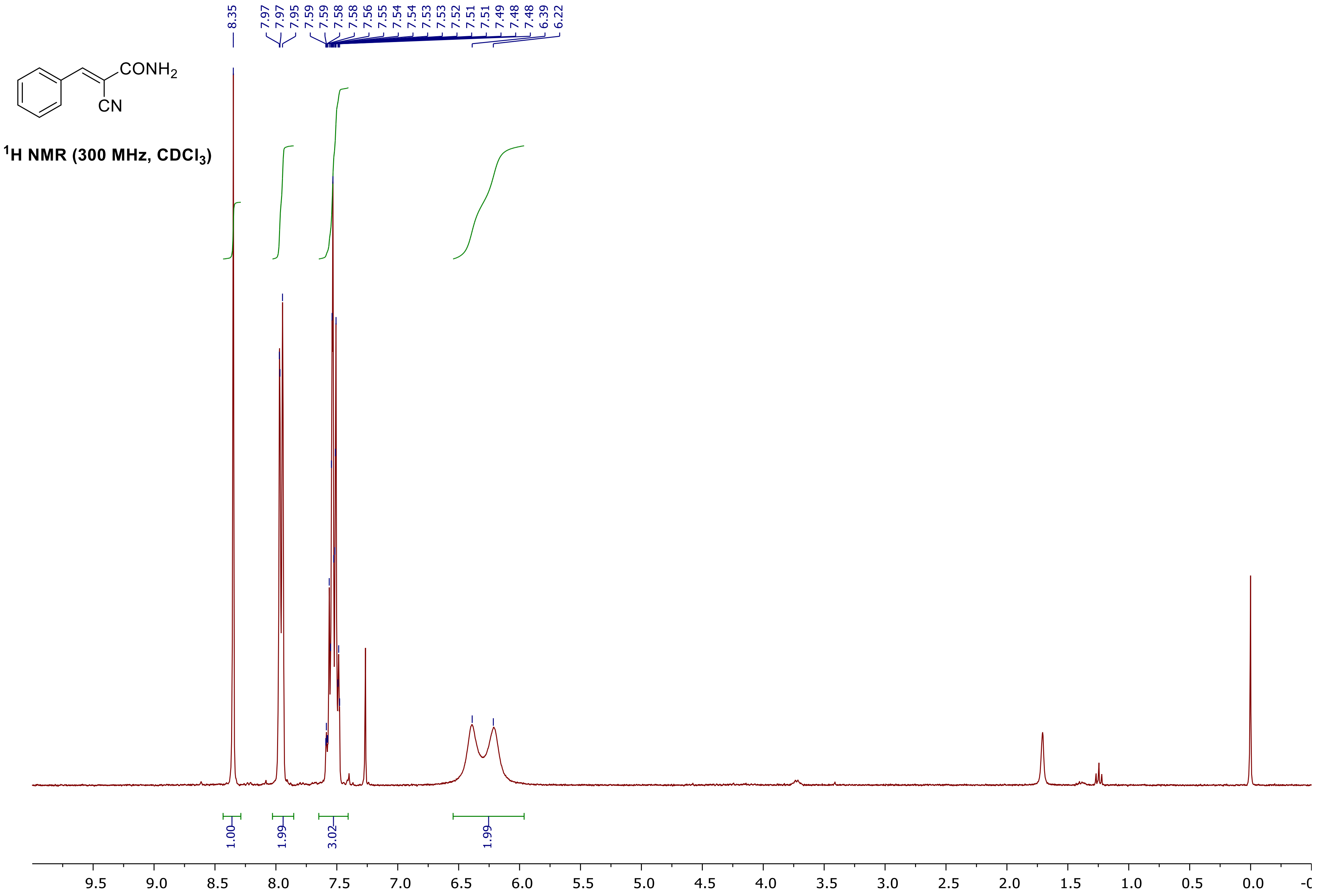
CONH2
CN
1H NMR (300 MHz, CDCl3)
8.35
7.97 7.97 7.95 7.59 7.59 7.58 7.58 7.56 7.55 7.54 7.54 7.53 7.53 7.52 7.51 7.51 7.49 7.48 7.48 6.39 6.22
1.00 1.99 3.02 1.99
9.5 9.0 8.5 8.0 7.5 7.0 6.5 6.0 5.5 5.0 4.5 4.0 3.5 3.0 2.5 2.0 1.5 1.0 0.5 0.0

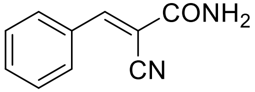

$^{13}C\{^{1}H\}$ NMR (75 MHz, $CDCl_3$)

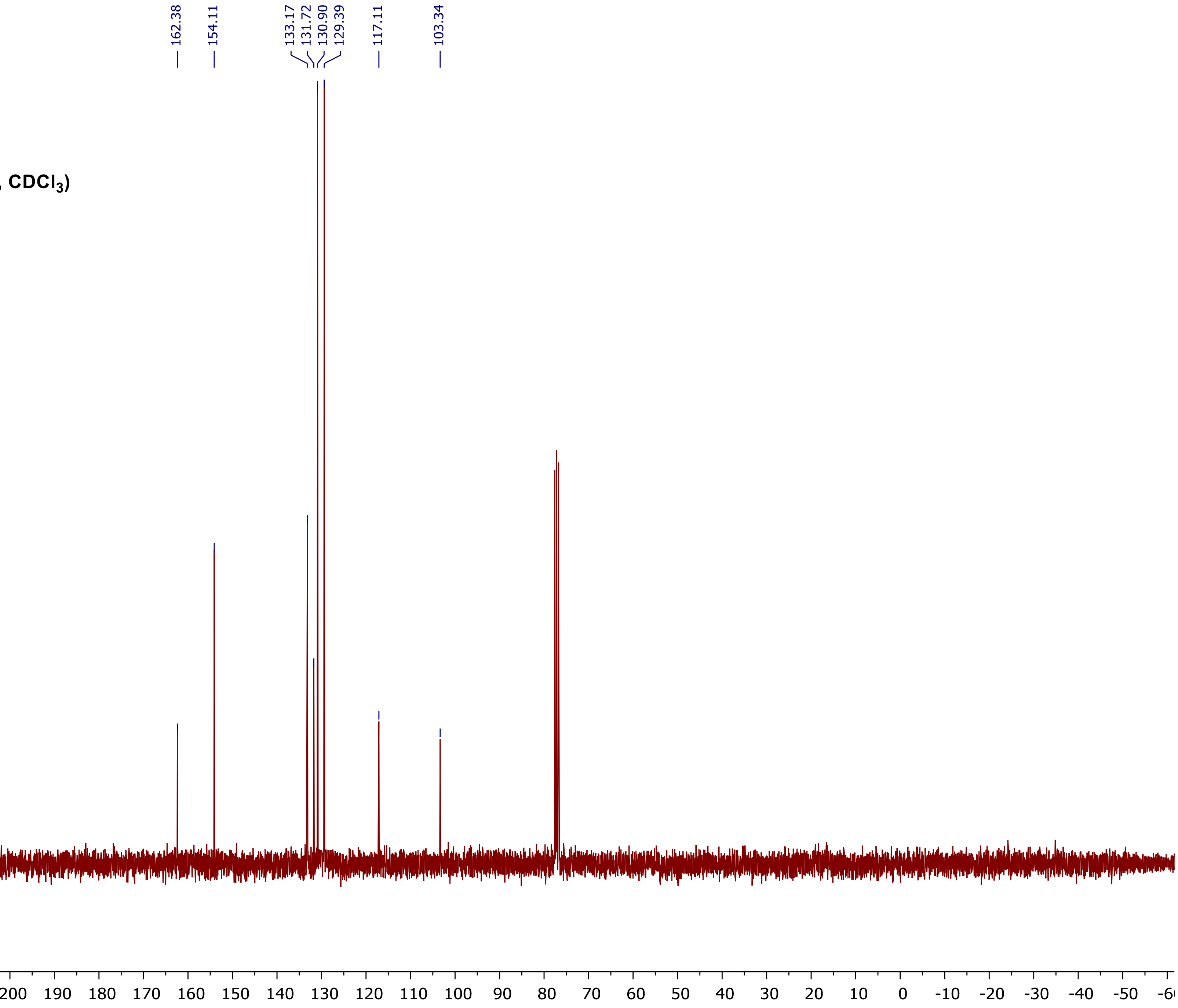

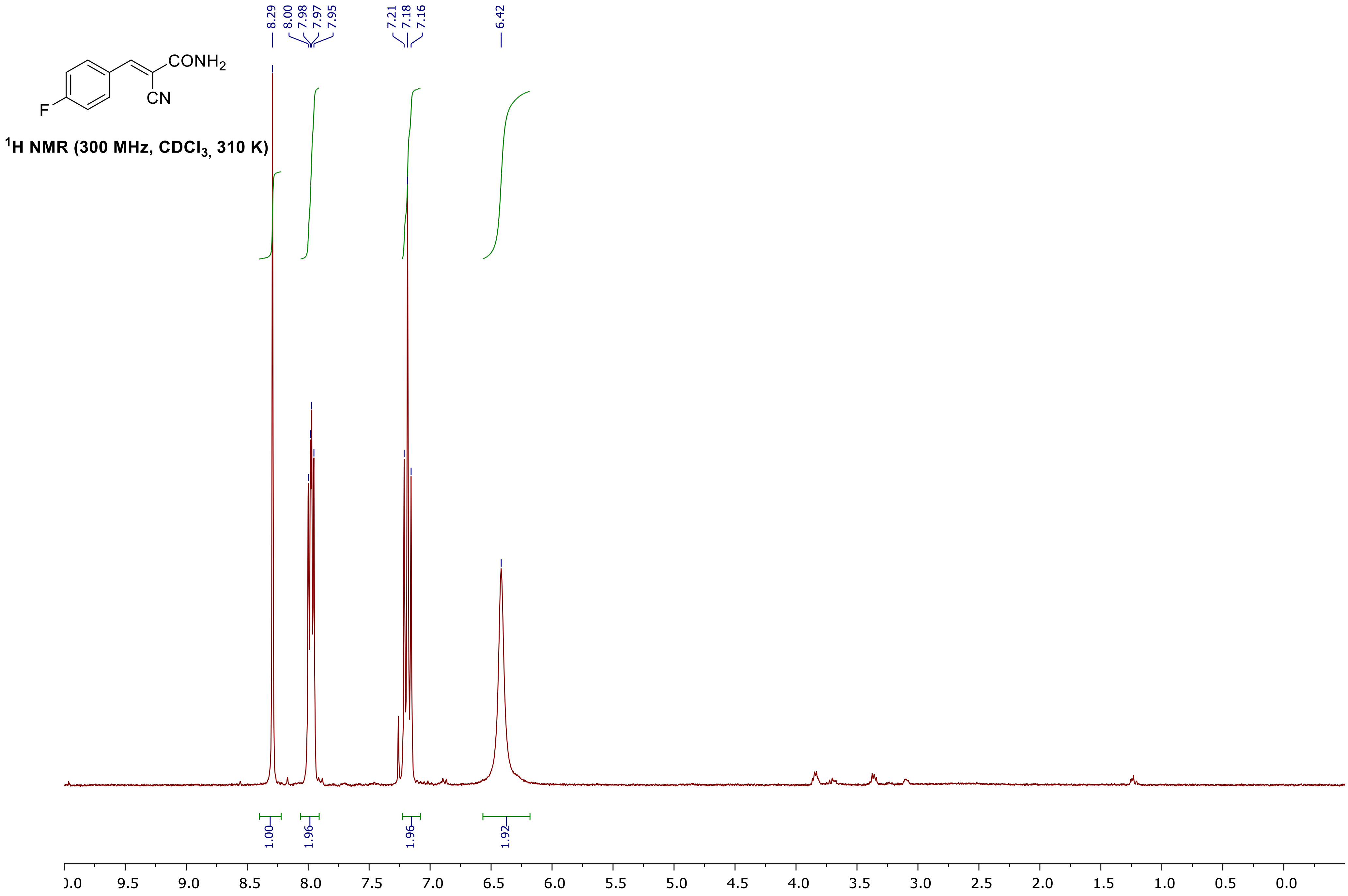
CONH2
CN
F
1H NMR (300 MHz, CDCl3, 310 K)
8.29
8.00
7.98
7.97
7.95
7.21
7.18
7.16
6.42
1.00
1.96
1.96
1.92
).0 9.5 9.0 8.5 8.0 7.5 7.0 6.5 6.0 5.5 5.0 4.5 4.0 3.5 3.0 2.5 2.0 1.5 1.0 0.5 0.0

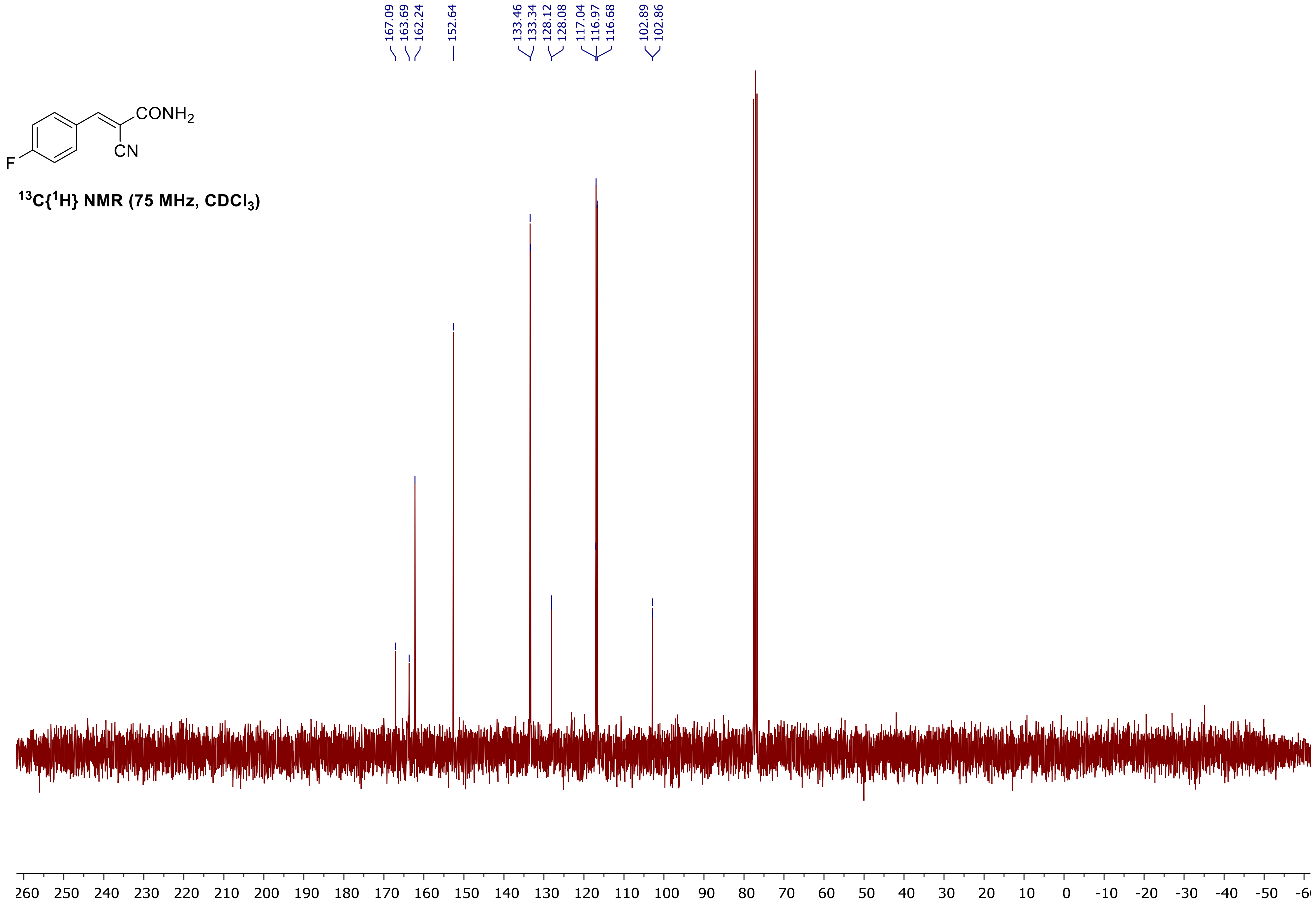
167.09
163.69
162.24
152.64
133.46
133.34
128.12
128.08
117.04
116.97
116.68
102.89
102.86
CONH2
CN
F
13C{1H} NMR (75 MHz, CDCl3)
260 250 240 230 220 210 200 190 180 170 160 150 140 130 120 110 100 90 80 70 60 50 40 30 20 10 0 -10 -20 -30 -40 -50

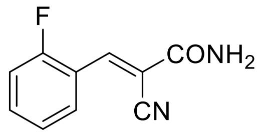

^1H NMR (300 MHz, $CDCl_3$)

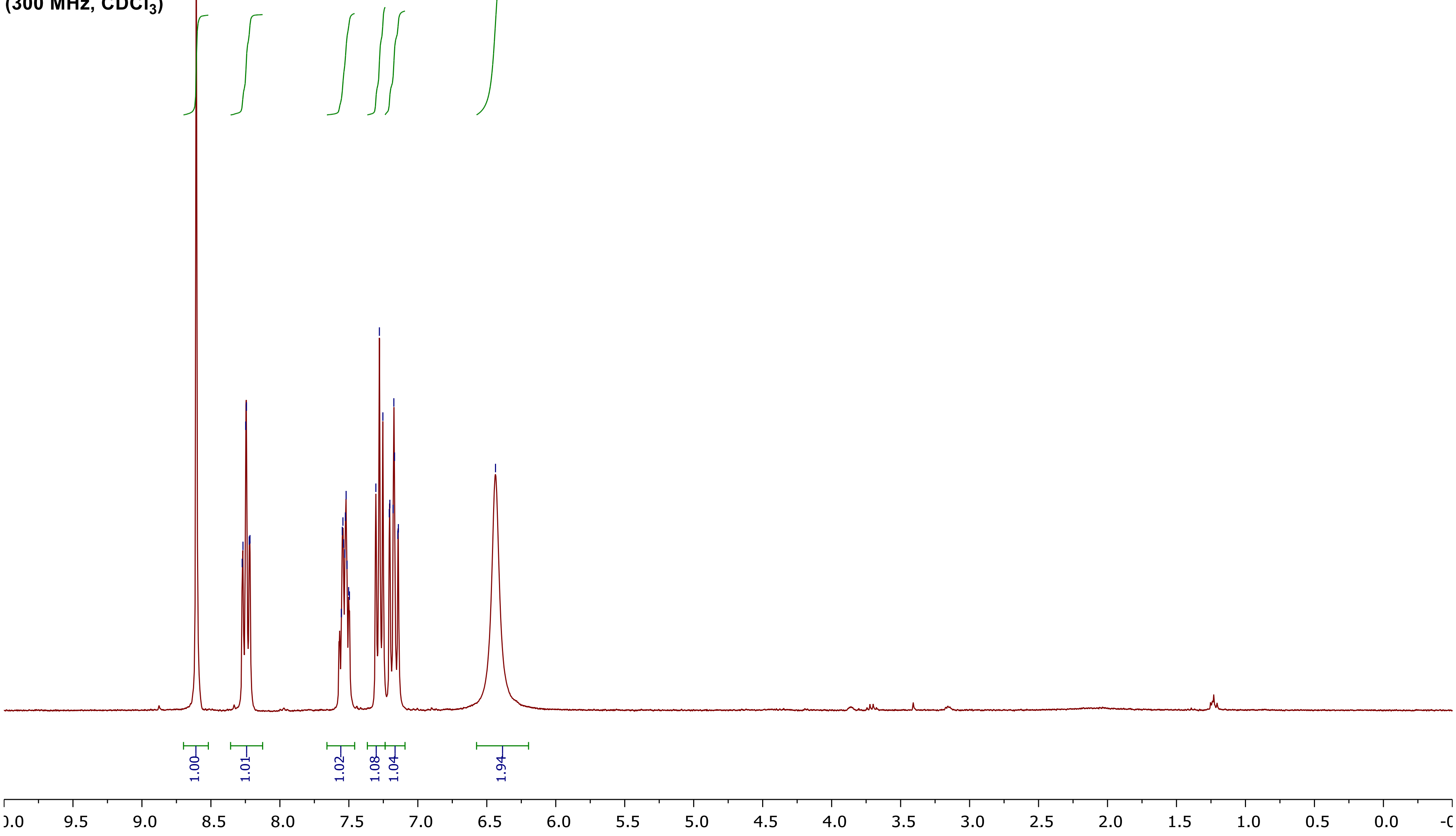

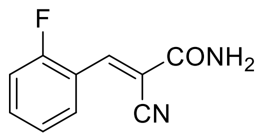

$^{13}C\{^{1}H\}$ NMR (75 MHz, $CDCl_3$)

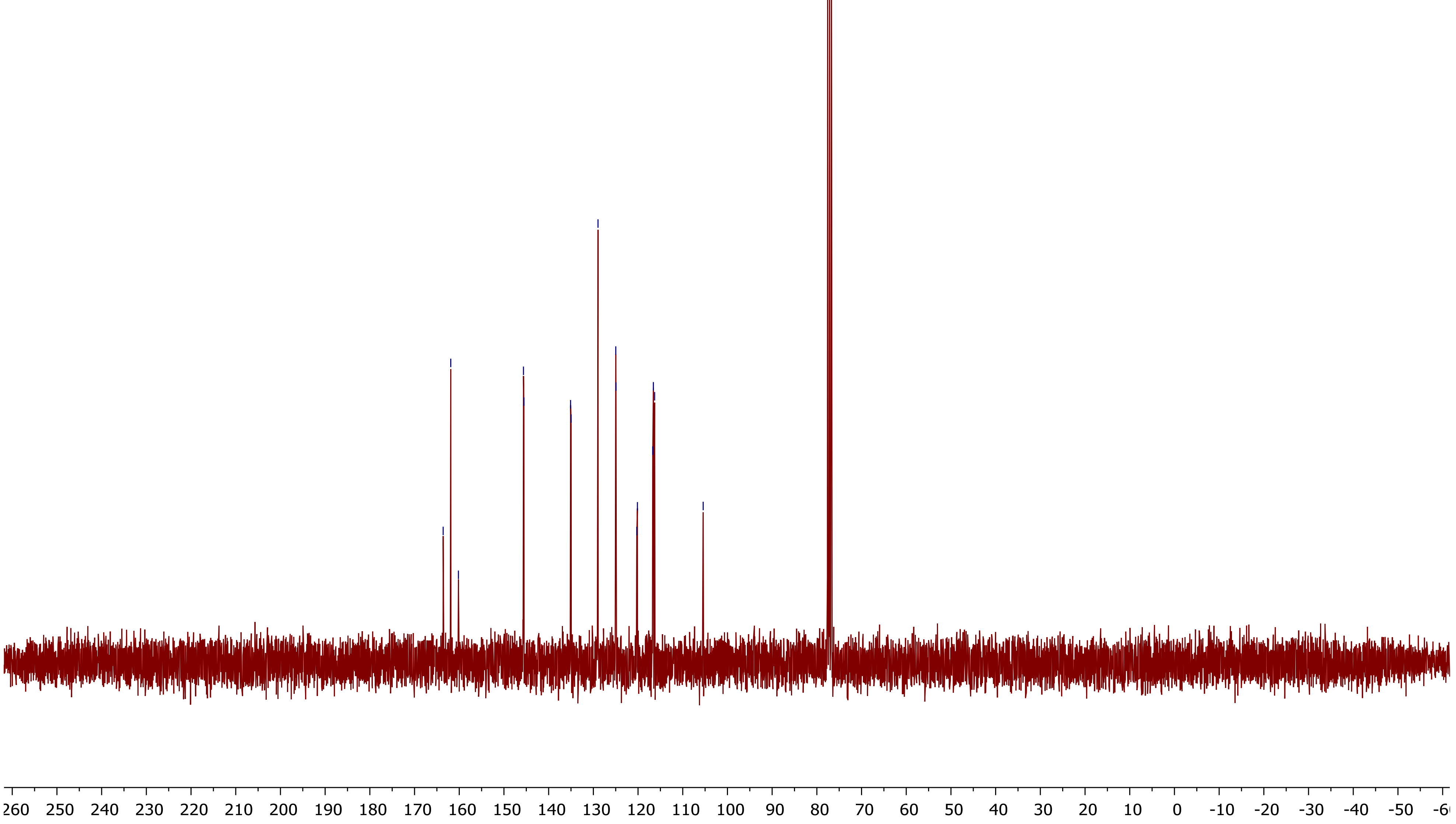

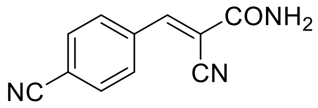

^{1}H NMR (300 MHz, DMSO-d_6)

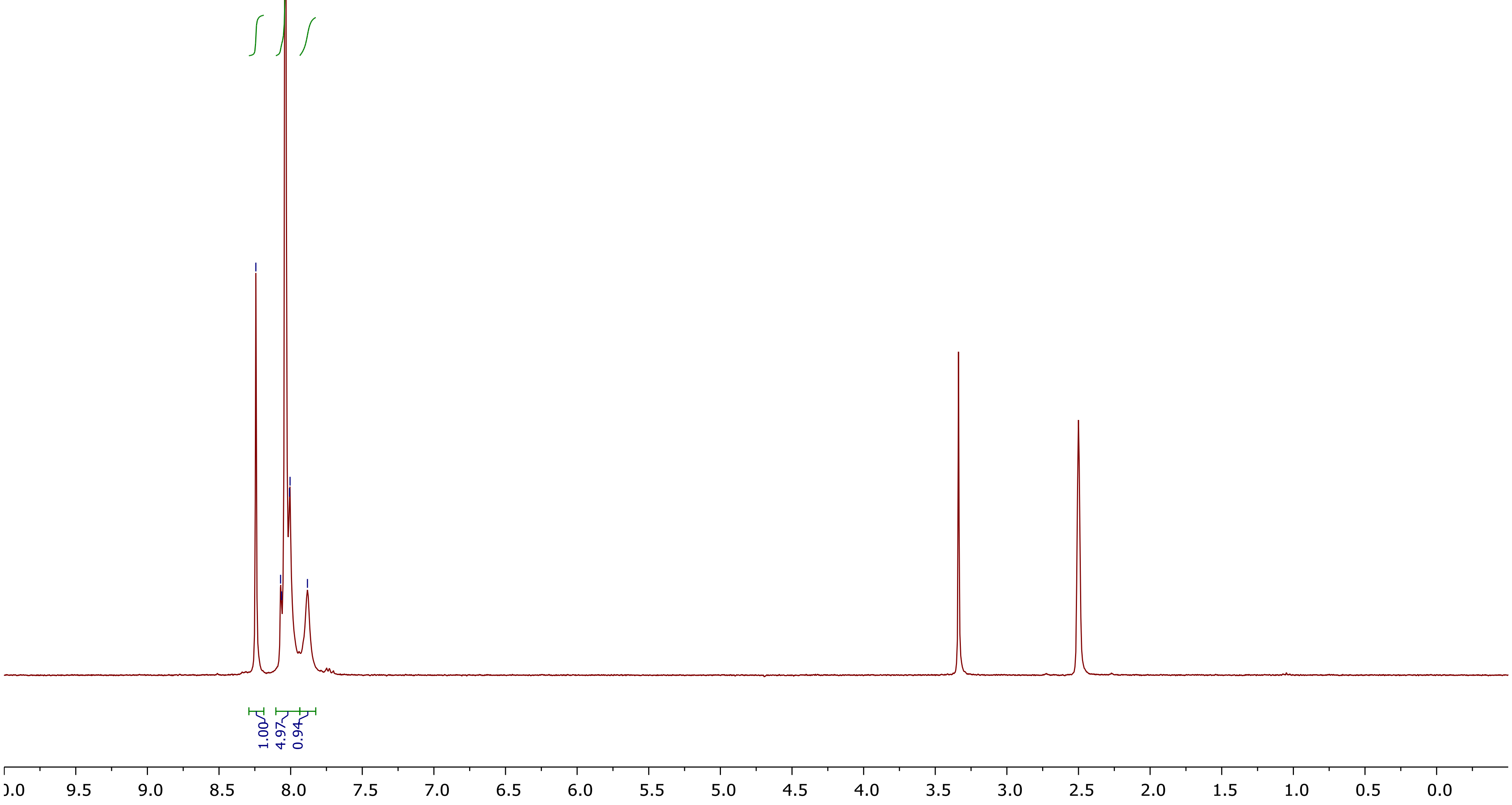

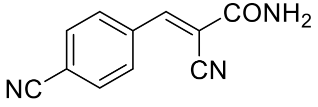

$^{13}C\{^{1}H\}$ **NMR (75 MHz, DMSO-d_6)**

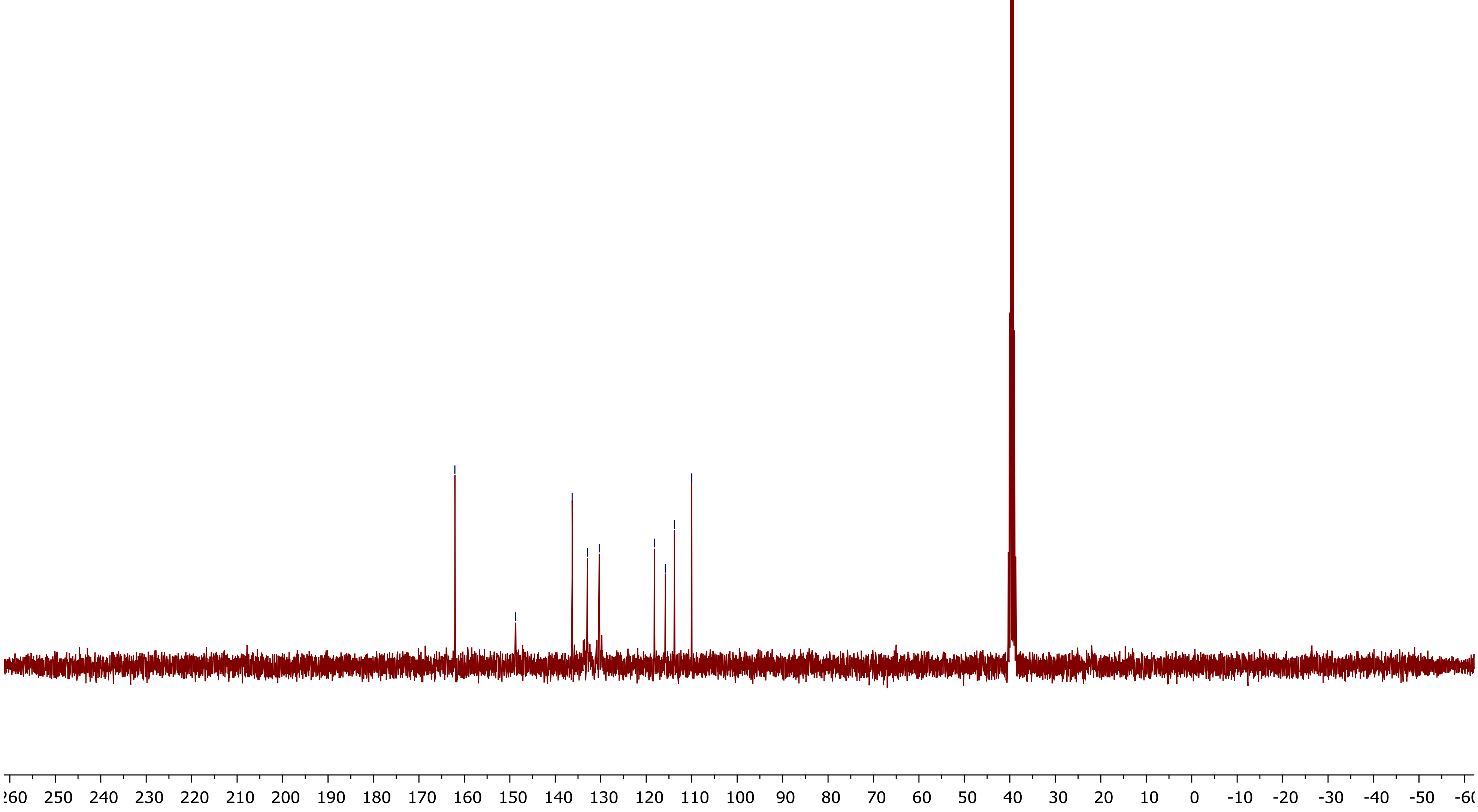

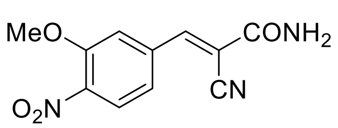

^{1}**H NMR (300 MHz, DMSO-d_6)**

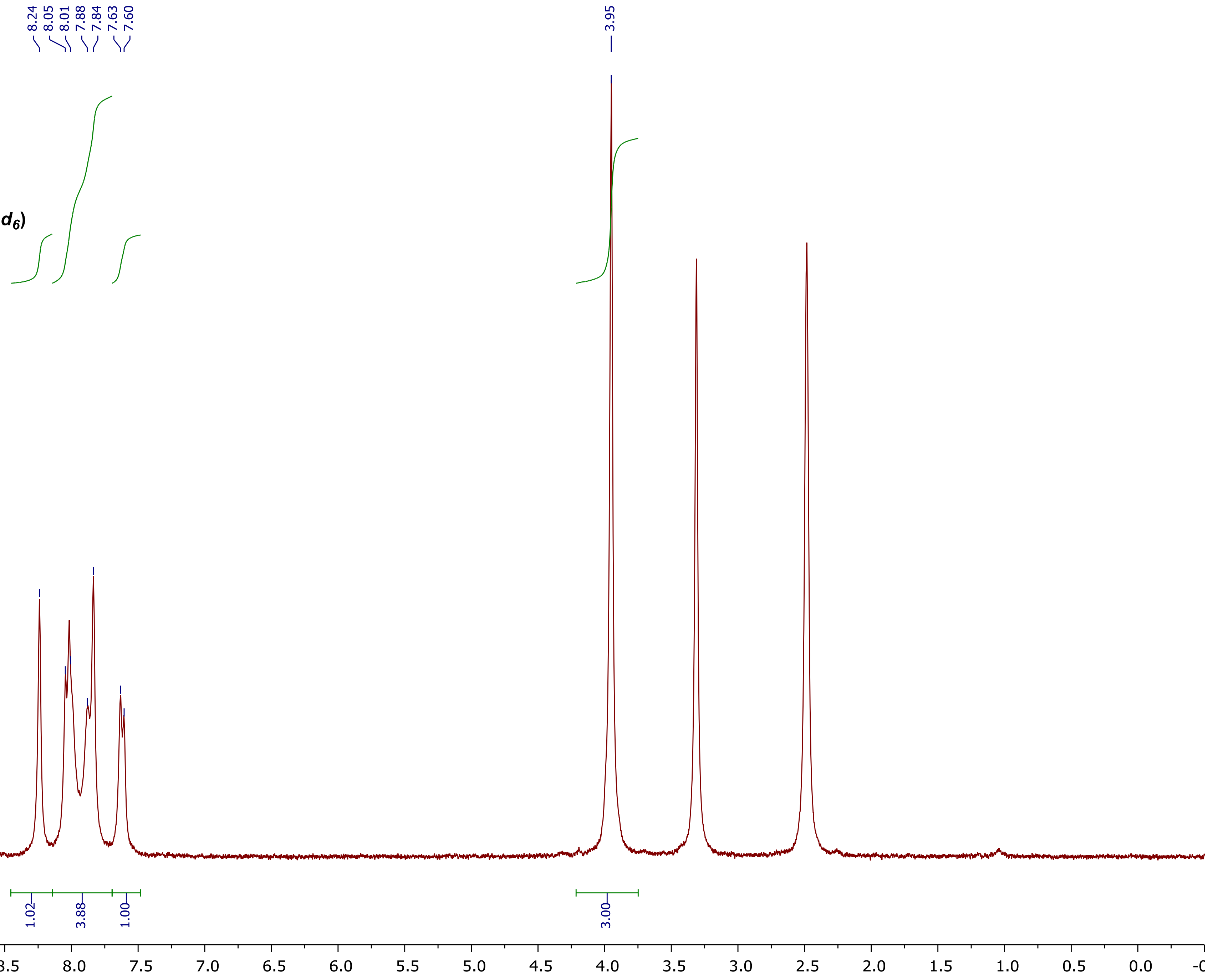

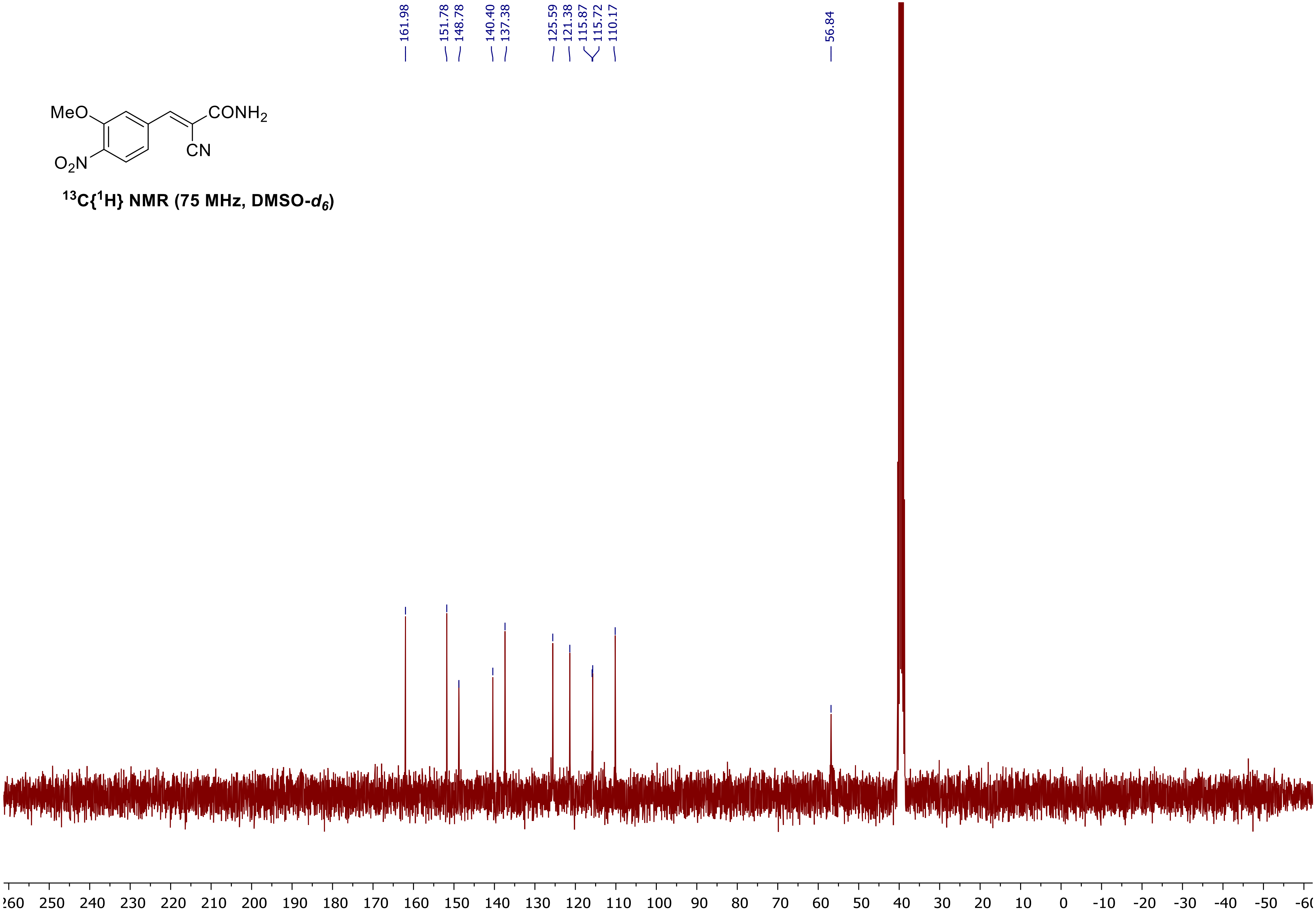

$^{13}C\{^{1}H\}$ NMR (75 MHz, DMSO-d_6)

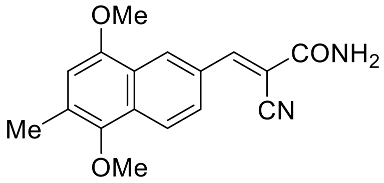

^{1}H NMR (300 MHz, DMSO-d_6)

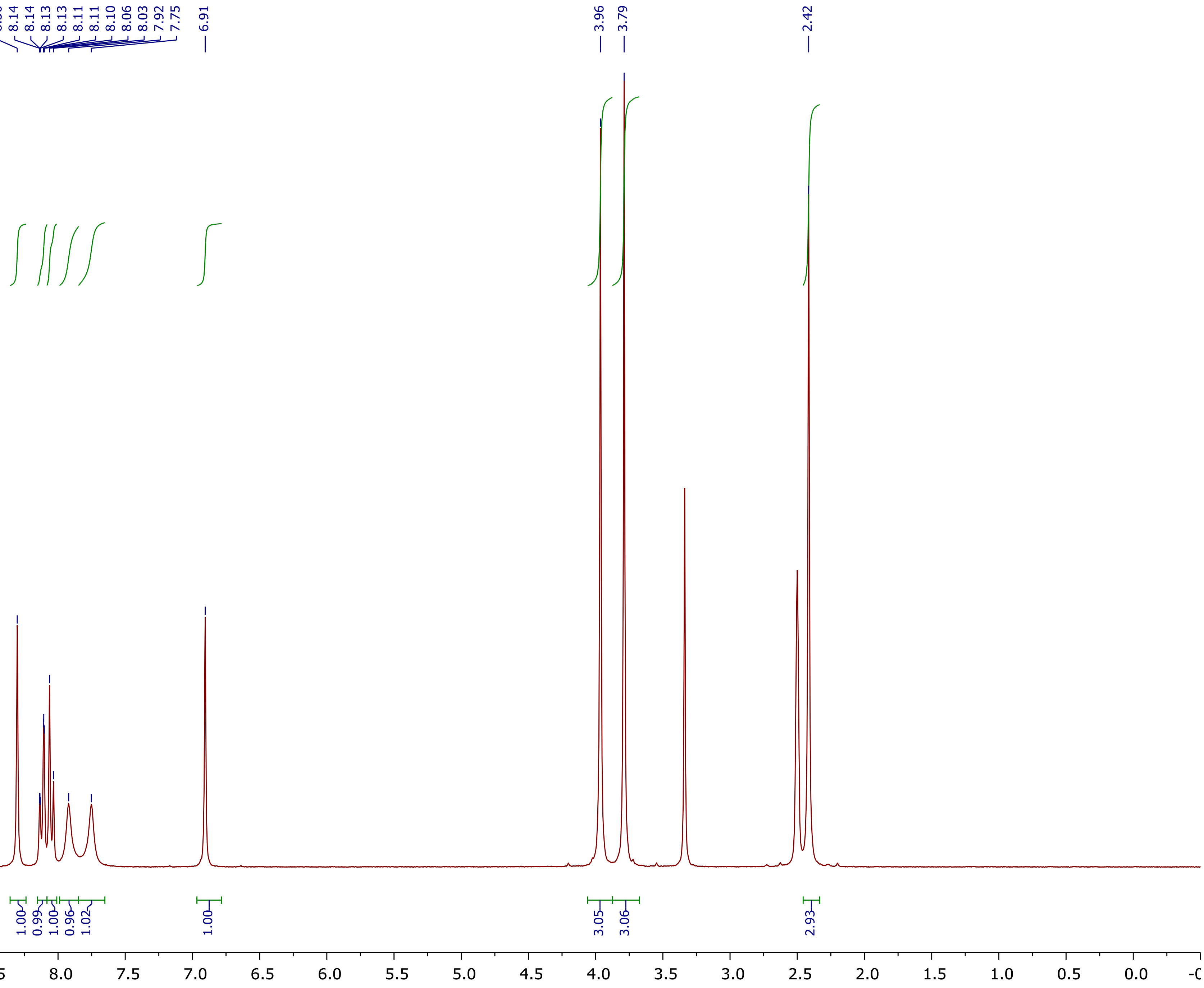

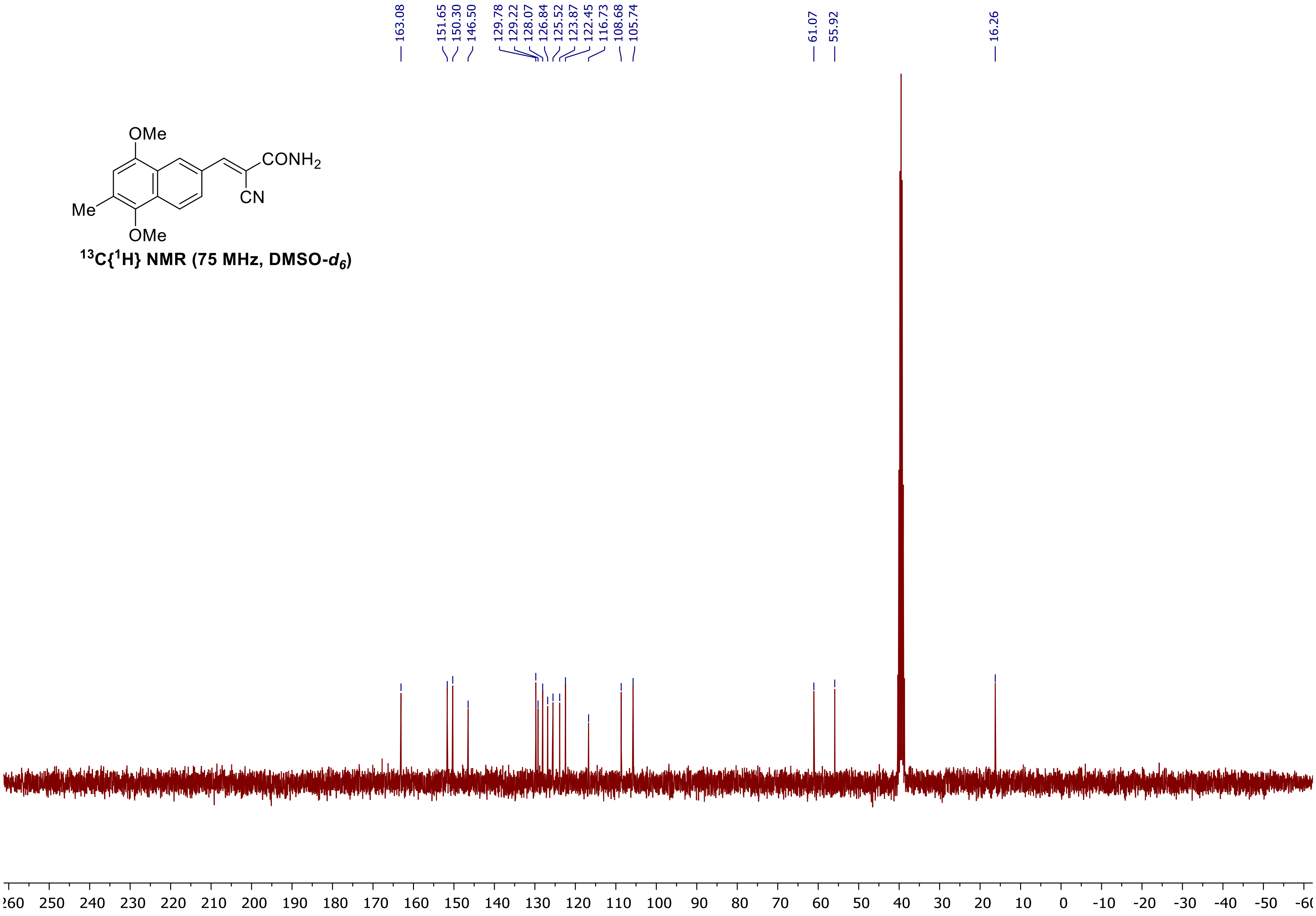

$^{13}C\{^{1}H\}$ NMR (75 MHz, DMSO-d_6)

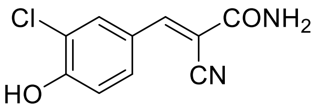

^{1}H NMR (300 MHz, DMSO-d_6)

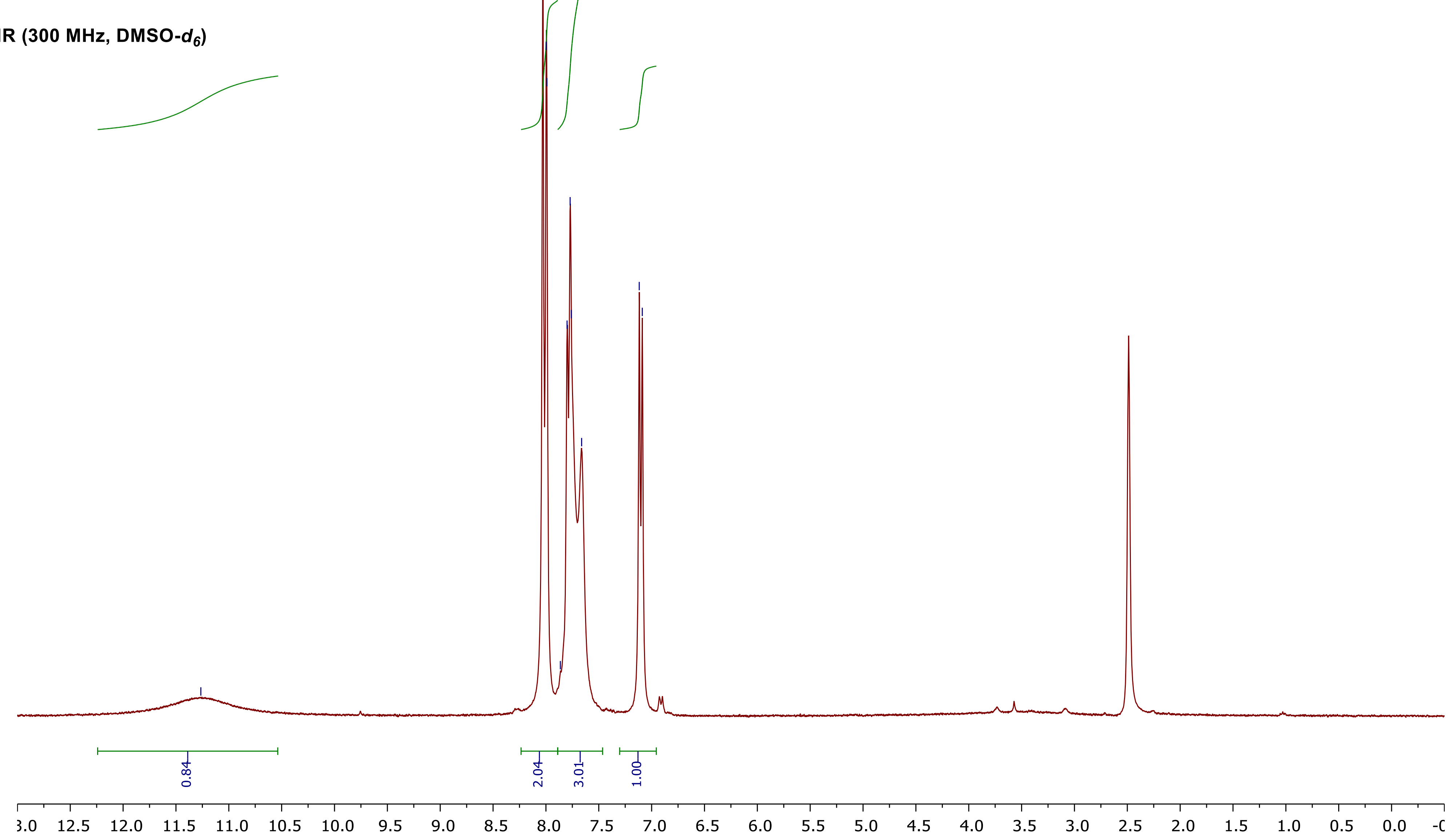

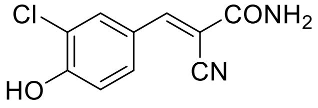

$^{13}C\{^1H\}$ NMR (75 MHz, DMSO-d_6)

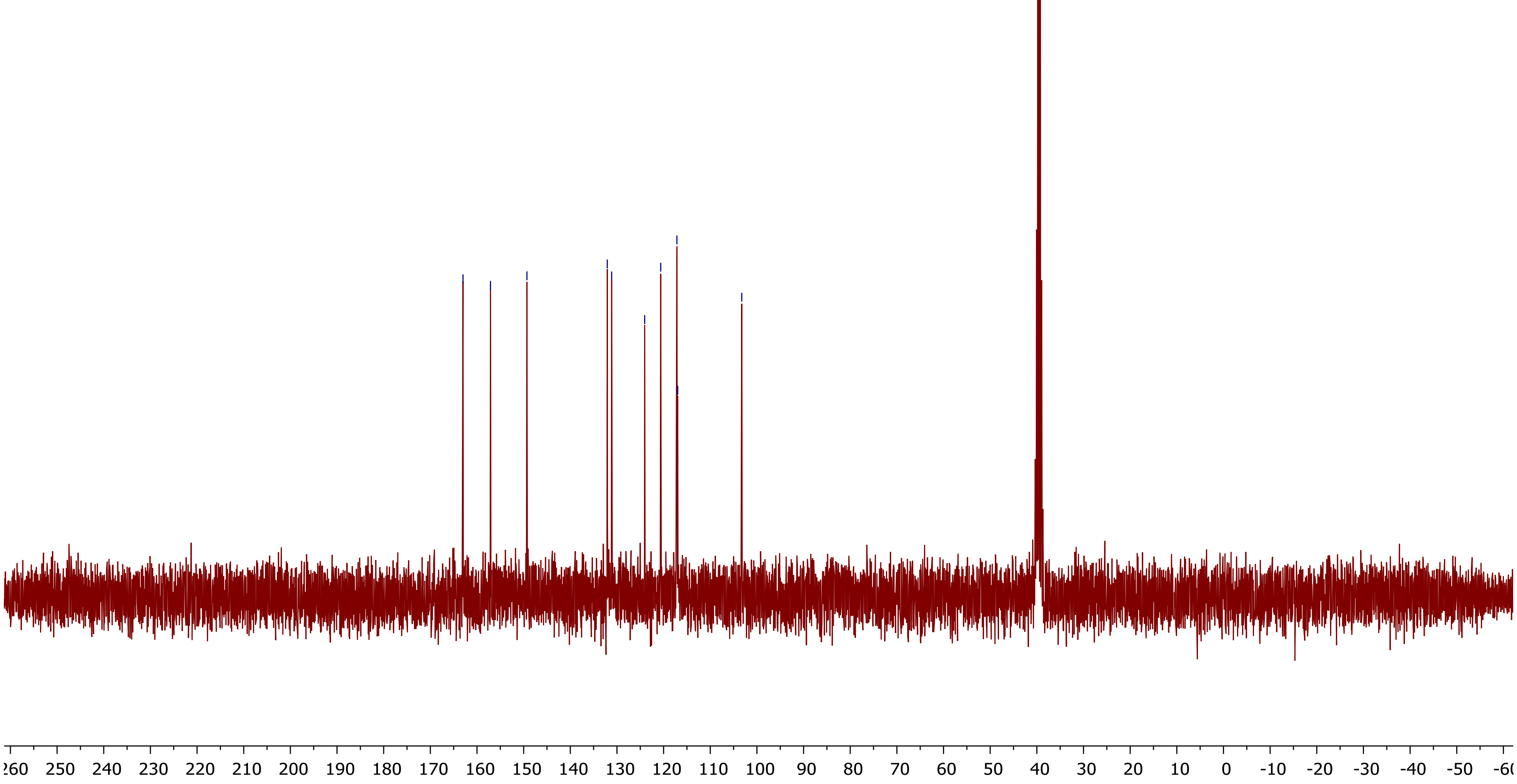

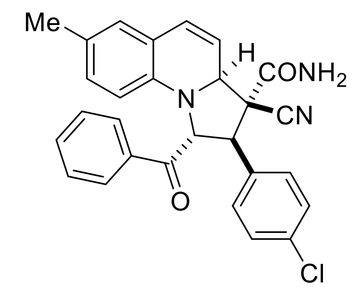

^{1}H NMR (300 MHz, CDCl$_3$)

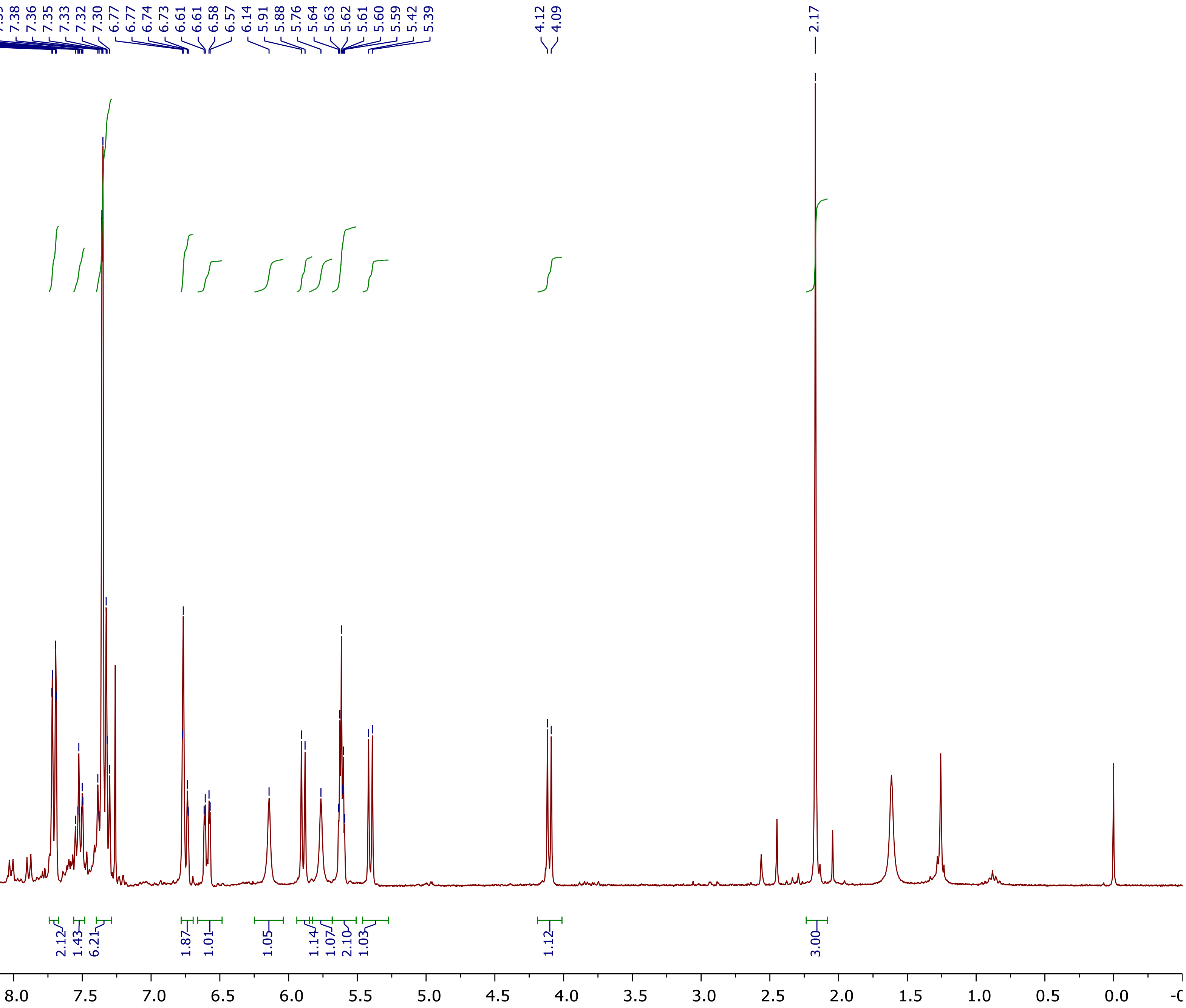

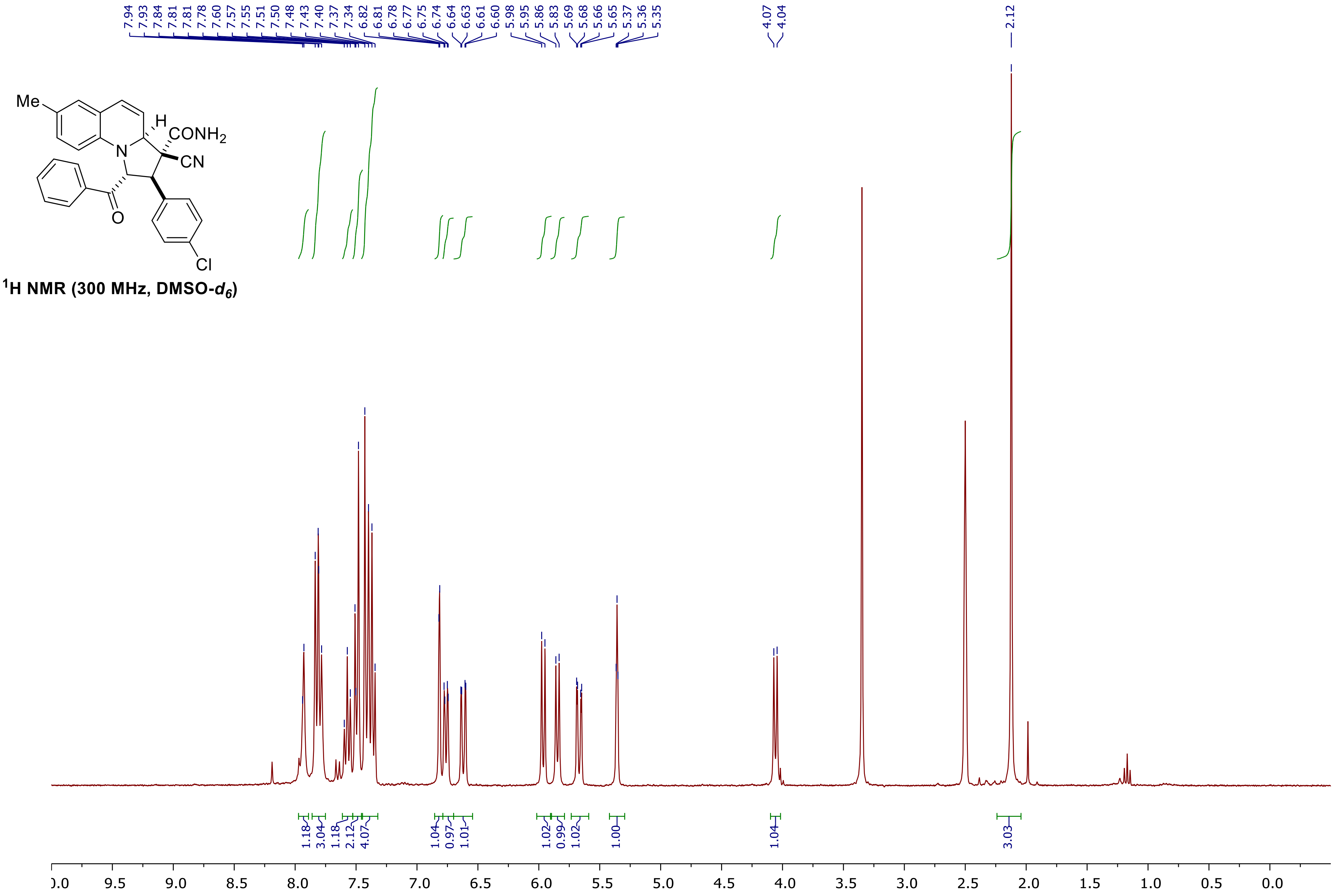

7.94
7.93
7.84
7.81
7.81
7.78
7.60
7.57
7.55
7.51
7.50
7.48
7.43
7.40
7.37
7.34
6.82
6.81
6.78
6.77
6.75
6.74
6.64
6.63
6.61
6.60
5.98
5.95
5.86
5.83
5.69
5.68
5.66
5.65
5.37
5.36
5.35
4.07
4.04
2.12
Me
H
CONH2
N
CN
O
Cl
1H NMR (300 MHz, DMSO-d6)
1.18
3.04
1.18
2.12
4.07
1.04
0.97
1.01
1.02
0.99
1.02
1.00
1.04
3.03
10.0
9.5
9.0
8.5
8.0
7.5
7.0
6.5
6.0
5.5
5.0
4.5
4.0
3.5
3.0
2.5
2.0
1.5
1.0
0.5
0.0

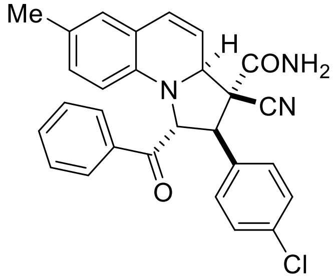

$^{13}C\{^{1}H\}$ **NMR (75 MHz, DMSO-*d*$_6$)**

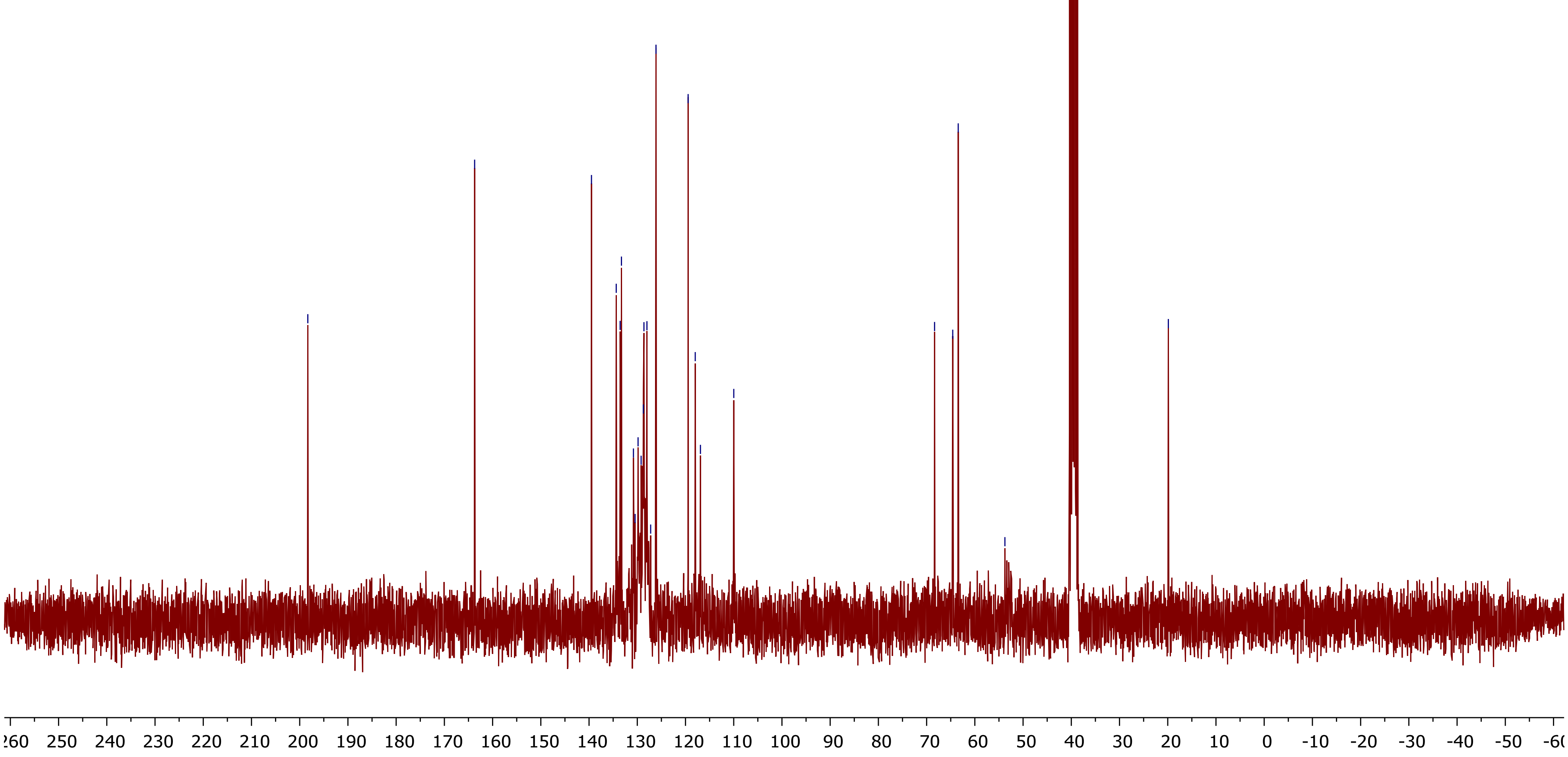

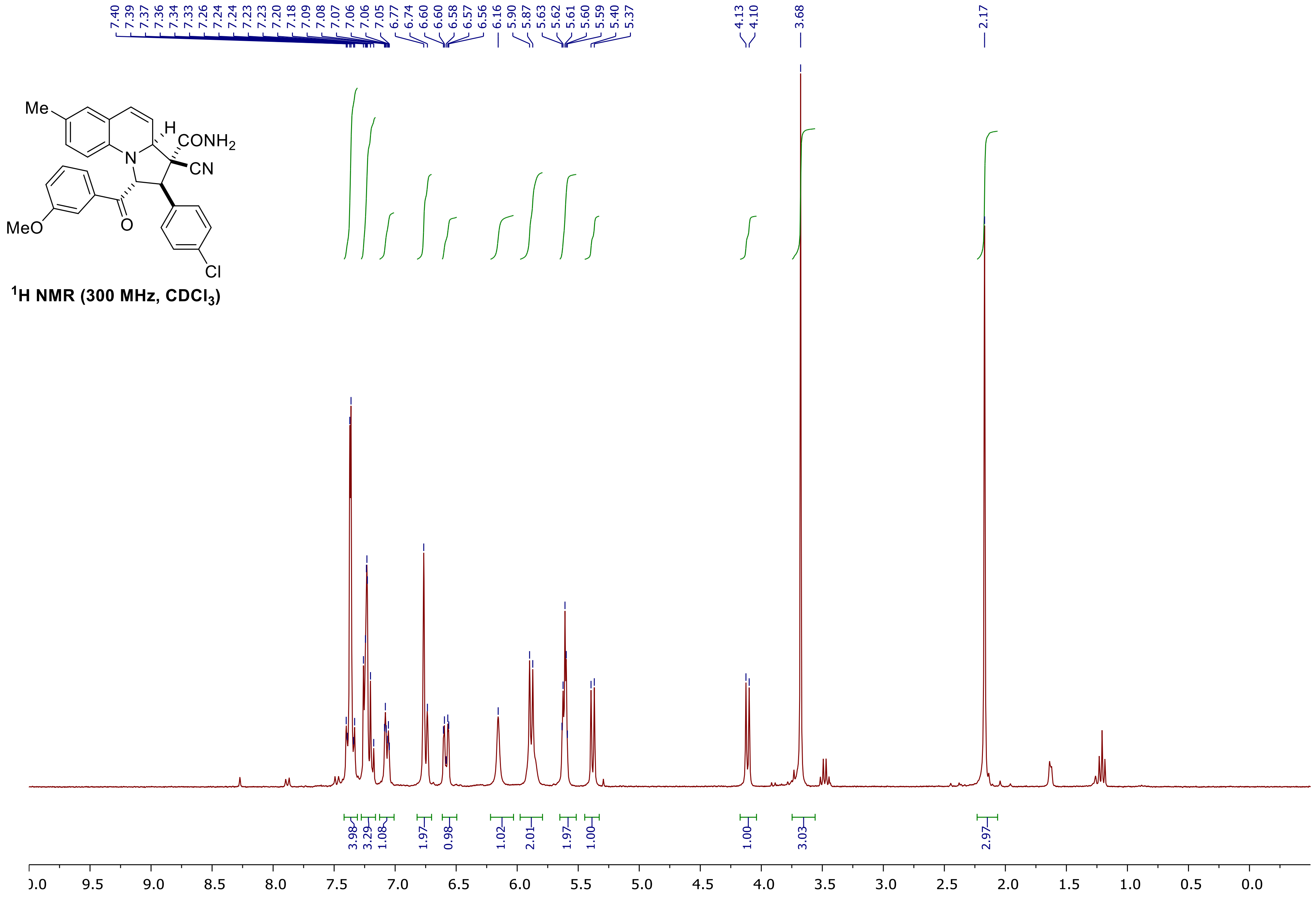
Me
H
CONH2
N
CN
MeO
O
Cl
1H NMR (300 MHz, CDCl3)
7.40
7.39
7.37
7.36
7.34
7.33
7.26
7.24
7.24
7.23
7.23
7.20
7.18
7.09
7.08
7.07
7.06
7.06
7.05
6.77
6.74
6.60
6.60
6.58
6.57
6.56
6.16
5.90
5.87
5.63
5.62
5.61
5.60
5.59
5.40
5.37
4.13
4.10
3.68
2.17
3.98
3.29
1.08
1.97
0.98
1.02
2.01
1.97
1.00
1.00
3.03
2.97
10.0
9.5
9.0
8.5
8.0
7.5
7.0
6.5
6.0
5.5
5.0
4.5
4.0
3.5
3.0
2.5
2.0
1.5
1.0
0.5
0.0

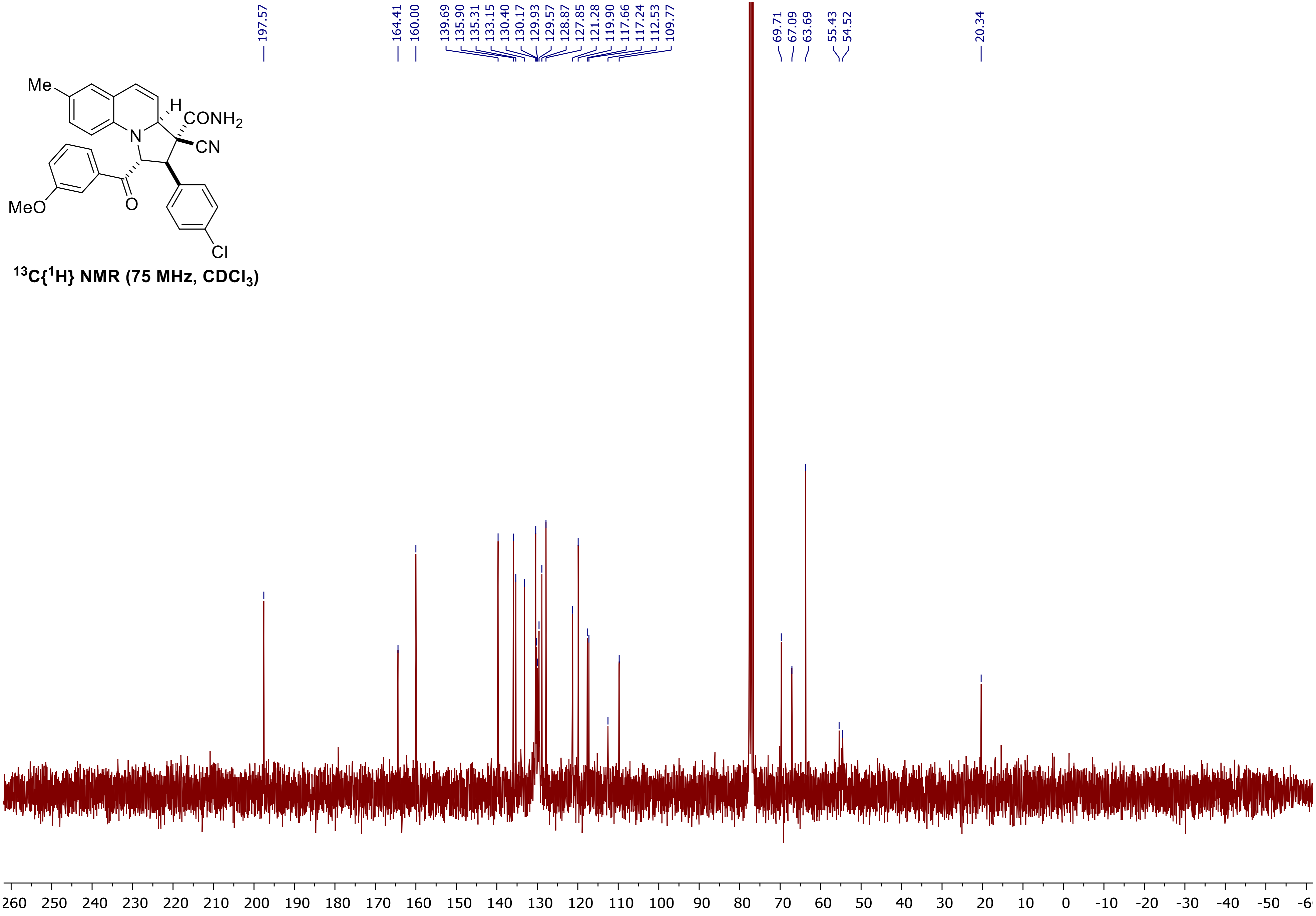

$^{13}C\{^{1}H\}$ NMR (75 MHz, $CDCl_3$)

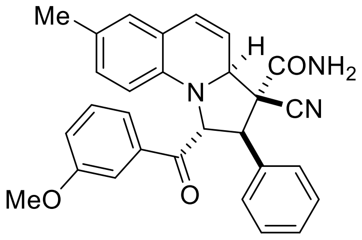

^{1}H NMR (300 MHz, DMSO-*d*6)

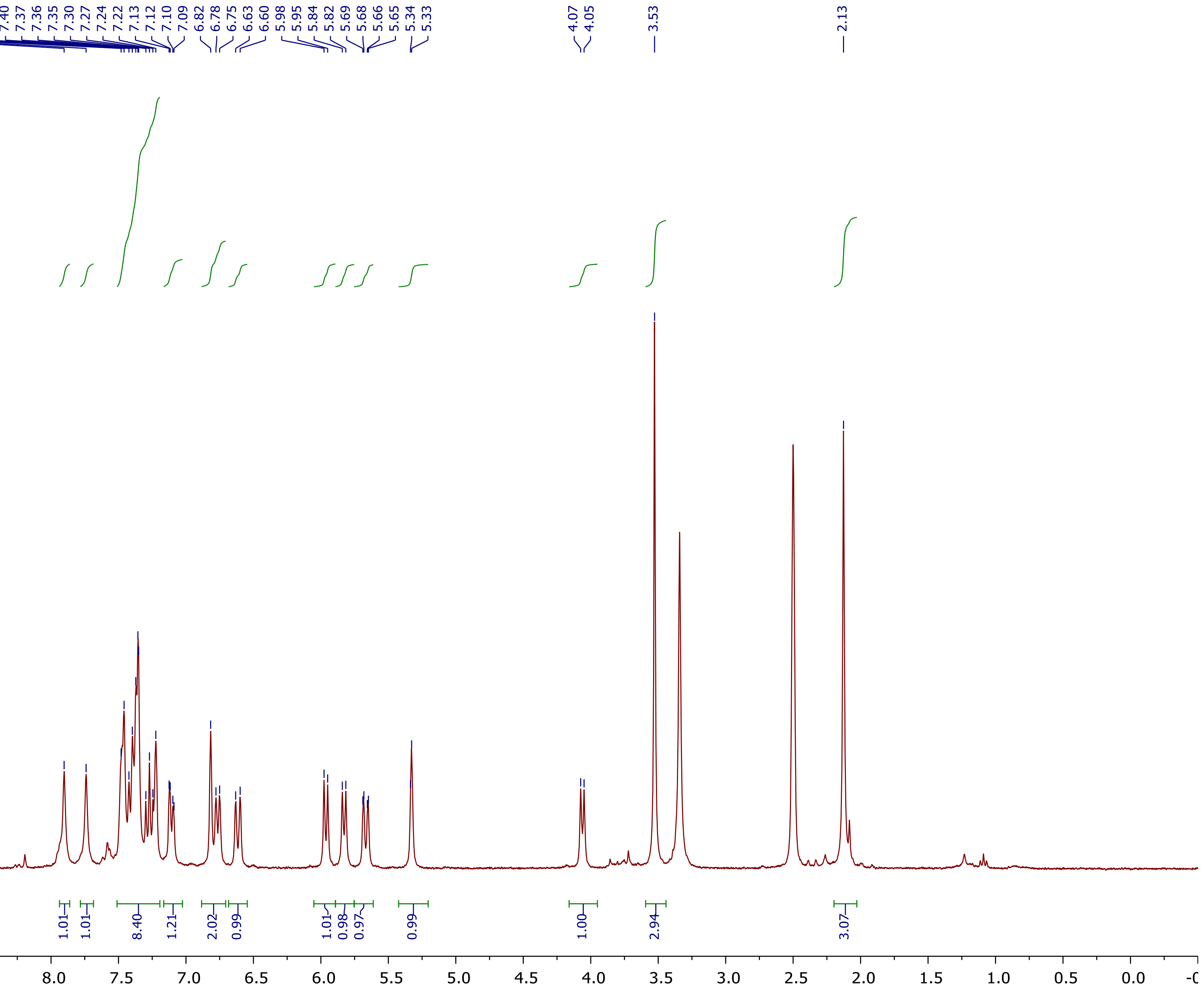

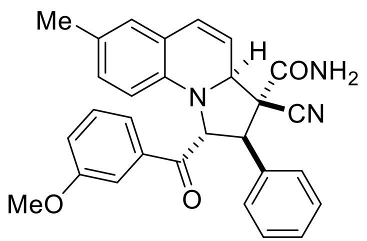

$^{13}C\{^{1}H\}$ **NMR (75 MHz, DMSO-*d*$_6$)**

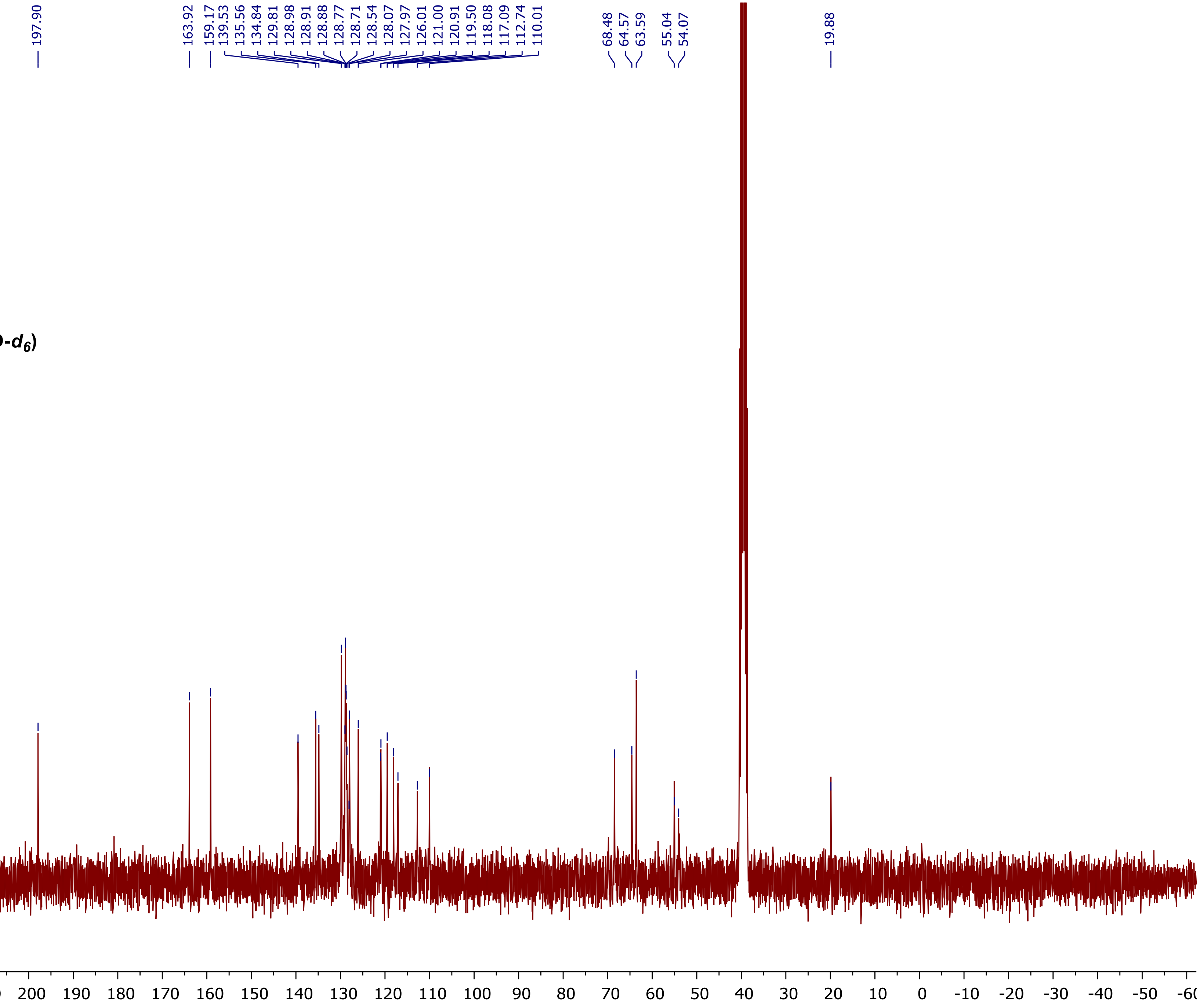

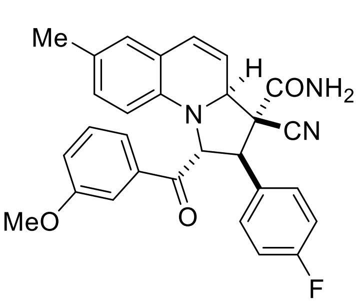

^{1}H NMR (300 MHz, $CDCl_3$)

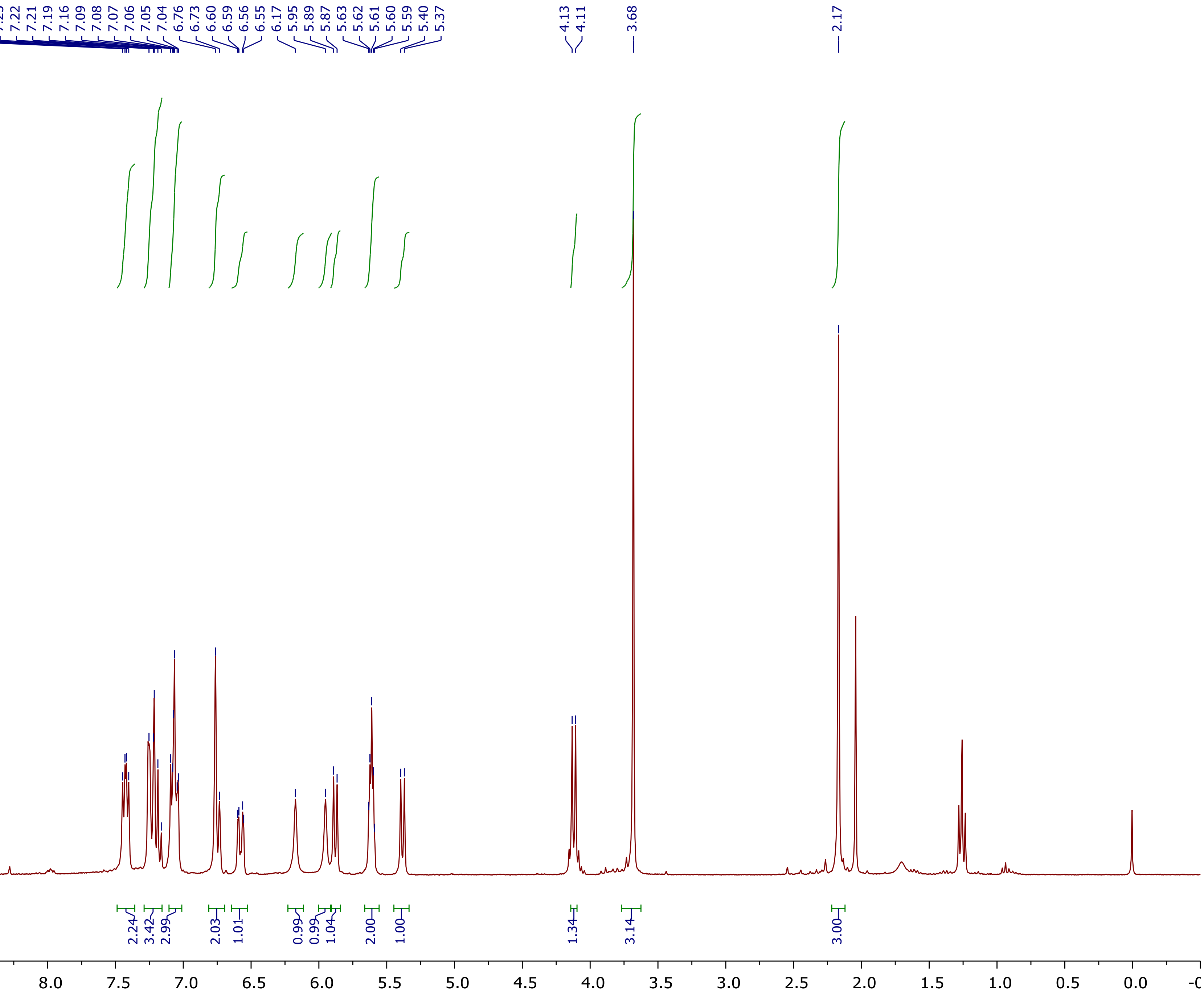

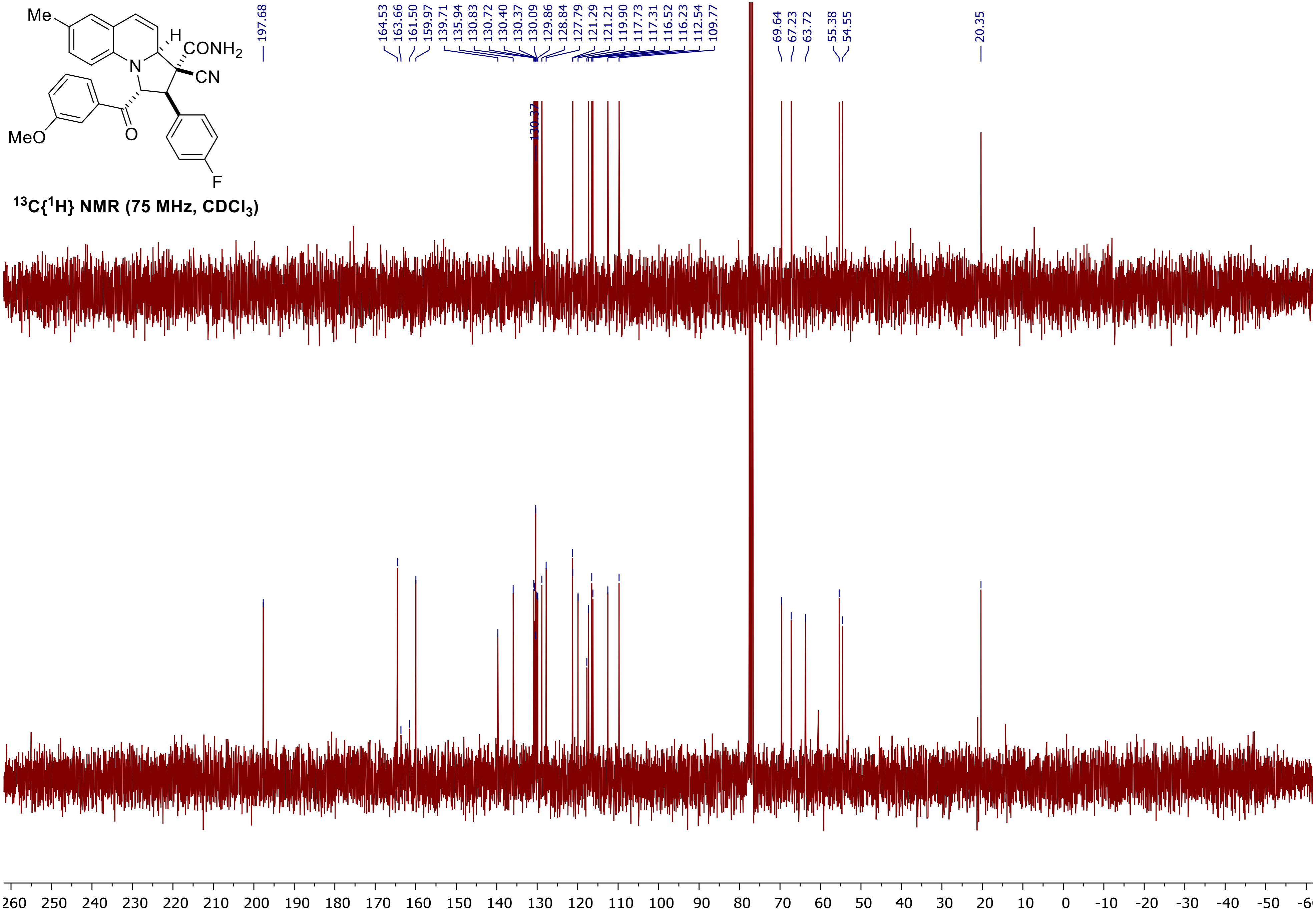
Me
H
CONH2
CN
N
MeO
O
F
13C{1H} NMR (75 MHz, CDCl3)
197.68
164.53
163.66
161.50
159.97
139.71
135.94
130.83
130.72
130.40
130.37
130.09
129.86
128.84
127.79
121.29
121.21
119.90
117.73
117.31
116.52
116.23
112.54
109.77
69.64
67.23
63.72
55.38
54.55
20.35
130.37
260 250 240 230 220 210 200 190 180 170 160 150 140 130 120 110 100 90 80 70 60 50 40 30 20 10 0 -10 -20 -30 -40 -50

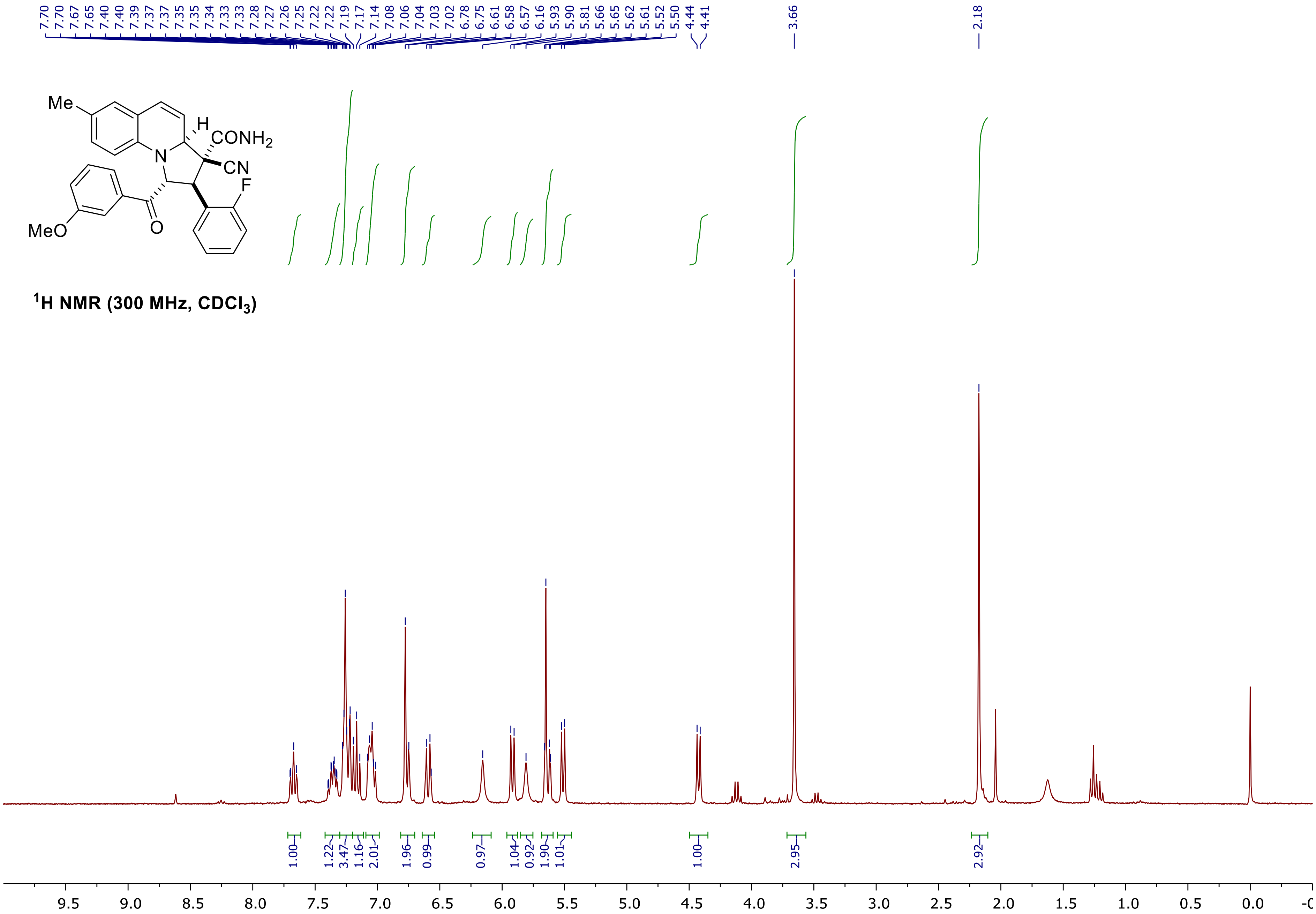

^{1}H NMR (300 MHz, $CDCl_3$)

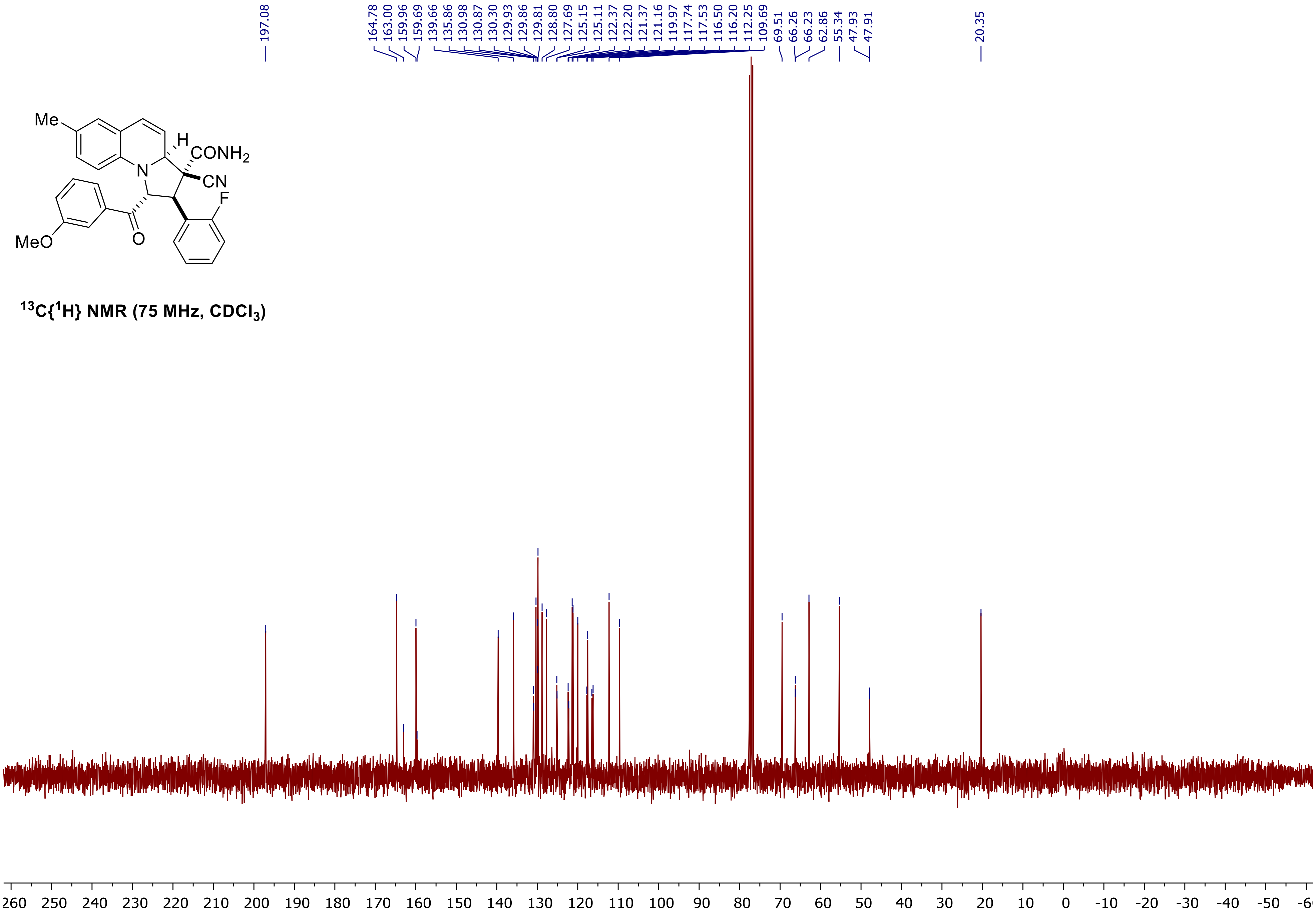
197.08
164.78
163.00
159.96
159.69
139.66
135.86
130.98
130.87
130.30
129.93
129.86
129.81
128.80
127.69
125.15
125.11
122.37
122.20
121.37
121.16
119.97
117.74
117.53
116.50
116.20
112.25
109.69
69.51
66.26
66.23
62.86
55.34
47.93
47.91
20.35
Me
H
CONH2
N
CN
F
MeO
O
13C{1H} NMR (75 MHz, CDCl3)
260 250 240 230 220 210 200 190 180 170 160 150 140 130 120 110 100 90 80 70 60 50 40 30 20 10 0 -10 -20 -30 -40 -50 -6

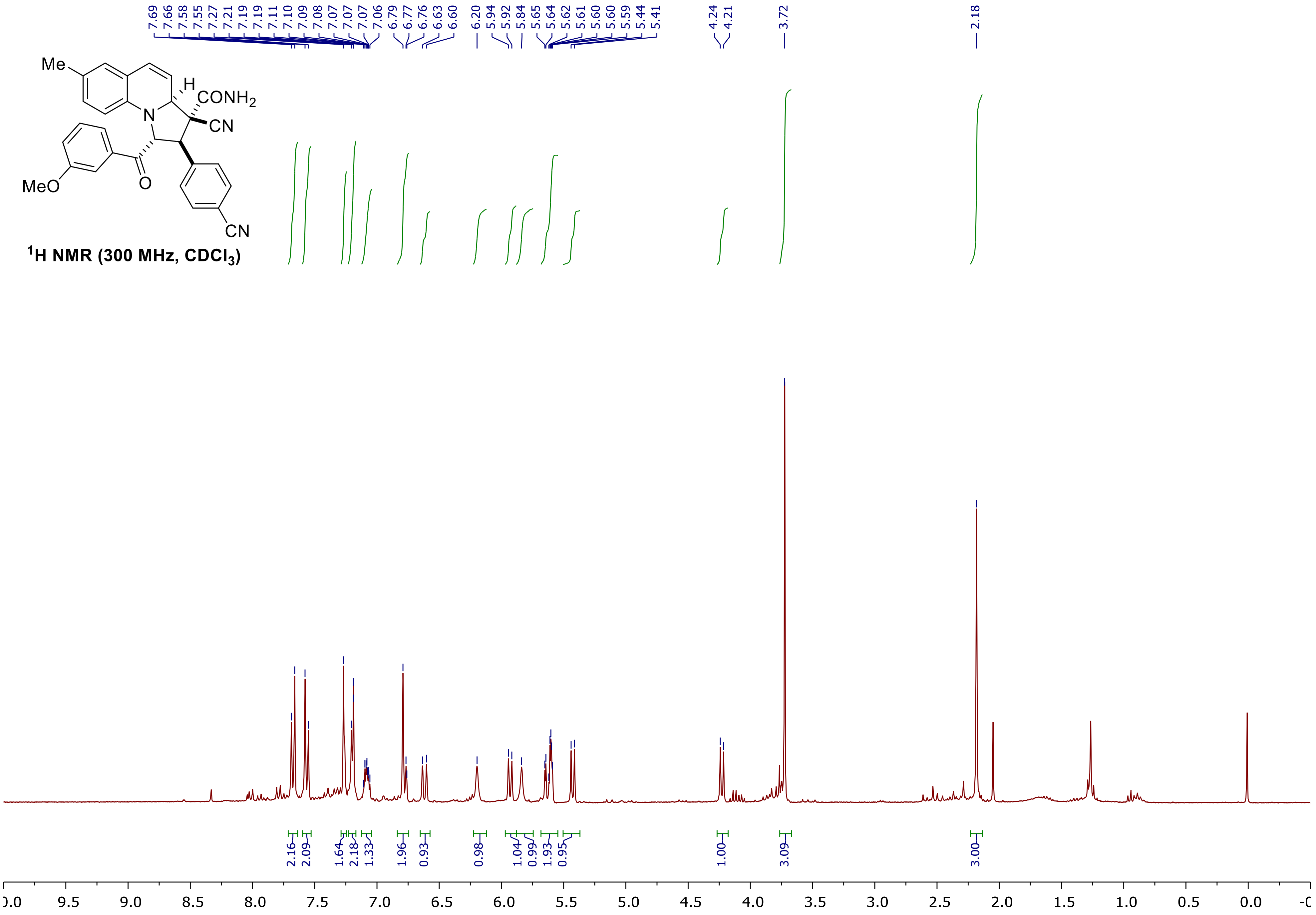

^{1}H NMR (300 MHz, $CDCl_3$)

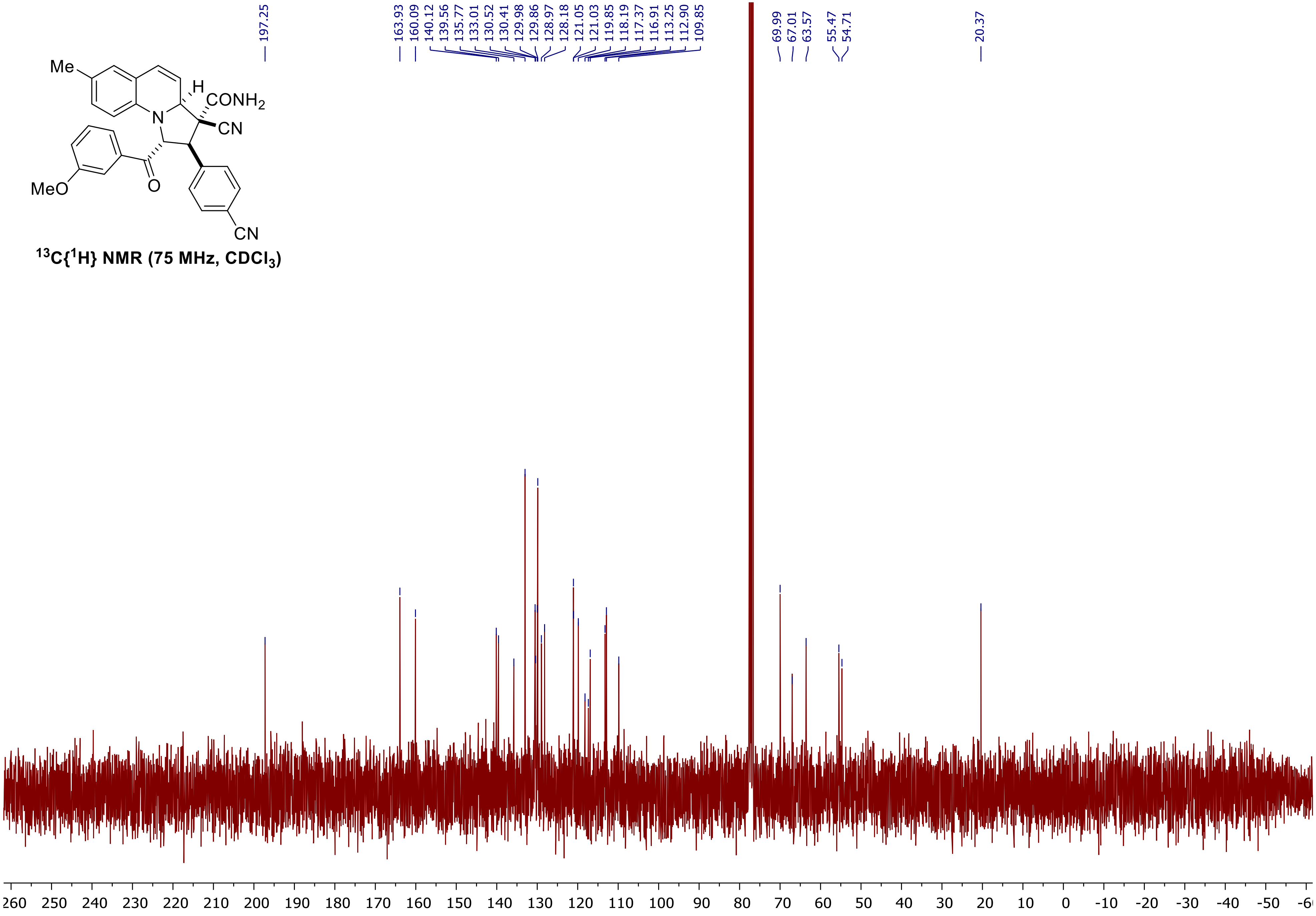

$^{13}C\{^{1}H\}$ NMR (75 MHz, $CDCl_3$)

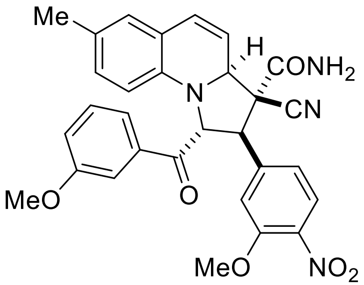

^{1}H NMR (300 MHz, $CDCl_3$)

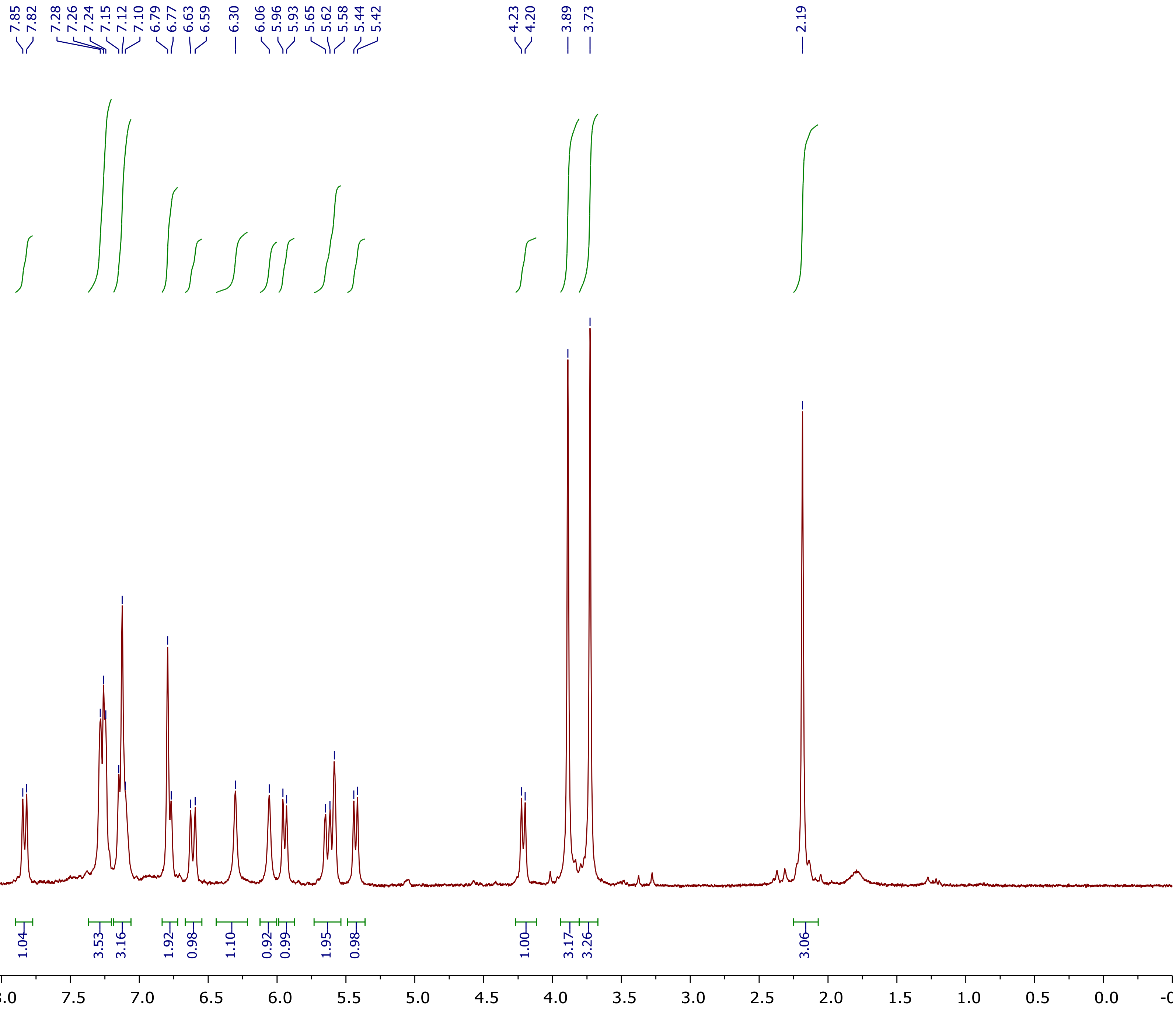

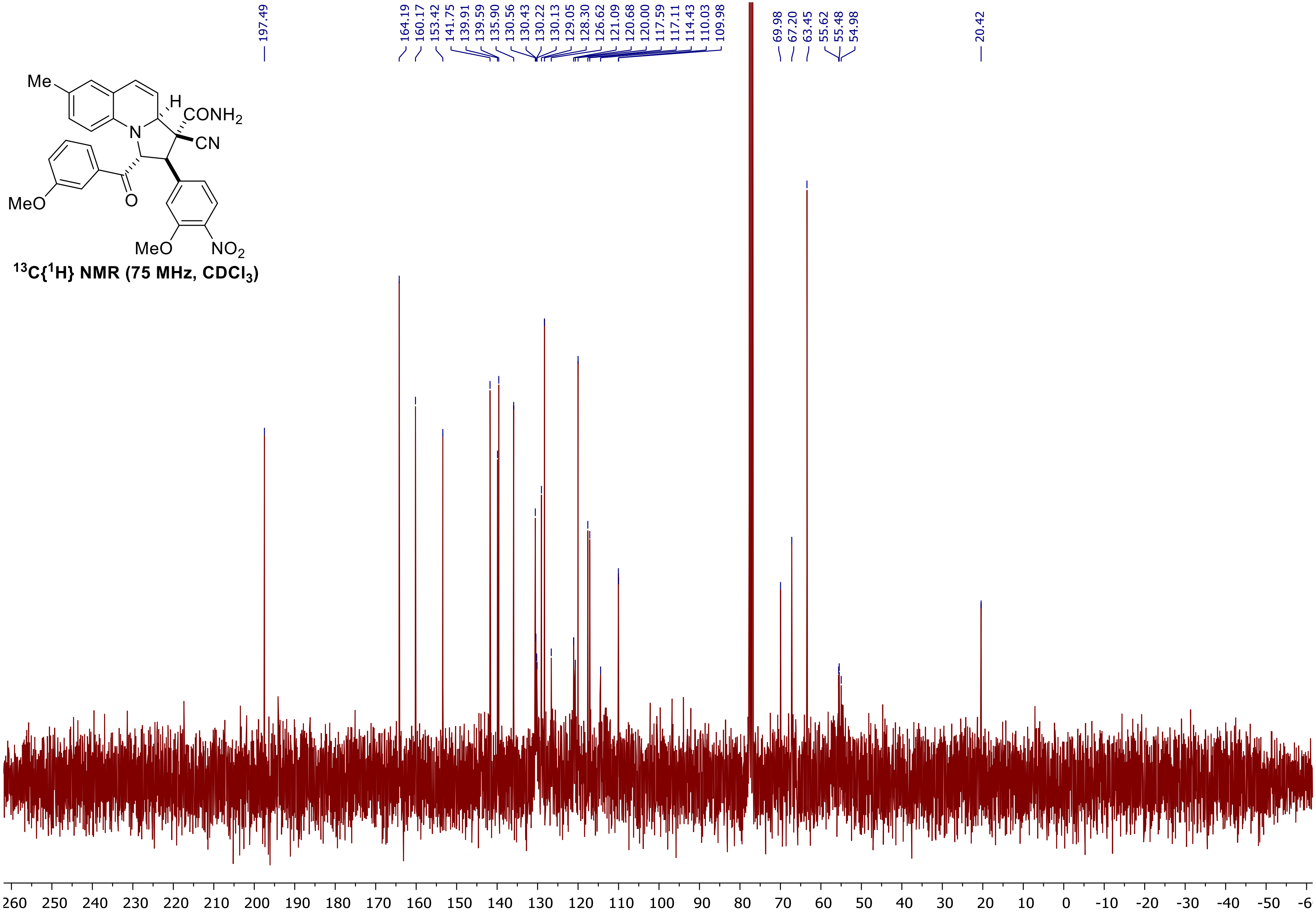
Me
H
CONH2
N
CN
MeO
O
MeO
NO2
13C{1H} NMR (75 MHz, CDCl3)
197.49
164.19
160.17
153.42
141.75
139.91
139.59
135.90
130.56
130.43
130.22
130.13
129.05
128.30
126.62
121.09
120.68
120.00
117.59
117.11
114.43
110.03
109.98
69.98
67.20
63.45
55.62
55.48
54.98
20.42
260 250 240 230 220 210 200 190 180 170 160 150 140 130 120 110 100 90 80 70 60 50 40 30 20 10 0 -10 -20 -30 -40 -50 -6

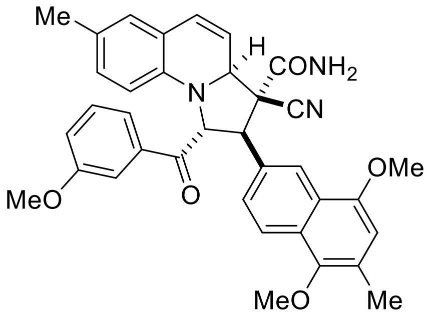

(used in biology studies)

^{1}H NMR (300 MHz, CDCl$_3$)

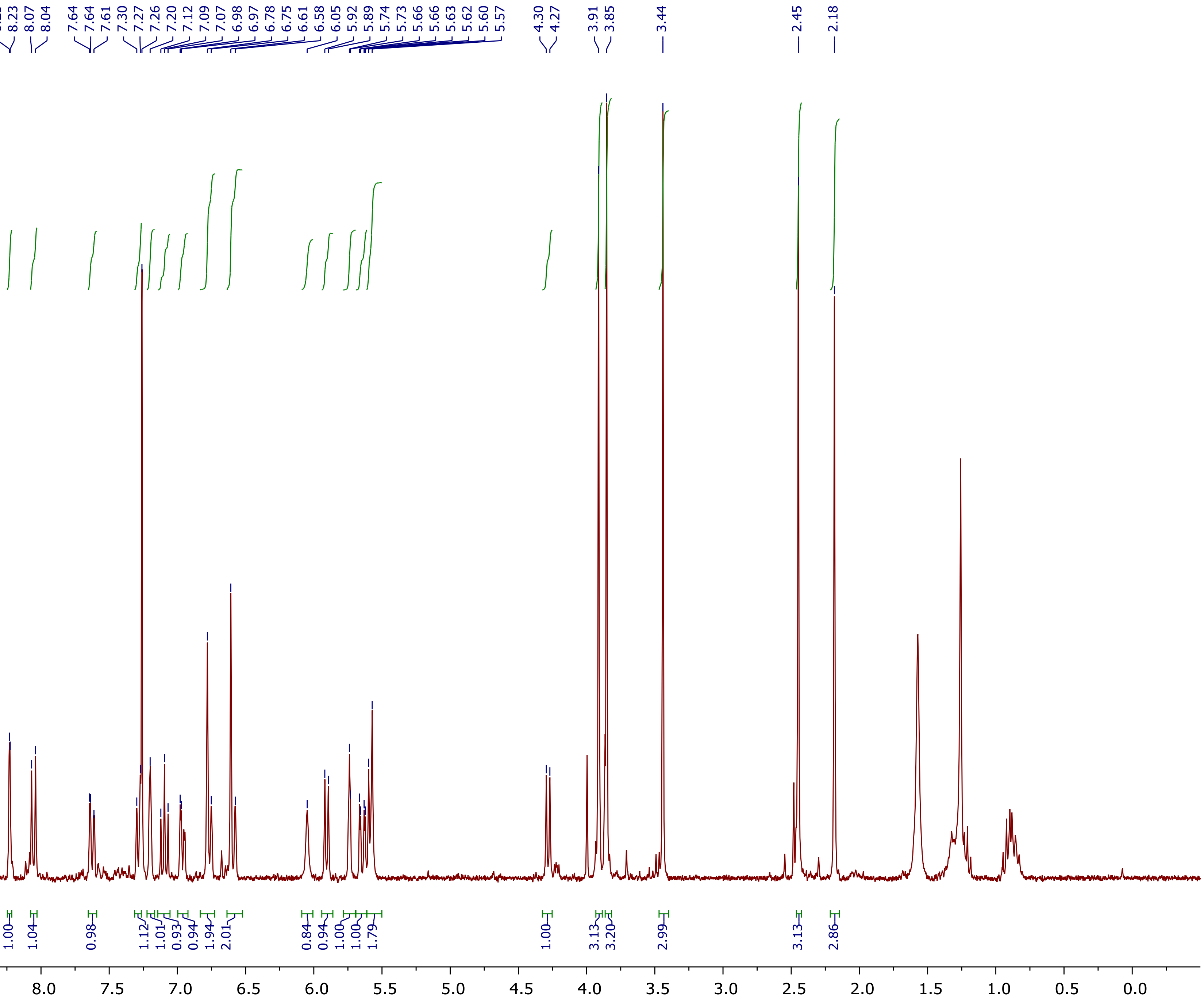

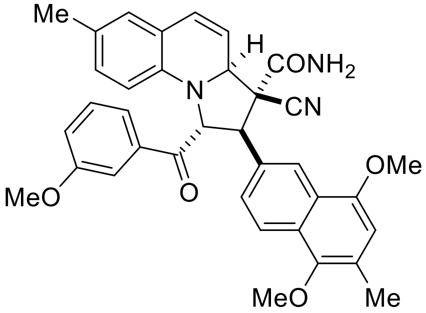

(contaminated with SM)

^{1}H NMR (700 MHz, DMSO-*d*$_6$)

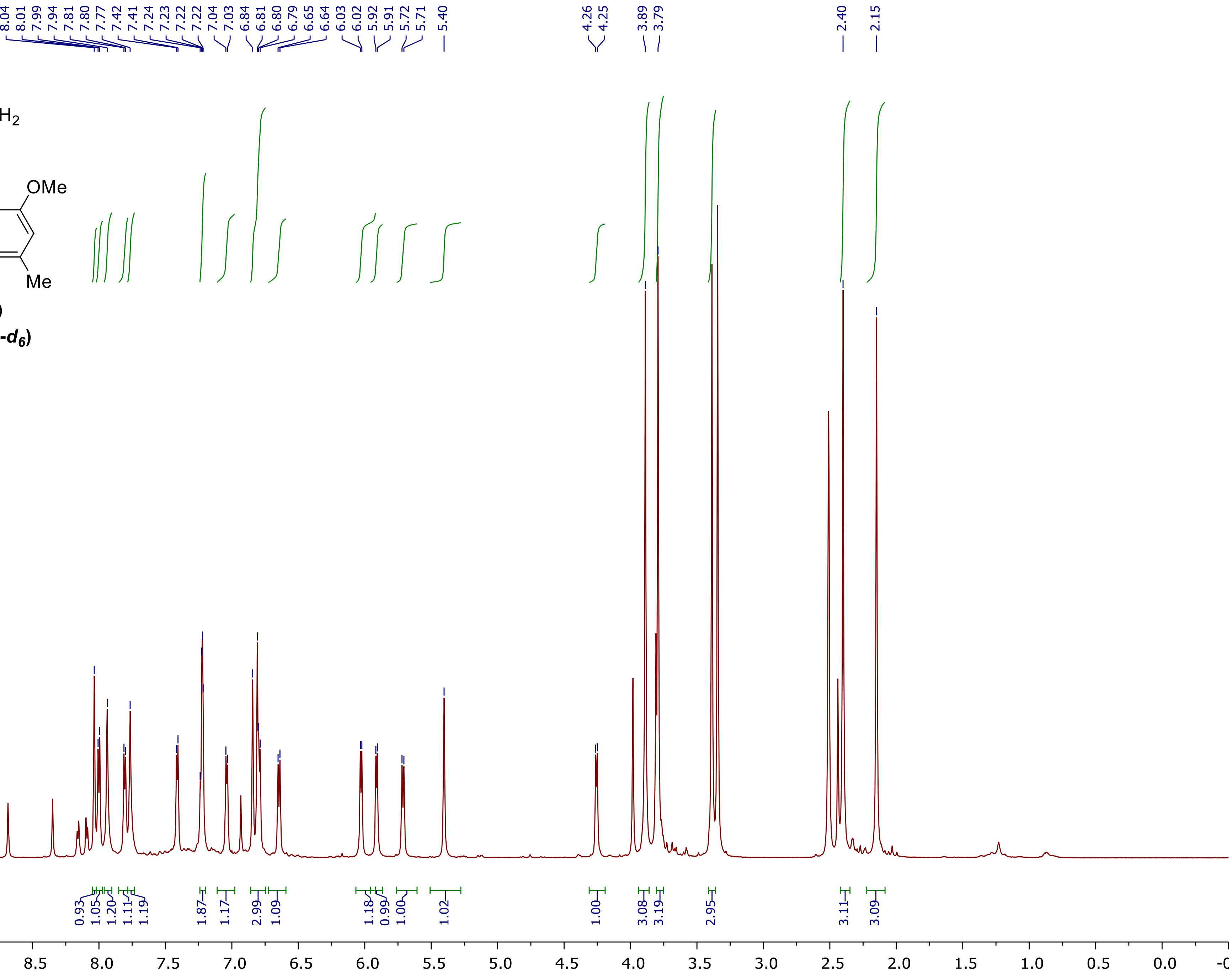

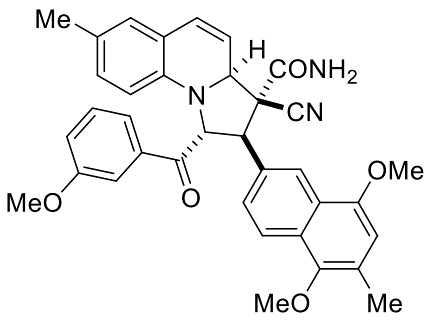

(contaminated with SM)

$^{13}C\{^{1}H\}$ NMR (175 MHz, DMSO-d_6)

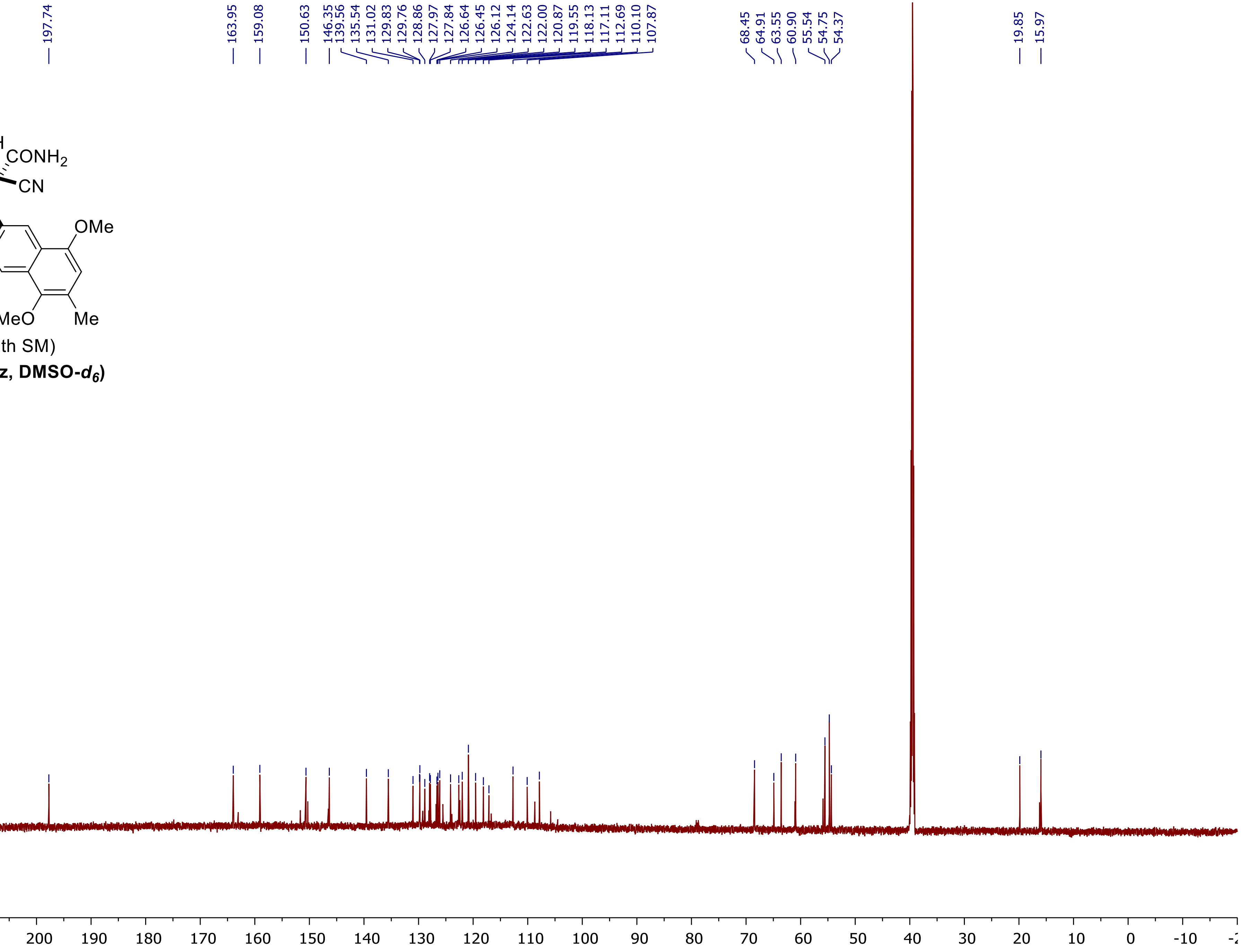

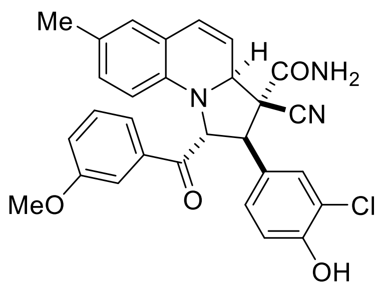

^{1}H NMR (300 MHz, $CDCl_3$)

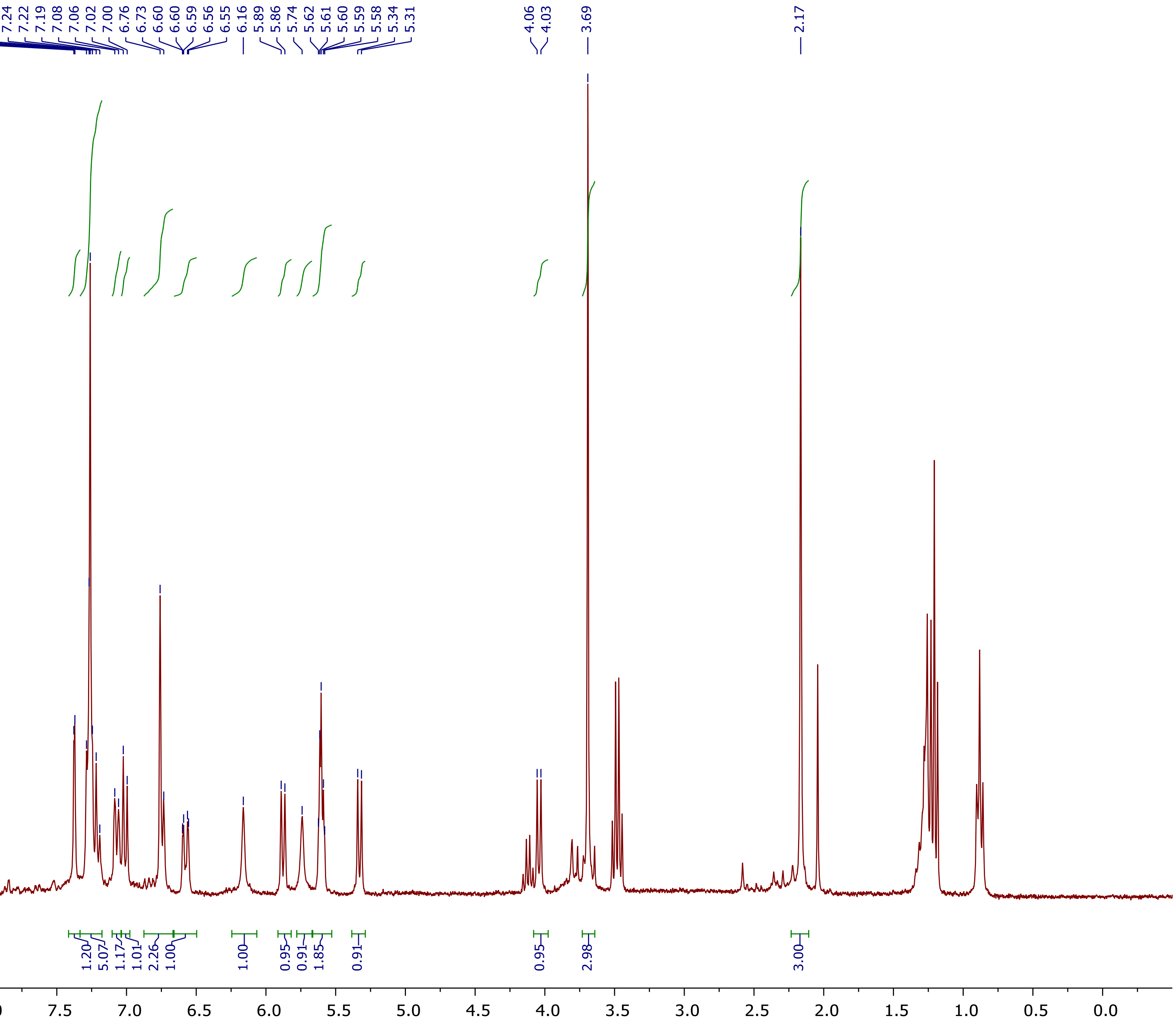

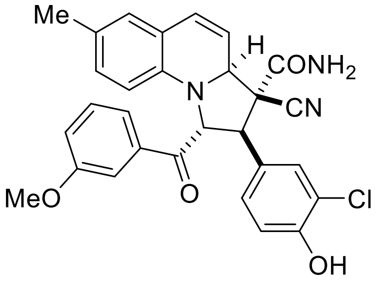

^{13}C NMR (75 MHz, CDCl$_3$)

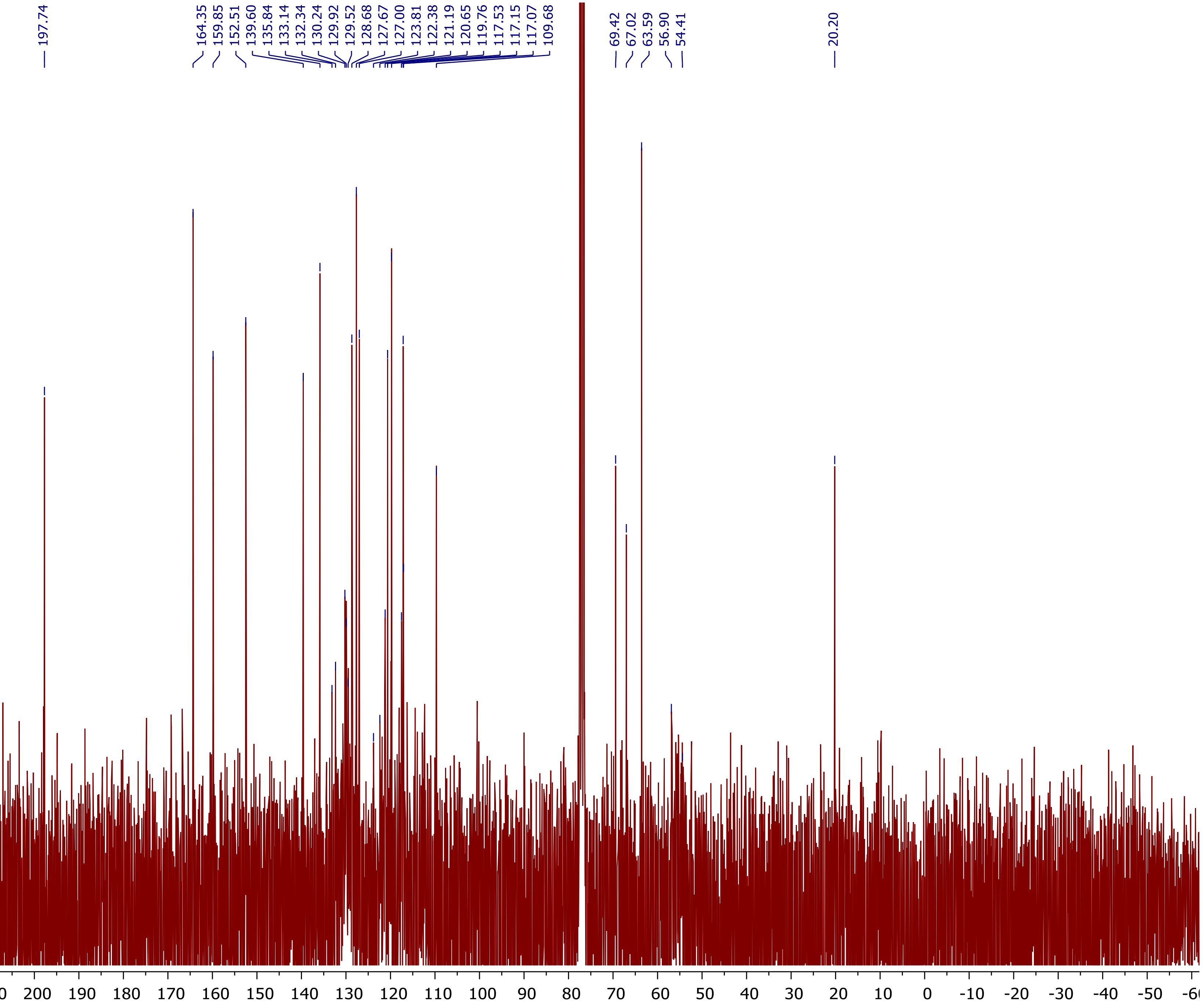

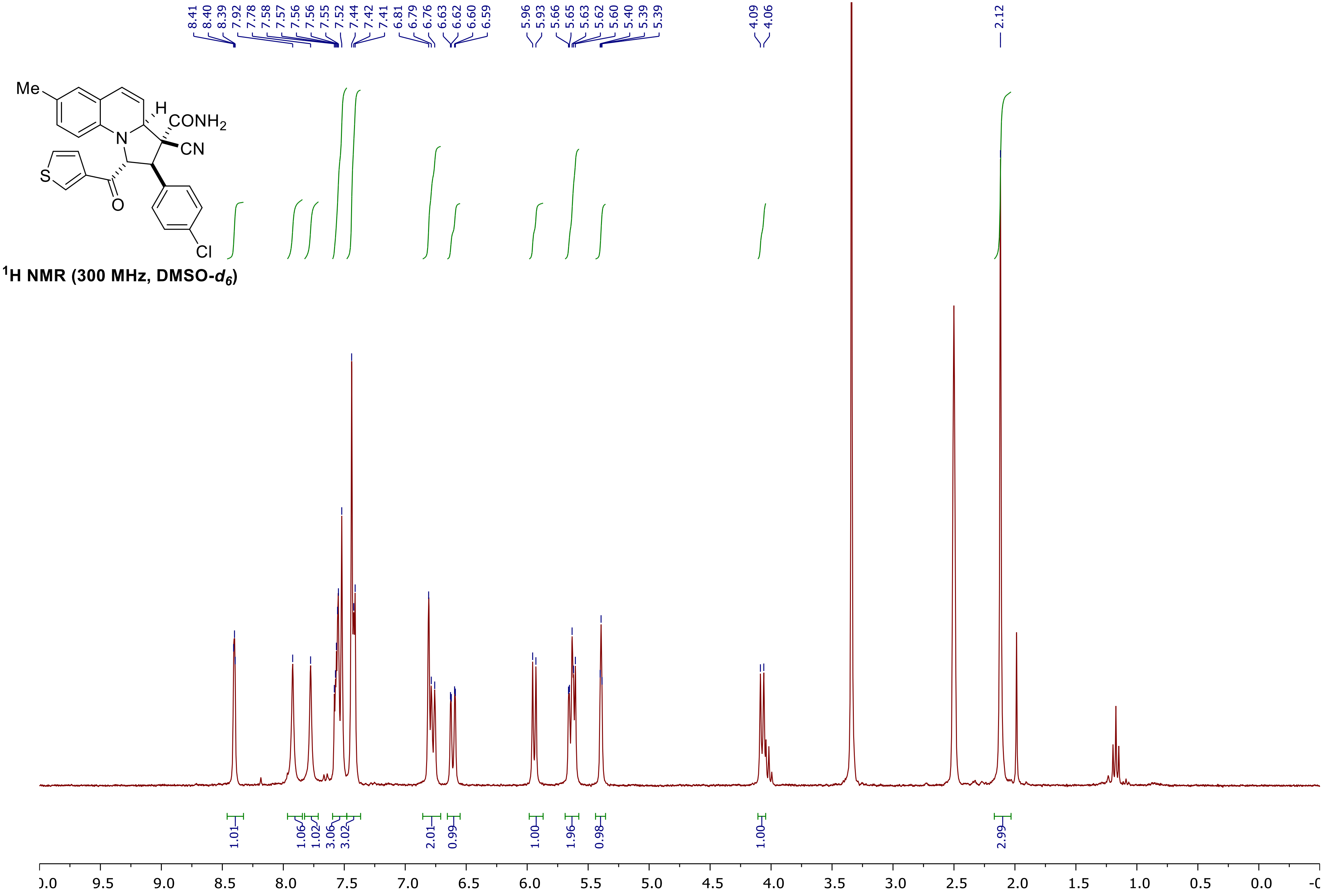
Me
H
CONH2
N
CN
S
O
Cl
1H NMR (300 MHz, DMSO-d6)
8.41
8.40
8.39
7.92
7.78
7.58
7.57
7.56
7.56
7.55
7.52
7.44
7.42
7.41
6.81
6.79
6.76
6.63
6.62
6.60
6.59
5.96
5.93
5.66
5.65
5.63
5.62
5.60
5.40
5.39
5.39
4.09
4.06
2.12
1.01
1.06
1.02
3.06
3.02
2.01
0.99
1.00
1.96
0.98
1.00
2.99
9.5
9.0
8.5
8.0
7.5
7.0
6.5
6.0
5.5
5.0
4.5
4.0
3.5
3.0
2.5
2.0
1.5
1.0
0.5
0.0

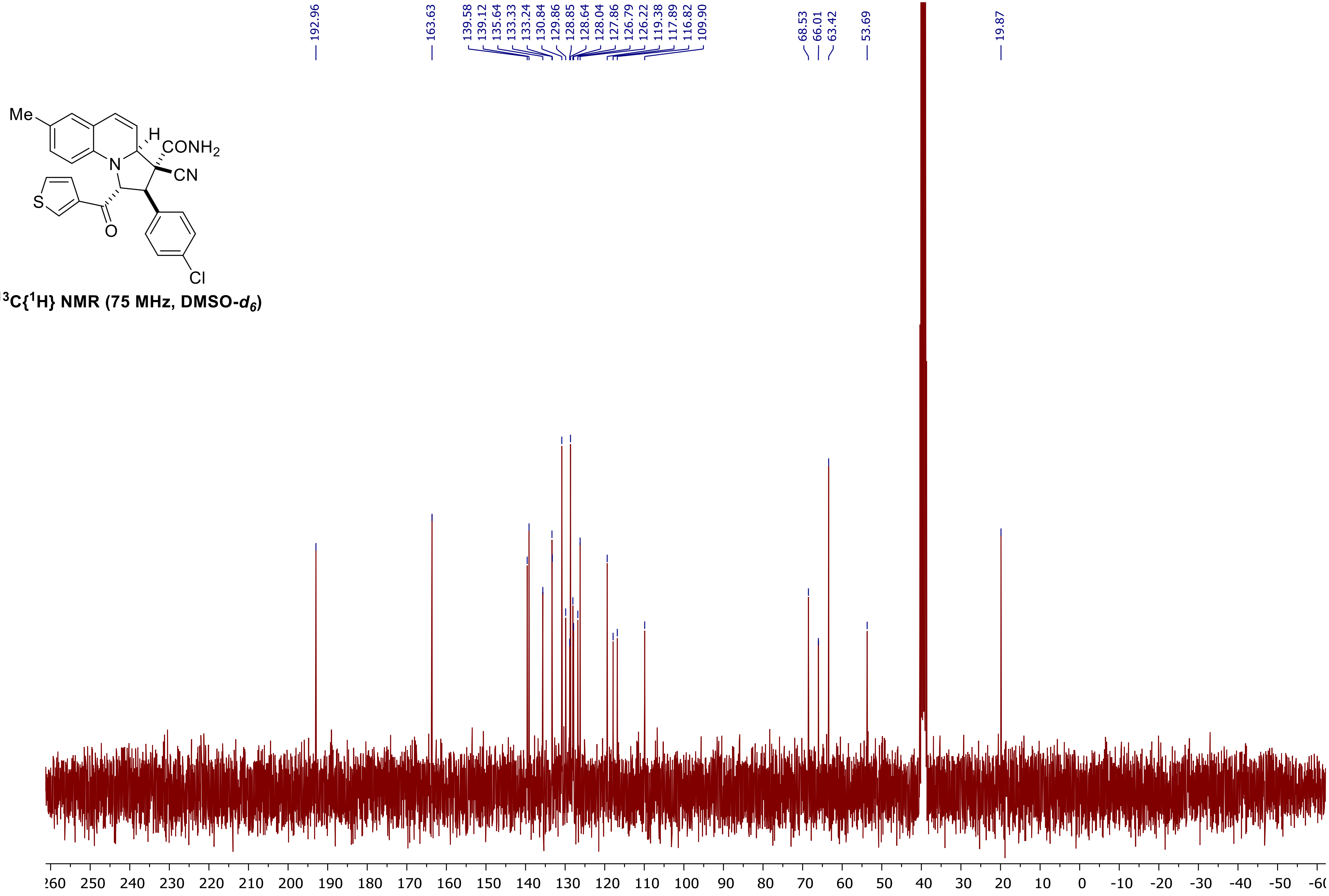

$^{13}C\{^{1}H\}$ NMR (75 MHz, DMSO-d_6)

Determination of the stereochemistry of compounds by X-ray diffraction and comparison of NMR-data

X-ray datasets for **s17_der2** were collected in Kurchatov Centre for Synchrotron Radiation and Nanotechnology using 'Belok' beamline. The intensities of collected reflections were integrated, merged and empirically corrected for absorption using XDS software.[4]

The structures were solved by dual-space algorithm and refined in anisotropic approximation for non-hydrogen atoms against $F^2(hkl)$. Hydrogen atoms of methyl, methylene and aromatic fragments were calculated according to those idealized geometry and refined with constraints applied to C-H bond lengths and equivalent displacement parameters. All structures were solved with the ShelXT[5] program and refined with the ShelXL[6] program. Molecular graphics was drawn using OLEX2[7] program.

CCDC 2291532 contains the supplementary crystallographic data for **s17_der2**. These data can be obtained free of charge from the Cambridge Crystallographic Data Centre via https://www.ccdc.cam.ac.uk/structures.

Crystallographic data for **s17_der2**

Brutto formula	$C_{29}H_{25}N_3O_3$
Formula weight	463.52
Diffractometer	Rayonix SX 165 area detector and Marexperts dtb goniostat
Scan mode	φ scans
Anode [Wavelength, Å]	synchrotron [0.745]
Crystal Dimensions, mm	0.01 × 0.07 × 0.12
Crystal color	yellow
Crystal system	triclinic
a, Å	6.8090(14)
b, Å	10.015(2)
c, Å	17.754(4)
α, °	79.94(3)
β, °	87.68(3)
γ, °	85.00(3)
Volume, Å^3	1187.1(4)
Density, gcm^{-3}	1.297
Temperature, K	100
T_{min}/T_{max}	0.61/0.94
μ, mm^{-1}	0.094

Space group	$P\bar{1}$
Z	2
F(000)	488
Reflections collected	16185
Independent reflections	6446
Reflections (I>2σ(I))	3749
Parameters	361
R_{int}	0.0533
$2\theta_{min}$ - $2\theta_{max}$, °	2.442 - 62.310
wR_2 (all reflections)	0.1496
R_1(I>σ(I))	0.0540
GOF	1.030
ρ_{min}/ρ_{max}, $e\text{Å}^{-3}$	-0.251/0.291

The crystal structure of s17_der2 belongs to the triclinic crystal system, space group P-1. The carbonyl- and cyanogroups are in a mutual pseudo-trans configuration relatively to the C1-C21 bond (Fig. S1). The methoxyphenyl group is disordered over two positions in a 1:1 ratio. In crystals, s17_der2 molecules are assembled into chains due to weak N-H...N bonds between the amino and cyano groups.

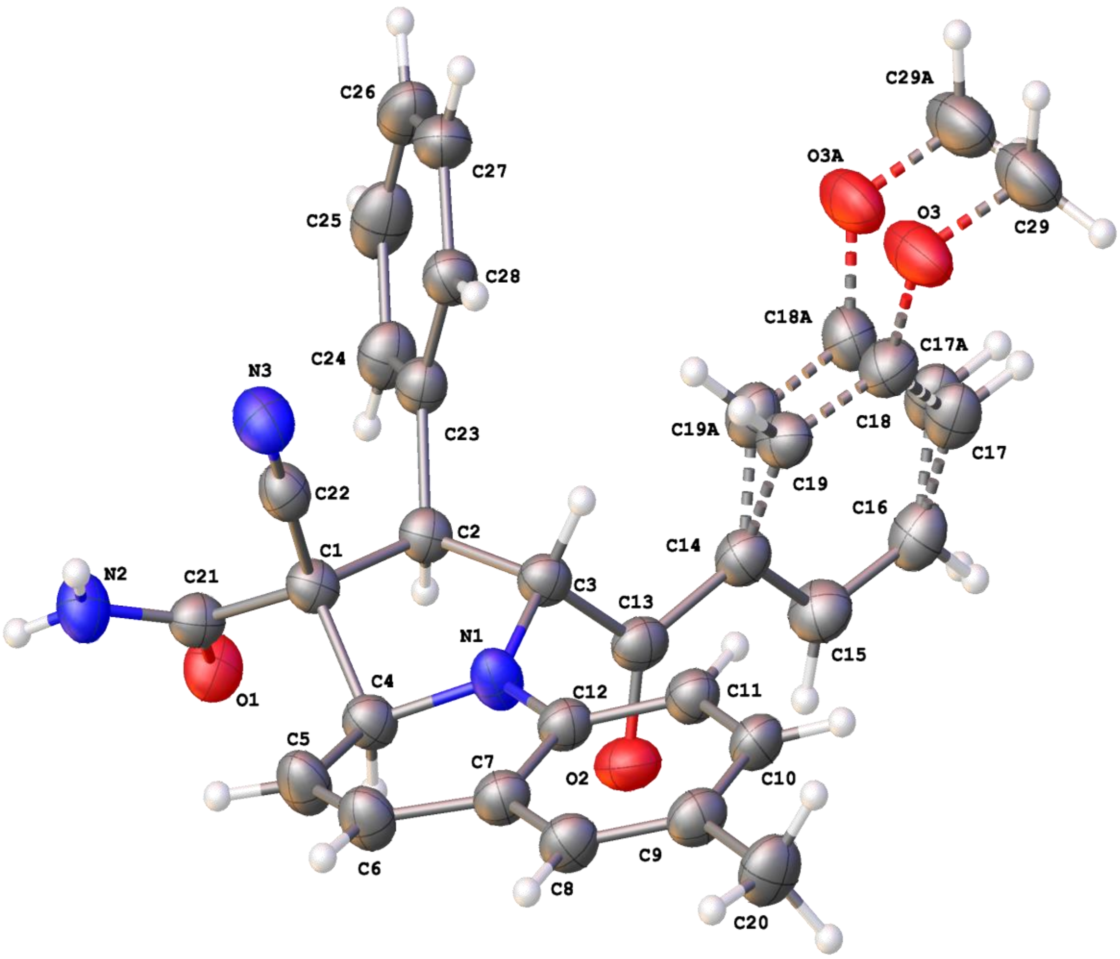

Figure S1. The general view of s17_der2 is presented as anisotropic displacement ellipsoids with a probability of 50%. The disordered part of the molecule is shown by the dotted line.

The relative stereochemical configuration of all other compounds was determined based on the conservatism of the ^{1}H NMR signals in the region of 3.8-7.0. The spectra were measured in CDCl3 or DMSO-d6, for which the overall picture may differ significantly. Therefore, to establish the stereostructure of each compound, its spectrum was compared with the spectrum of s17_der1, the spectra of which were measured in both solvents.

Example of such comparison for s17 is provided below:

1) The proton spectrum of s17_der1 in DMSO-d_6 was compared with the corresponding spectrum of the compound s17_der2, the structure of which was determined by X-Ray analysis. The chemical shifts of all signals are within an error of 0.03 ppm (mostly exactly the same), as well as coupling constants are within an error 0.1 Hz (despite a slight broadening of the s17_der2 spectrum due to relatively poor solubility).

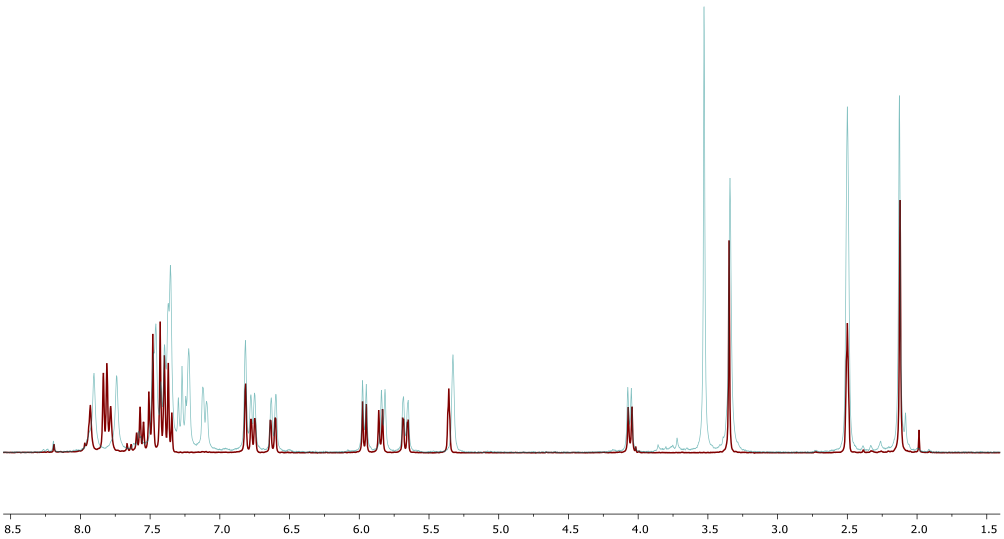

Figures S2. Comparison of the spectra of s17_der1 and s17_der2 in DMSO-d_6. The proton spectrum for s17_der1 is shown in maroon, s17_der2 – in navy blue.

2) The proton spectrum of s17 in $CDCl_3$ was compared with the corresponding spectrum of the compound s17_der1. The chemical shifts of all the signals, except the NH_2 group (which exchanges with the residual water in $CDCl_3$) are within 0.03 ppm error level, as well as coupling constants are within 0.1 Hz error level.

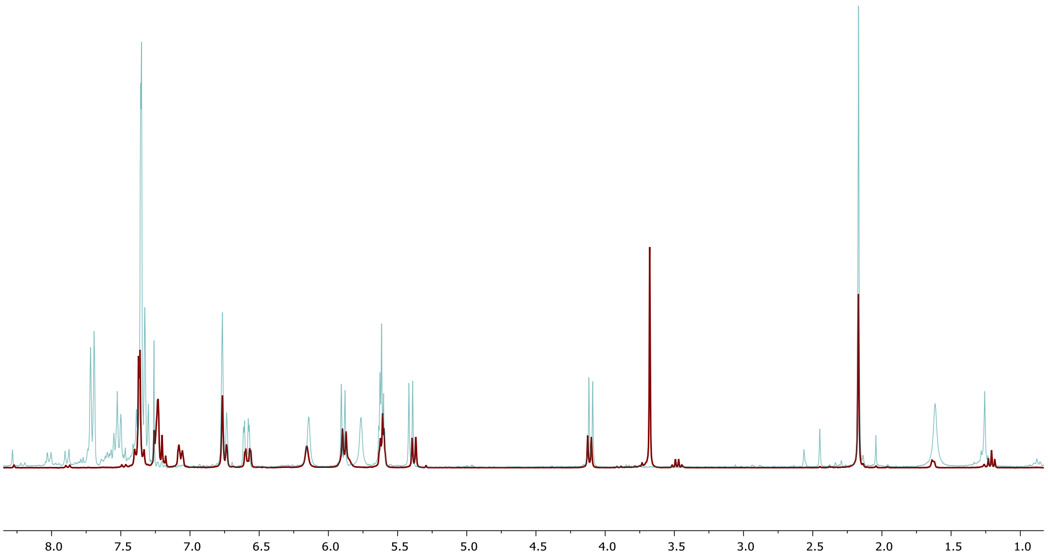

Figure S3. Comparison of the spectra of s17 and s17_der1 in $CDCl_3$. The proton spectrum for s17 is shown in maroon, s17_der1 – in navy blue.

References

(1) Sumanth, G.; Lakshmikanth, K.; Saini, S. M.; Mundhe, P.; Shivaprasad, K.; Chandrashekharappa, S. Phenyl Pyrrolo [1,2-a] Quinolines- Finding of a Key by-Product during Quinolinium Salt Preparation. *J. Mol. Struct.* **2023**, *1273*, 134350.

(2) Kemnitzer, W.; Kuemmerle, J.; Jiang, S.; Zhang, H. Z.; Sirisoma, N.; Kasibhatla, S.; Crogan-Grundy, C.; Tseng, B.; Drewe, J.; Cai, S. X. Discovery of 1-Benzoyl-3-Cyanopyrrolo[1,2-a]Quinolines as a New Series of Apoptosis Inducers Using a Cell- and Caspase-Based High-Throughput Screening Assay. Part 1: Structure-Activity Relationships of the 1- and 3-Positions. *Bioorganic Med. Chem. Lett.* **2008**, *18* (23), 6259–6264.

(3) Gunasekar, R.; Thamaraiselvi, P.; Rathore, R. S.; Sathiyanarayanan, K. I.; Easwaramoorthi, S. Tuning the Electronic Properties of 2-Cyano-3-Phenylacrylamide Derivatives. *J. Org. Chem.* **2015**, *80* (24), 12351–12358.

(4) Kabsch, W. Integration, scaling, space-group assignment and post-refinement. *Acta Cryst.* **2010**, D66, 133–144.

(5) Sheldrick, G.M. SHELXT - Integrated space-group and crystal-structure determination. *Acta Cryst*. **2015**, A71, 3-8.

(6) Sheldrick, G.M. Crystal structure refinement with SHELXL. *Acta Cryst.* **2015**, C71, 3-8.

(7) Dolomanov, O.V., Bourhis, L.J., Gildea, R.J, Howard, J.A.K., Puschmann, H. OLEX2: a complete structure solution, refinement and analysis program., *J. Appl. Cryst.* **2009**, 42, 339-341.
