# Supplementary material for: KuINins as a New Class of HIV-1 Inhibitors That Block Post-Integration DNA Repair
Source: Int J Mol Sci. 2023 Dec 11;24(24):17354. doi: 10.3390/ijms242417354 (PMC10744174; doi:10.3390/ijms242417354)
Supplement: Supplementary file 1 [file ijms-24-17354-s001.zip › sup_section S2_MG.pdf]

Supplementary Section S2

Table S1. Filtering options for selecting compounds for molecular docking.

Parameter	Value
Molecular weight	150 ≤ x ≤ 500
LogP	-5 ≤ x ≤ 5
Hydrogen bond donors	x ≤ 5
Hydrogen bond acceptors	x ≤ 10
Formal charge	2 ≤ x ≤ 2
Rotatable bonds	x ≤ 8
Heavy atoms	15 ≤ x ≤ 50

Table S2. The effect of 100 µM compounds s1-s37 on the complex formation between the recombinant IN and Ku70 proteins

Short name	ChemDiv ID	Structure	Relative amount of IN/Ku70 complex, %
s1	2388-1023	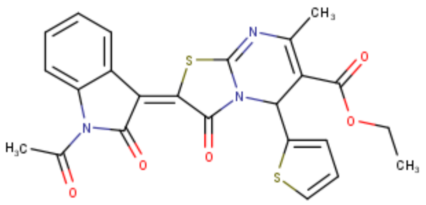	51±7
s2	2388-1024	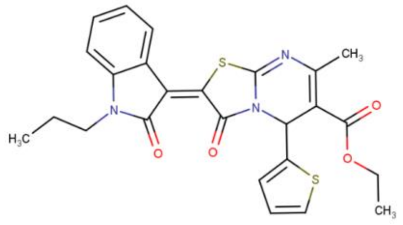	80±12
s3	4340-1060	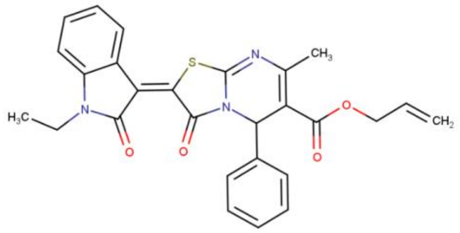	84±8

s4	5645-0002	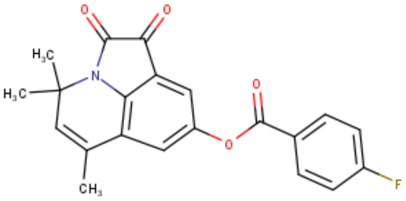	80±5
s5	6528-0171	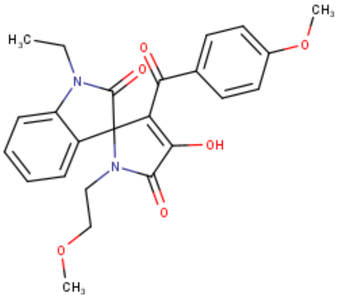	94±6
s6	7954-5114	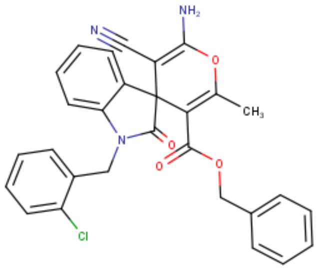	80±10
s7	8006-7560	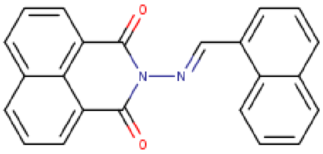	90±5
s8	8009-7811	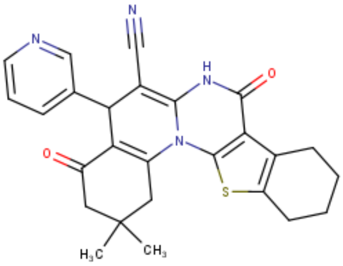	33±4
s9	8009-9264	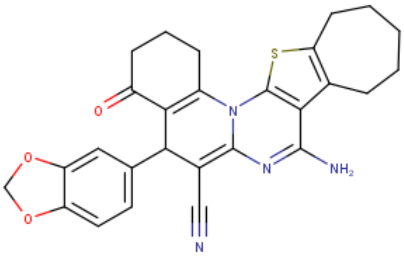	9±2
s10	8013-3218	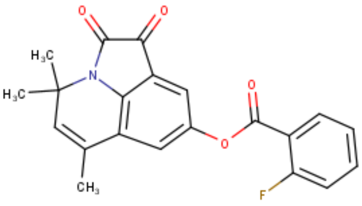	85±4

s11	8013-5834	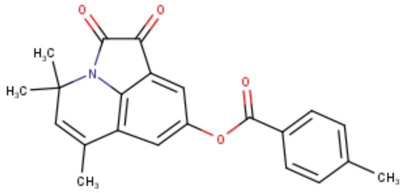	77±6
s12	8015-2552	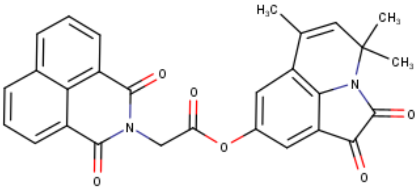	81±5
s13	8015-2553	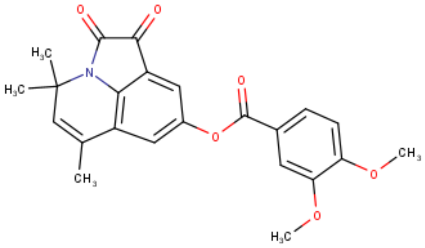	86±8
s14	8015-2560	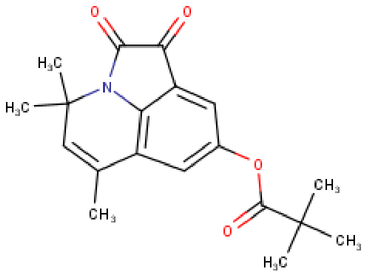	87±10
s15	8016-8353	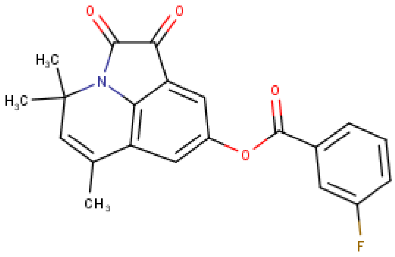	86±6
s16	8017-9121	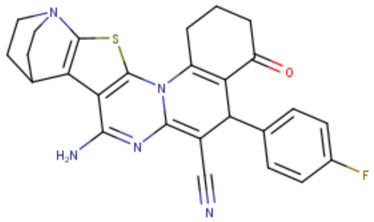	70±6
s17	8018-2468	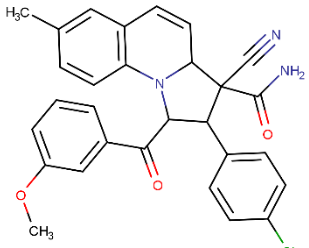	8±2

s18	D063-0985	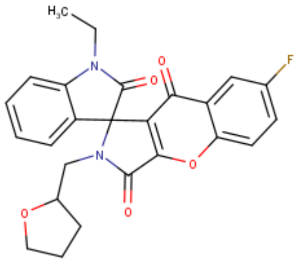	87±9
s19	D436-0418	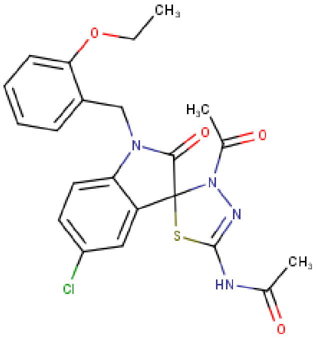	88±8
s20	D436-0419	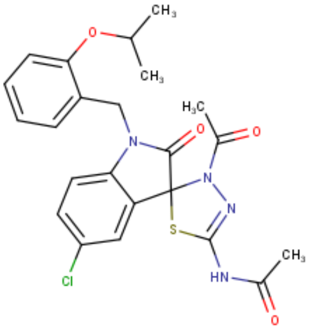	85±4
s21	D477-0395	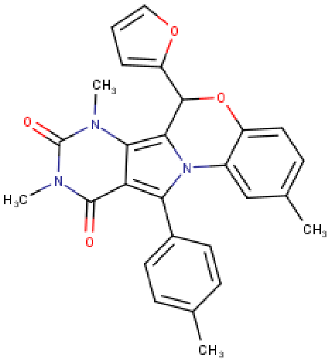	88±7
s22	D718-0015	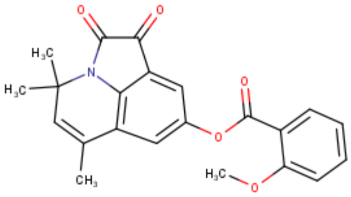	81±5

s23	V028-1401	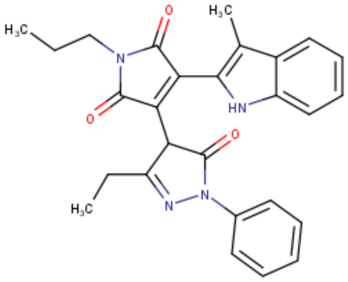	69±7
s24	Y020-0485	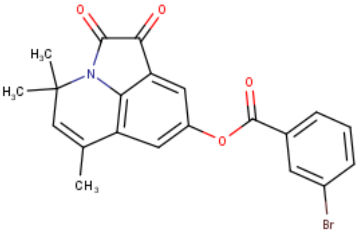	70±4
s25	Y020-7157	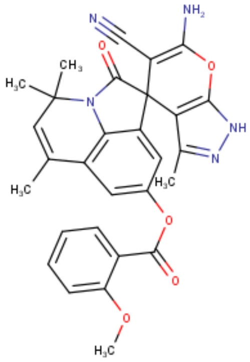	72±8
s26	Y021-1030	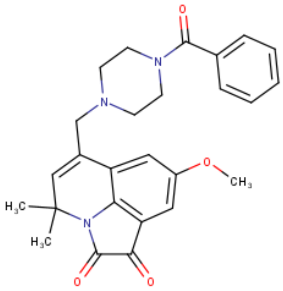	60±7
s27	Y021-1831	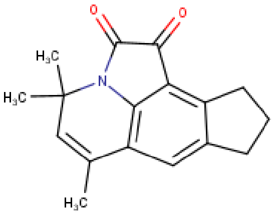	72±6
s28	Y021-2381	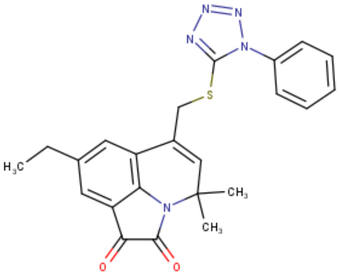	55±4

s29	Y021-2384	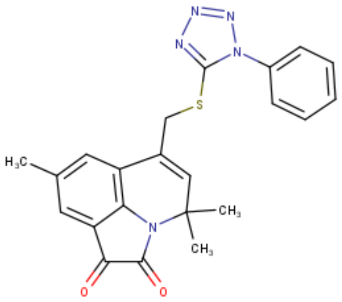	76±6
s30	Y021-2385	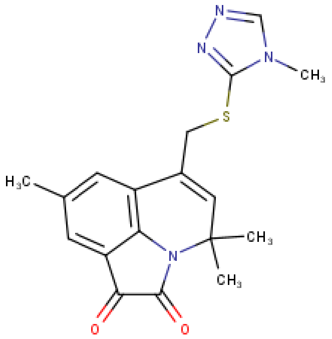	73±7
s31	Y021-2391	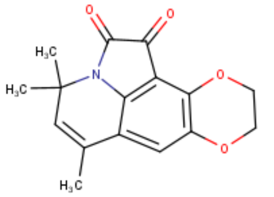	74±10
s32	Y021-2699	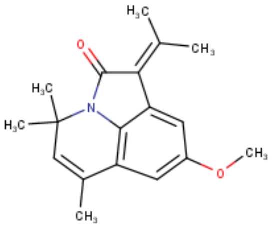	65±4
s33	Y021-2713	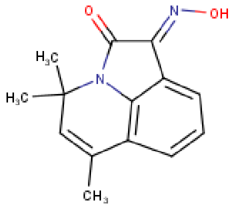	92±8
s34	Y021-2725	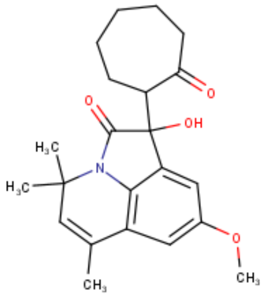	92±6

s35	Y021-2756	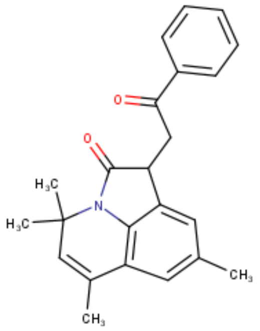	83±7
s36	Y021-2757	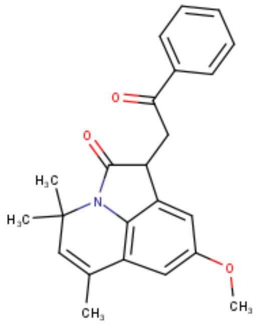	86±9
s37	Y021-2876	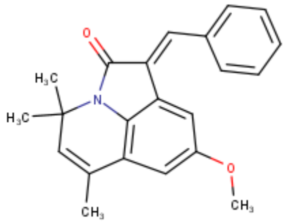	99±8

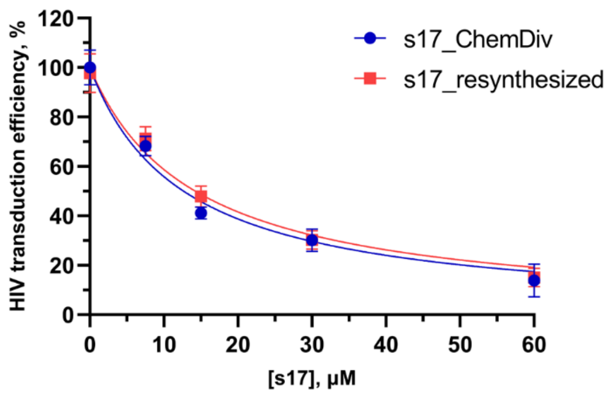

Figure S4. The effect of the commercially available s17 (ChemDiv ID 8018-2468) and its resynthesized variant on the efficiency of 293T cell transduction by HIV-1_wt vector. Mean±SD (n=3) are present.

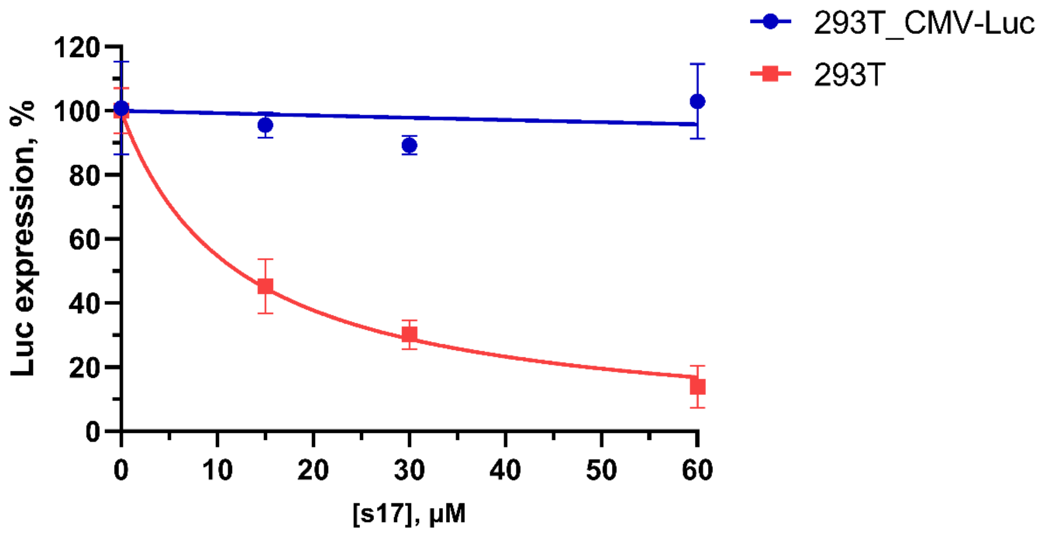

Figure S5. The effect of s17 on firefly luciferase expression from CMV promoter. To obtain 293T_CMV-Luc cells, 293T cells were transduced by HIV_wt vector at MOI 1.0 and grown for 10 days to remove all unintegrated viral DNA. 10 days later, s17 was added to stably transduced cells 293T_CMV-Luc and the luciferase expression level was measured 24 hours post-treatment. In parallel, the effect of s17 on transduction of 293T by HIV_wt was measured. For this purpose, 293T cells were transduced by HIV_wt and simultaneously treated by s17 for 24 h. Luciferase levels were measured 24 hours after transduction. Mean±SD (n=3) are present.

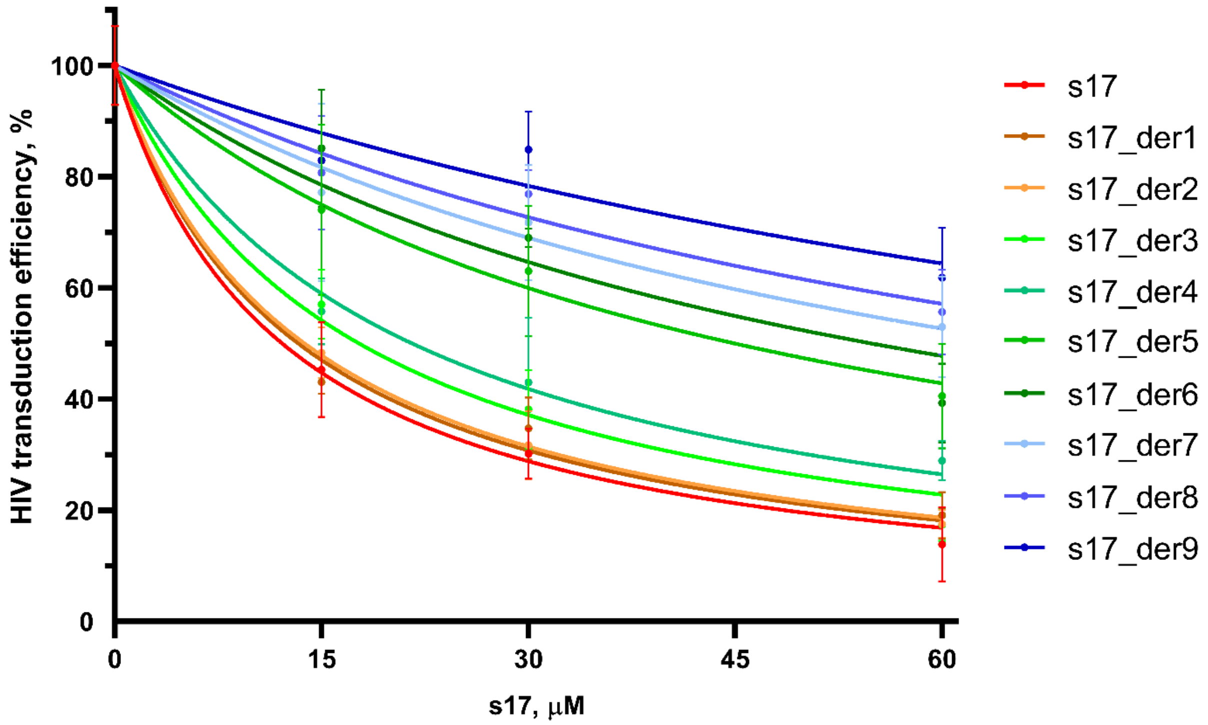

Figure S6. The effect of s17 derivatives with substituted methoxyphenyl or chlorophenyl radicals on the transduction efficiency of 293T cells. 293T cells were transduced with the HIV_wt vector and treated with s17 or its derivatives. After 24 hours, the cells were lysed and the level of luciferase expression was measured. Data were normalized to DMSO treated samples (0 μM of inhibitors). Mean±SD (n=4) are present.
